# From Porphyrins to Versatile Photostable Bacteriochlorins: Triplet-State-Driven Design of Perfluorinated Photosensitizers

**DOI:** 10.1021/acs.jmedchem.6c01282

**Published:** 2026-08-08

**Authors:** Dominik Barczyk, Marta Warszyńska, Paweł Repetowski, Joanna Kuncewicz, Janusz M. Dąbrowski

**Affiliations:** † Faculty of Chemistry, 37799 Jagiellonian University, Kraków 30-387, Poland; ‡ Doctoral School of Exact and Natural Sciences, Jagiellonian University, Kraków 30-348, Poland

## Abstract

Perfluorinated tetrapyrrolic photosensitizers offer a promising platform for photodynamic therapy, yet establishing clear relationships between molecular structure, excited-state behavior, and therapeutic efficacy remains a major challenge. We report a systematic study of porphyrin and bacteriochlorin derivatives designed to elucidate how structural modifications govern photophysical properties and biological performance. Comprehensive spectroscopic analyses, including measurements of triplet-state lifetimes in both solution and micellar formulations, revealed that metalation and macrocycle reduction critically modulate excited-state deactivation pathways and intersystem crossing efficiencies. These effects translate directly into differences in the photodynamic activity. The lead bacteriochlorin exhibits high photostability and prolonged intratumoral retention, together with a pronounced shift toward type I photochemistry, enabling efficient photodynamic activity under hypoxic conditions and resulting in sustained tumor control in long-term *in vivo* studies. This work identifies triplet-state dynamics as a key design parameter linking molecular architecture to biological response and provides a rational basis for the development of next-generation photosensitizers.

## Graphical abstract

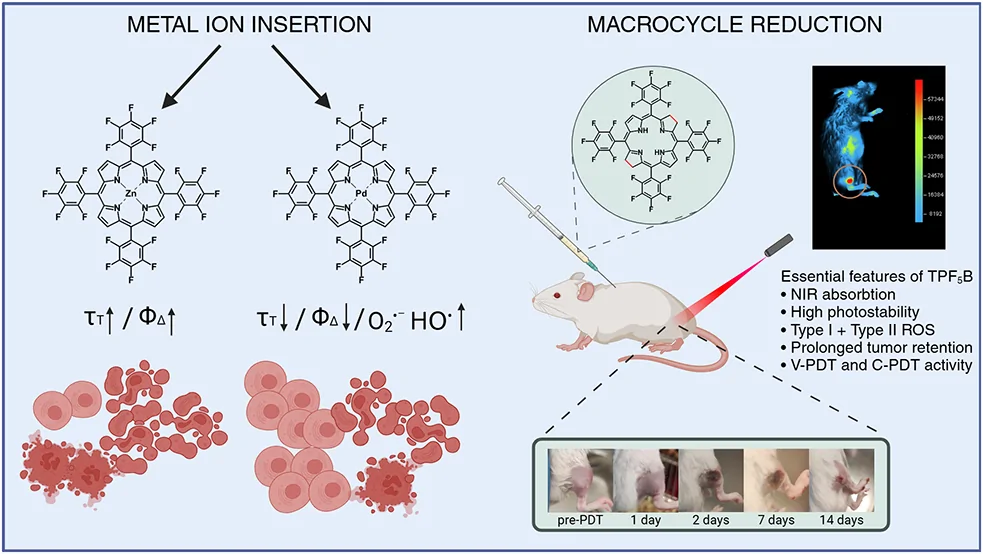

## Introduction

Photodynamic therapy (PDT) is an established treatment modality that relies on the interplay among photosensitizer (PS), molecular oxygen, and light to generate cytotoxic reactive oxygen species (ROS).^1–3^ Upon light activation, PS molecules populate excited states that initiate oxidative processes, leading to cancer cell death, vascular damage, and activation of antitumor immune responses.^4,5^ PDT has demonstrated clinical efficacy in the treatment of various cancers, including skin, lung, prostate, bladder, and head and neck tumors.^6,7^

There is a broad range of tetrapyrrolic compounds currently employed in PDT; however, achieving an optimal balance of key physicochemical and biological properties within a single molecule remains a major challenge. These include not only high selectivity, photostability, and efficient ROS generation but also appropriate amphiphilicity governing partitioning and membrane interactions, as well as low dark cytotoxicity.^8–10^ Numerous porphyrin-based photosensitizers have been developed to address these requirements, but their excitation within the visible spectral region limits their applicability to superficial or easily accessible tumors.^11–13^ Consequently, there is a strong interest in photosensitizers absorbing in the near-infrared (NIR) region, where deeper tissue penetration of light can be achieved, which is critical for effective *in vivo* therapy.^14,15^ Maintaining measurable fluorescence alongside photodynamic activity is also desirable, as it enables monitoring of photosensitizer distribution and supports theranostic applications.

Bacteriochlorins, as reduced porphyrinoids, exhibit intense absorption and emission in the far-red and NIR regions and are therefore attractive candidates for PDT.^14,15^ However, their broader application is often hindered by insufficient photostability, particularly under conditions relevant for cellular-targeted PDT (C-PDT, drug-to-light interval (DLI) ≈ 24 h). As a result, bacteriochlorins have been more commonly explored in vascular-targeted PDT (V-PDT), where short DLIs (typically minutes) favor vascular damage over direct tumor cell killing. Previous studies have shown that structural modifications, including the introduction of halogen substituents and sulfonamide functionalities, can improve photostability and modulate biological performance.^16,17^ Despite these advances, bacteriochlorin-based PSs still rarely combine adequate photostability, favorable excited-state dynamics, and consistent efficacy across distinct therapeutic modalities. Importantly, a clear relationship between molecular structure, triplet-state lifetimes, and *in vivo* therapeutic efficacy remains insufficiently understood.

In this study, we address these challenges through rational structural modification of tetrapyrrolic photosensitizers aimed at enhancing photostability and controlling excited-state properties. Fully fluorinated phenyl rings were retained at the *meso-* positions of the porphyrin core, providing steric protection against oxidative degradation and improving overall stability. Moreover, the electron-withdrawing character of the fluorine substituents promotes intersystem crossing (ISC) via spin–orbit coupling (SOC), enhancing ROS generation.^18^

To further modulate excited-state behavior, the porphyrin core was coordinated with Zn^2+^ or Pd^2+^ ions, which alter SOC and influence ISC efficiency, triplet-state formation, and other deactivation pathways, ultimately affecting the balance among fluorescence, nonradiative decay, and ROS generation.^19,20^ These effects are accompanied by changes in the electronic structure, including hypsochromic shifts of the lowest-energy Q bands, which may influence light-harvesting efficiency and photodynamic performance. Finally, to extend absorption into the near-infrared region and improve tissue penetration, the porphyrin scaffold was reduced to the corresponding bacteriochlorin.^21–23^ This transformation enables NIR excitation but is often accompanied by reduced photostability, typically addressed by introducing bulky substituents such as sulfonamide groups. However, such modifications frequently lead to atropisomerism arising from restricted rotation of peripheral aryl groups, resulting in conformational heterogeneity that complicates purification and reproducibility. In this approach, simplification of peripheral substitution minimizes atropisomer formation while preserving photostability, facilitating purification, scalability, and batch-to-batch consistency. This design strategy enables a systematic investigation of how targeted structural modifications govern triplet-state dynamics, photostability, and the balance between type I and type II photochemical pathways, ultimately shaping photodynamic efficacy under biologically relevant conditions.

## Results and Discussion

### Synthesis

The synthetic strategy enables systematic modification of the tetrapyrrolic core through metalation and macrocycle reduction (Scheme 1). 5,10,15,20-Tetrakis­(pentafluorophenyl)­porphyrin (TPF_5_P) was prepared via condensation of pyrrole with pentafluorobenzaldehyde in dichloromethane under an inert atmosphere, catalyzed by BF_3_·OEt_2_ and followed by oxidation with DDQ.^24^ Metalation with zinc and palladium was performed using the corresponding metal acetates in anhydrous DMF at elevated temperature (150 °C), affording the corresponding metalloporphyrins TPF_5_P Zn and TPF_5_P Pd. Reaction progress was monitored by electronic absorption spectroscopy.^25^ To access the NIR-absorbing derivative, the porphyrin scaffold was further transformed into the corresponding bacteriochlorin (TPF_5_B) via solvent-free reduction using *p*-toluenesulfonyl hydrazide under vacuum and inert atmosphere (140 °C, porphyrin/hydrazide ratio 1:40).^26–28^ The crude product was purified by preparative TLC (ethyl acetate/*n*-hexane, 1:10). This modular approach enables direct comparison of free-base porphyrin, metalloporphyrin, and bacteriochlorin derivatives, providing a consistent platform to assess how structural modifications affect excited-state properties and photodynamic performance.

**Scheme 1:**
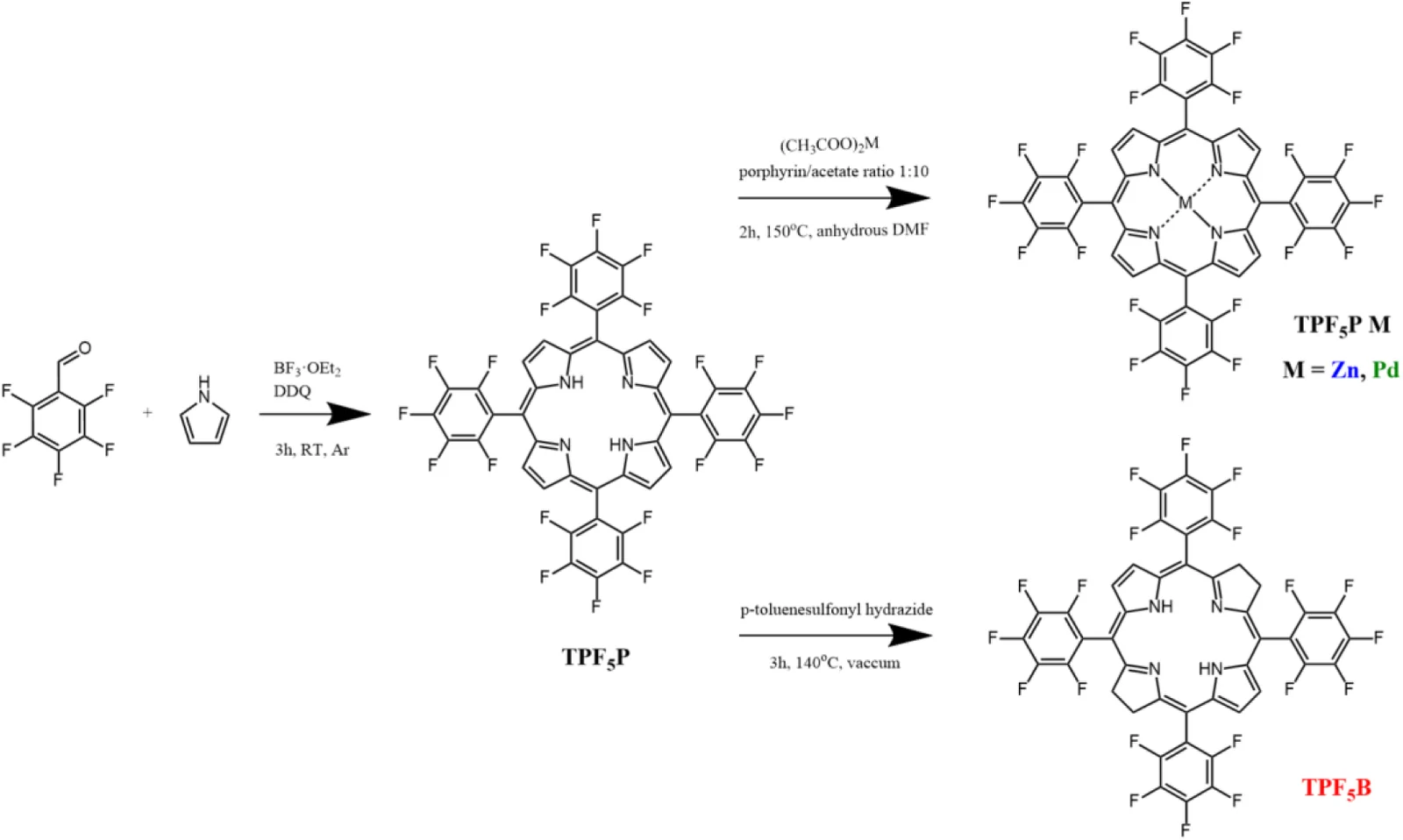
Synthetic Routes to *Meso*-Substituted Perfluorinated Tetrapyrroles, Including Metalation (TPF_5_P M) and Reduction of TPF_5_P to Bacteriochlorin (TPF_5_B)

### Preparation of Pluronic P123 Micelles

Because micellar formulation can strongly influence photosensitizer dispersion, stability, and biological performance, the investigated compounds were incorporated into Pluronic P123 micelles using the thin-film hydration method. The resulting formulations were characterized in terms of encapsulation efficiency, hydrodynamic diameter, ζ-potential, and stability over time.

All investigated photosensitizers were efficiently incorporated into Pluronic P123 micelles with encapsulation efficiencies ranging from 75.4% to 92.3% (Table 1). The highest encapsulation efficiency was observed for TPF_5_B, indicating favorable accommodation of bacteriochlorin within the Pluronic P123 micellar environment. In contrast, TPF_5_P Pd showed the lowest loading efficiency, which may reflect a less favorable molecular packing or weaker compatibility with the micellar core.

**Table 1:** Physicochemical Parameters of Photosensitizer-Loaded P123 Micelles, Including Encapsulation Efficiency, ζ-Potential, Particle Size, and *n*-Octanol/PBS Distribution Coefficient at pH 7.4 (logD_7.4_)

Name	Encapsulation Efficiency [%]	ζ-potential [mV]	Size [nm]	LogD_7.4_
**TPF**_**5**_ **P**	78.3	–15.31	17.7	2.18
**TPF**_**5**_ **P Zn**	88.5	–8.29	20.5	0.35
**TPF**_**5**_ **P Pd**	75.4	–26.83	20.5	-
**TPF**_**5**_ **B**	92.3	–15.00	32.3	1.06

Hydrodynamic diameters determined by dynamic light scattering (DLS) were within the nanoscale range (Figure 1a-d), confirming the formation of small micellar assemblies. TPF_5_P-loaded micelles displayed the smallest, most abundant diameter (17.7 nm), whereas TPF_5_B-loaded micelles formed larger assemblies (32.3 nm), suggesting that macrocycle reduction and the resulting changes in molecular shape may influence the organization within the carrier. Because encapsulation efficiency in Pluronic P123 micelles is governed not only by lipophilicity but also by molecular geometry, aggregation tendency, packing within the hydrophobic PPO domain, and compatibility with the hydrated PEO corona, apparent *n*-octanol/PBS distribution coefficients determined at pH 7.4 (logD_7.4_) are included in Table 1 as supportive descriptors of formulation behavior.

**Figure 1:**
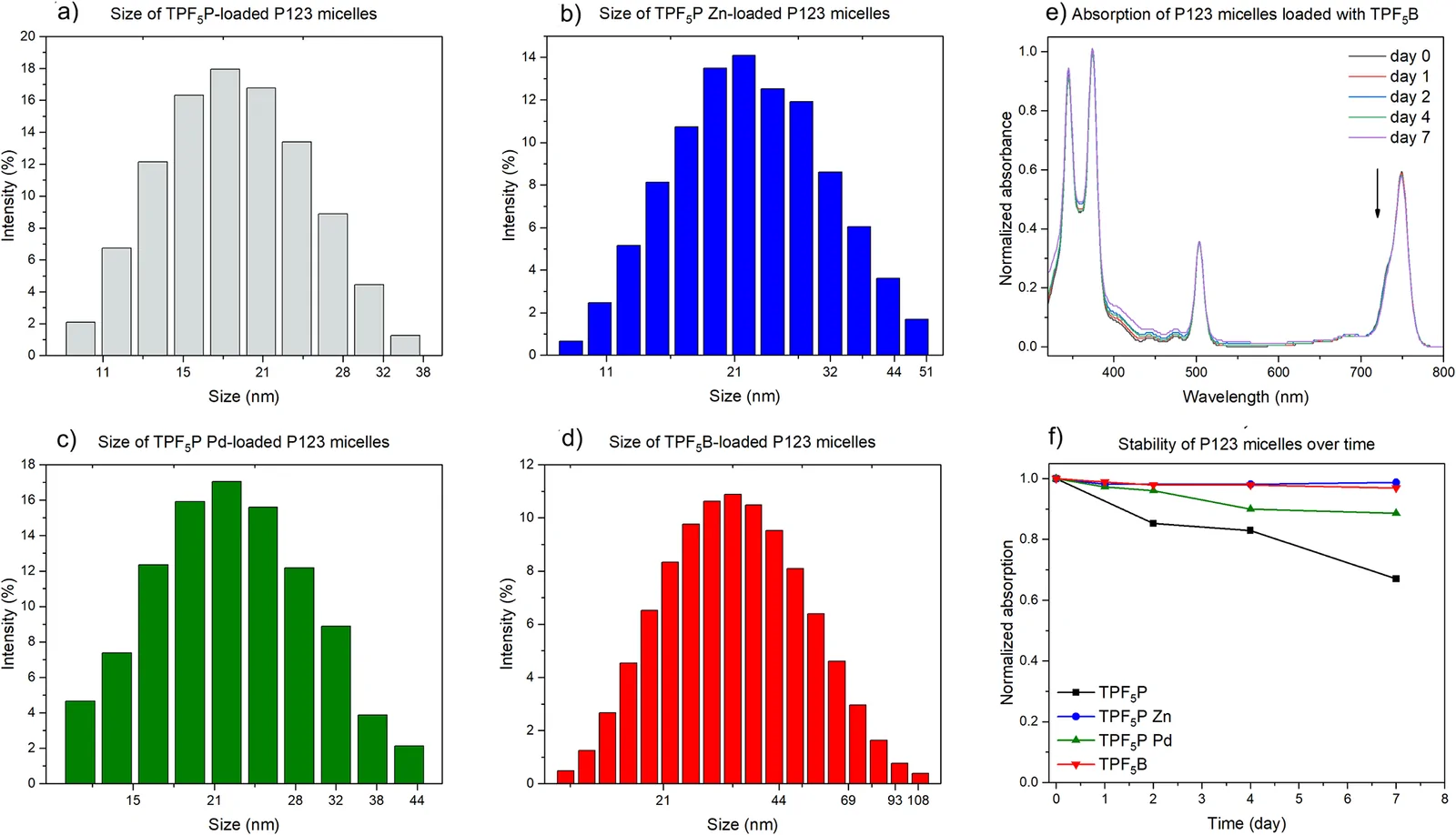
(a-d) Hydrodynamic size distributions of photosensitizers encapsulated in Pluronic P123 micelles determined by dynamic light scattering (DLS). (e) Stability of TPF_5_B-loaded micelles over time determined by absorbance measurements. (f) Stability over time for all investigated photosensitizers, assessed by monitoring the absorbance of PS-loaded P123 micelles over seven days.

The measured ζ-potentials were negative for all formulations, ranging from −8.29 to −26.83 mV (Table 1). These values should be interpreted primarily as physicochemical descriptors, not as direct predictors of colloidal stability, because Pluronic P123 is a nonionic amphiphilic polymer and stabilization is expected to arise predominantly from steric effects associated with the hydrated PEO corona, with limited contribution from electrostatic repulsion. Therefore, formulation stability was evaluated primarily by monitoring absorption changes over time. All investigated P123 formulations showed good colloidal stability under the applied conditions (Figures 1e,f, and S2). Among the studied systems, TPF_5_P showed the most pronounced decrease in absorbance over time, which may indicate partial redistribution, aggregation, or loss of effective solubilization upon prolonged storage.

### Determination of *n*-Octanol/PBS Distribution Coefficients at pH 7.4 (logD_7.4_)

The *n*-octanol/PBS distribution coefficients determined at pH 7.4 (logD_7.4_) provided additional experimental descriptors of the formulation-relevant distribution behavior of the investigated compounds (Table 1). The free-base porphyrin TPF_5_P showed the highest logD_7.4_ value (2.18), consistent with its pronounced hydrophobic character. Coordination with Zn^2+^ resulted in a lower value (logD_7.4_ = 0.35), suggesting that metal insertion alters not only the electronic structure of the macrocycle but also its apparent distribution between the aqueous and lipophilic phases.

Reduction of the porphyrin to the corresponding bacteriochlorin gave an intermediate logD_7.4_ value (1.06), indicating that macrocycle reduction modifies the balance among hydrophobic surface area, electronic structure, and environment-dependent aggregation behavior rather than simply increasing lipophilicity.^15^ This observation is also consistent with the high encapsulation efficiency of TPF_5_B, which likely reflects a favorable molecular accommodation within the Pluronic P123 micellar environment. The logD_7.4_ value for the palladium derivative could not be determined reliably because of insufficient fluorescence intensity under the assay conditions; therefore, its distribution behavior cannot be directly compared within this fluorescence-based data set.^29,30^ Calibration curves are present in Figure S3.

### Photophysical Characterization

To assess the impact of formulation on optical properties, absorption spectra were recorded in different solvents: DMSO (Figure 2a-b), aqueous buffer (1% DMSO in PBS, Figure S4), and after encapsulation in Pluronic P123 micelles in PBS buffer (Figure 2c-d). While DMSO serves as a reference solvent, micellar formulation provides a more biologically relevant environment, improving solubility and stabilizing the photosensitizers.^31–34^ The electronic absorption spectra of the investigated tetrapyrrolic derivatives (Figures 2 and S5) display the characteristic features of porphyrinoid systems, including an intense Soret band around 400 nm and Q bands in the visible to near-infrared region. Both metalation and macrocycle reduction lead to systematic spectral changes reflecting modifications in the electronic structure. Insertion of Zn^2+^ induces a bathochromic shift of the Soret band, whereas Pd^2+^ results in a hypsochromic shift, consistent with differences in metal–ligand interactions and electron density distribution within the macrocycle.^35^ Reduction of the porphyrin to bacteriochlorin leads to pronounced spectral reorganization, including hypsochromic modification and splitting of the Soret band associated with the lifting of the LUMO/LUMO+1 degeneracy, together with a marked bathochromic shift of the S_0_ → S_1_ transition, manifested by the emergence of an intense Q-band centered at ∼750 nm.^36^ These spectral changes directly affect light absorption under defined irradiation conditions and must be considered when comparing photodynamic performance across the series. Furthermore, metalation reduces the number of Q bands, from four in the free-base porphyrin (TPF_5_P) to two in the palladium complex and a single band in the zinc derivative, reflecting an increase in molecular symmetry (from D_2_h to D_4_h).^37^ This simplification of the electronic structure is expected to influence excited-state population pathways and, consequently, intersystem crossing efficiency.

**Figure 2:**
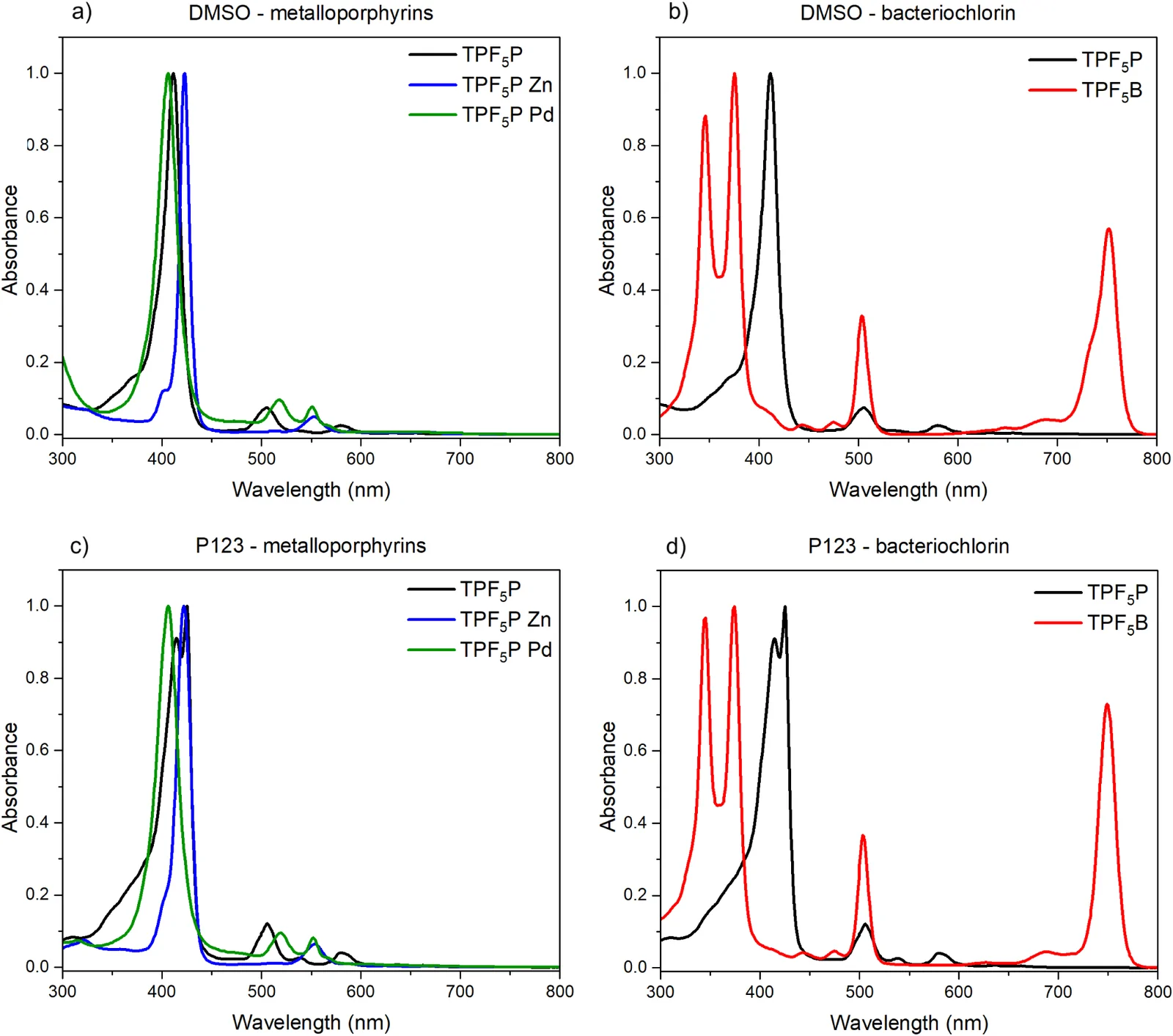
Normalized electronic absorption spectra of the investigated photosensitizers recorded in different formulations: (a,b) DMSO solution and (c,d) Pluronic P123 micelles in PBS. Panels (a,c) show the absorption spectra of porphyrin derivatives TPF_5_P, TPF_5_P Pd, TPF_5_P Zn; panels (b,d) show the absorption spectra of bacteriochlorin TPF_5_B, with the free-base porphyrin TPF_5_P included for comparison.

In general, the characteristic absorption features were largely preserved after incorporation into Pluronic P123 micelles, indicating the effective solubilization of the photosensitizers within the micellar environment. However, minor band broadening or splitting observed for selected compounds, particularly TPF_5_P, suggests formulation-dependent changes in supramolecular organization rather than complete molecular dispersion.

This behavior was more pronounced in 1% DMSO/PBS, where TPF_5_P showed clear signs of aggregation, including broadening and shifts of the Soret and Q bands (Figure S4). Nevertheless, the retention of relevant absorption bands under these conditions indicates that the systems remain suitable for a photodynamic evaluation. To compare the absorption profiles of all obtained photosensitizers, molar absorption coefficients were determined in three different formulations and are summarized in Table 2.

**Table 2:** Absorption Maxima (Abs) and Corresponding Molar Absorption Coefficients (ε) of Studied Tetrapyrroles: Porphyrins TPF_5_P, TPF_5_P Zn, TPF_5_P Pd, and Bacteriochlorin TPF_5_B, Measured in DMSO, after Encapsulation in Pluronic P123 Micelles, and 1% DMSO in PBS

	Abs/nm (ε/M^–1^ cm^–1^)					
Name	B(0,0)		*Q*_*y*_(1,0)	*Q*_*y*_(0,0)	*Q*_*x*_(1,0)	*Q*_*x*_(0,0)
**DMSO**						
**TPF**_**5**_ **P**	411 (2.4 × 10^5^)		506 (1.6 × 10^4^)	580 (5.5 × 10^3^)	632 (9.3 × 10^2^)	656 (6.5 × 10^2^)
**TPF**_**5**_ **P Zn**	422 (1.7 × 10^5^)		552 (6.9 × 10^3^)	-	-	-
**TPF**_**5**_ **P Pd**	406 (1.5 × 10^5^)		517 (1.2 × 10^4^)	550 (1.0 × 10^4^)	-	-
**TPF**_**5**_ **B**	345 (9.2 × 10^4^)	375 (9.8 × 10^4^)	-	503 (3.2 × 10^4^)	-	752 (7.5 × 10^4^)
**P123/PBS**						
**TPF**_**5**_ **P**	414 (3.7 × 10^4^)		506 (5.8 × 10^3^)	580 (2.1 × 10^3^)	622 (2.7 × 10^2^)	636 (3.5 × 10^2^)
**TPF**_**5**_ **P Zn**	422 (6.6 × 10^4^)		554 (5.9 × 10^3^)	-	-	-
**TPF**_**5**_ **P Pd**	406 (7.0 × 10^4^)		519 (8.8 × 10^3^)	552 (7.5 × 10^3^)	-	-
**TPF**_**5**_ **B**	345 (7.9 × 10^4^)	374 (8.1 × 10^4^)	-	503 (3.1 × 10^4^)	-	752 (6.1 × 10^4^)
**1% DMSO/PBS**						
**TPF**_**5**_ **P**	425 (1.9 × 10^4^)		508 (6.6 × 10^3^)	586 (2.5 × 10^3^)	-	-
**TPF**_**5**_ **P Zn**	422 (2.5 × 10^4^)		555 (3.2 × 10^3^)	-	-	-
**TPF**_**5**_ **P Pd**	410 (2.5 × 10^4^)		522 (5.7 × 10^3^)	557 (5.4 × 10^3^)	-	-
**TPF**_**5**_ **B**	345 (3.1 × 10^4^)	375 (3.1 × 10^4^)	-	506 (1.8 × 10^4^)	-	747 (2.0 × 10^4^)

### Fluorescence Quantum Yield

For further characterization, fluorescence spectra were recorded for all synthesized compounds in different solvents or formulations, and the fluorescence quantum yields (Φ_F_) were determined. Fluorescence was observed for three of the four compounds and systematically decreased upon metal coordination (Figure S5). The free-base porphyrin (TPF_5_P) exhibited the highest fluorescence quantum yield, which decreased by approximately half in the zinc derivative (TPF_5_P Zn) and was completely quenched in the palladium complex (TPF_5_P Pd). This trend reflects the increasing strength of SOC, where the heavy-atom effect enhances ISC and promotes efficient population of the triplet excited state at the expense of radiative decay. The complete quenching of fluorescence in TPF_5_P Pd therefore indicates highly efficient singlet-to-triplet conversion, although photodynamic performance ultimately depends on the balance among triplet-state population, triplet lifetime, and the dominant ROS-generating pathways. However, the absence of fluorescence limits the applicability of fluorescence-based analytical and imaging techniques, precluding direct tracking of the compound without additional labeling.^38^ In contrast, the retained fluorescence of TPF_5_B supports its potential use in imaging-guided PDT.

Table 3 summarizes the key photophysical and physicochemical parameters of the investigated photosensitizers, including absorption and emission maxima, molar absorption coefficients, fluorescence, singlet oxygen, and photodecomposition quantum yields.

**Table 3:** Summary of Photochemical and Physicochemical Properties of Fluorinated Tetrapyrrolic Compounds: Absorption and Fluorescence Maxima (λ_max_), Molar Absorption Coefficients (ε_max_) for the Most Significant Bands, Quantum Yields of Fluorescence (Φ_F_), Singlet Oxygen Quantum Yield (Φ_Δ_), Photodecomposition Quantum Yield (Φ_PD_) Determined in Two Solvents: EtOH and DMSO, and Two Formulations: After Encapsulation in Pluronic P123 Micelles and in 1% DMSO in PBS Solution

	Absorption		Emission	Quantum yields				
Name	λ_max_ [nm]	ε_max_ [M^–1^ cm^–1^]	λ_max_ [nm]	Φ_F_ (EtOH)	Φ_Δ_ (EtOH)	Φ_PD_ (DMSO)	Φ_PD_ (P123)	Φ_PD_ (1% DMSO/PBS)
**TPF**_**5**_ **P**	411	2.4 × 10^5^	702	0.0644	0.84	1.0 × 10^–5^	3.7 × 10^–6^	3.4 × 10^–5^
**TPF**_**5**_ **P Zn**	422	1.7 × 10^5^	648	0.0356	0.95	1.8 × 10^–5^	4.8 × 10^–6^	3.0 × 10^–5^
**TPF**_**5**_ **P Pd**	406	1.5 × 10^5^	-	-	0.77	3.1 × 10^–6^	9.1 × 10^–7^	4.0 × 10^–5^
**TPF**_**5**_ **B**	752	7.5 × 10^4^	756	0.0998	0.36	1.2 × 10^–5^	4.0 × 10^–6^	5.0 × 10^–5^

### Quantum Yield of Photodecomposition

Stability under irradiation can be evaluated by measurements of photodecomposition quantum yield (Φ_PD_). The value for each photosensitizer was determined by measuring electronic absorption spectra after irradiation at 420 ± 20 nm for porphyrins, and at 750 ± 5 nm for bacteriochlorins. Φ_PD_ was calculated for PS dissolved in organic solvent (DMSO), after encapsulation in Pluronic P123 micelles, and in a solution of PBS with 1% DMSO to mimic the conditions of *in vitro* PS administration. As summarized in Table 3, incorporation into Pluronic P123 micelles resulted in a marked decrease in the quantum yields of photodecomposition for all investigated photosensitizers, reaching approximately 1 order of magnitude for most compounds. This effect was accompanied by lower apparent molar absorption coefficients relative to DMSO, which may reflect changes in the local environment and optical response of the micellar formulations. The observed reduction in photodecomposition (Figure S6) cannot be attributed solely to reduced photon absorption but also suggests a protective effect of the micellar environment, which may limit direct exposure of the photosensitizer to reactive intermediates and modulate oxygen accessibility. This stabilization highlights the role of appropriate delivery vehicles in tuning photochemical behavior under biologically relevant conditions.

Within the same formulation, the bacteriochlorin derivative exhibits consistently higher photodecomposition rates than those of the corresponding porphyrin analogues. This behavior can be rationalized by the higher HOMO energy levels typically associated with bacteriochlorins, which may increase the susceptibility to oxidative processes and facilitate photoinduced degradation pathways in the presence of oxygen.^5,39^ Comparison of metalated derivatives reveals distinct formulation-dependent trends. In DMSO and micellar systems, the Zn­(II) complex undergoes more pronounced photodecomposition, whereas the Pd­(II) derivative exhibits higher resistance, with quantum yields lower by approximately 1 order of magnitude. In contrast, under diluted aqueous conditions (1% DMSO in PBS), photodecomposition rates become more comparable across the series.^40,41^ These differences indicate a strong interplay between the electronic structure and microenvironment in determining photostability.

Photodecomposition quantum yield is an important parameter in photosensitizer design, and its optimal range depends on the therapeutic context. In V-PDT, partial photobleaching may help limit prolonged photosensitizer activity after irradiation, whereas in C-PDT, enhanced photostability is desirable to sustain photodynamic activity over extended drug-to-light intervals. Fluorination contributes to increased resistance to photobleaching, consistent with the electron-withdrawing character of fluorine substituents, which can stabilize the macrocycle and reduce its susceptibility to oxidative degradation.^42,43^ The studied bacteriochlorin derivative demonstrates a favorable balance between photoreactivity and photostability, which may support sustained photodynamic activity after tumor accumulation *in vivo*.^44^ This interplay appears to be critical for efficient photodynamic performance.

### Transient Absorption and Triplet-State Dynamics

Transient absorption spectra were recorded using a laser flash photolysis spectrometer to further characterize the photophysical properties of the synthesized photosensitizers and establish the influence of encapsulation. Figure 3a-c presents the ground-state absorption, fluorescence, and transient absorption (ΔOD) spectra of the investigated porphyrin photosensitizers, while Figure 3d shows the corresponding data for the bacteriochlorin. The positive ΔOD signals are consistent with the population of the triplet excited state and reveal distinct triplet–triplet absorption bands appearing at longer wavelengths than the ground-state Soret band. Interestingly, TPF_5_P Pd exhibits a weak emission feature in the 650–700 nm range, observed as a small negative ΔOD signal. This emission is not detectable by steady-state fluorometry, indicating an extremely low fluorescence quantum yield and highly efficient ISC.

**Figure 3:**
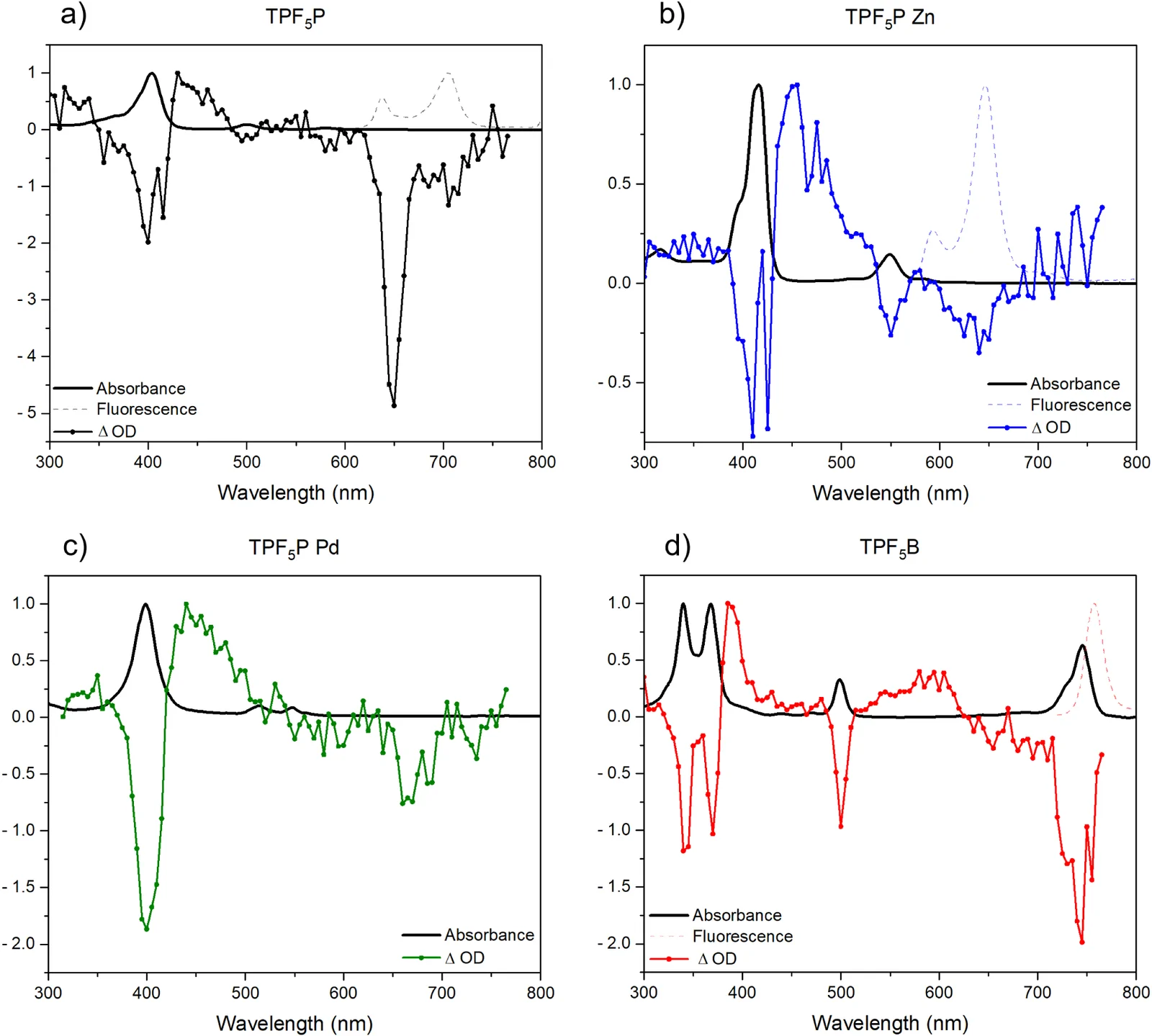
Electronic absorption (solid lines), fluorescence (dashed lines), and transient absorption at 1.5–2.5 ns (ΔOD, lines with symbols) spectra of the synthesized porphyrins (a-c) and bacteriochlorin (d) measured in ethanol.

Triplet-state lifetimes were determined under both ambient and deoxygenated conditions in three different media (Table 4). Removal of oxygen prolonged triplet-state lifetimes from the nanosecond range under air-equilibrated conditions to the microsecond range under argon-saturated conditions for all investigated compounds, confirming efficient quenching of the triplet state by molecular oxygen. The magnitude of this effect was structure- and medium-dependent, being particularly pronounced in molecular solvents and less marked after encapsulation in Pluronic P123 micelles. All decay curves are provided in Figures S7 and S8. Notably, under argon-saturated conditions, the bacteriochlorin derivative does not follow the trend expected from its short triplet lifetime under air-equilibrated conditions. Instead, its lifetime becomes longer than that of the Pd­(II) complex in all investigated media. Although minor differences in the efficiency of oxygen removal cannot be excluded, this observation further supports the efficient oxygen-mediated quenching of the bacteriochlorin triplet state under ambient conditions.

**Table 4:** Triplet-State Lifetimes (τ_T_) of Porphyrins and Bacteriochlorin Measured in Ethanol, DMSO, and Pluronic P123 Micelles in PBS under Air-Equilibrated and Argon-Saturated Conditions^a^

	Air [ns]				Ar [μs]	
Name	DMSO	EtOH	P123	DMSO	EtOH	P123
**TPF**_**5**_ **P**	1353	480	1950	219	950	19
**TPF**_**5**_ **P Zn**	2535	538	2229	224	498	43
**TPF**_**5**_ **P Pd**	1007	343	1339	101	4	18
**TPF**_**5**_ **B**	857	170	891	175	162	27

a Values obtained under ambient atmosphere are reported in nanoseconds (ns), whereas values measured after argon saturation are reported in microseconds (μs), reflecting the substantial lifetime prolongation upon oxygen removal.

Clear structure-dependent trends are observed. The free-base porphyrin TPF_5_P exhibits an intermediate lifetime, reflecting the absence of metal-induced spin–orbit coupling. Among metalloporphyrins, the palladium complex displays much shorter triplet lifetimes than the zinc analogue, consistent with stronger heavy-atom effect and accelerated triplet-state deactivation. Thus, although Pd­(II) enhances triplet-state quantum yield, it simultaneously accelerates triplet-state decay. The observed photophysical behavior of Pd­(II) complexes is particularly relevant in light of clinically evaluated palladium-based photosensitizers such as padeliporfin (TOOKAD Soluble).^14^ In contrast, Zn­(II) complexes provide a more favorable balance between efficient intersystem crossing and sufficiently long-lived triplet states, which is advantageous for energy transfer to molecular oxygen and singlet oxygen generation.^45,46^

The bacteriochlorin derivative exhibits shorter triplet-state lifetimes than those of porphyrins under air-equilibrated conditions, reflecting a shift in the dominant deactivation pathways. In this system, increased charge-transfer character may promote more efficient triplet-state quenching, whereas porphyrins predominantly undergo energy transfer processes associated with longer-lived triplet states. This mechanistic divergence highlights the role of macrocycle electronic structure in directing excited-state deactivation pathways.^8^ Consequently, the triplet-state lifetime emerges as a key parameter governing photodynamic performance, and strong heavy-atom effects may limit the suitability of Pd­(II) in bacteriochlorin-based systems. Such differences in triplet-state behavior are expected to influence the balance between energy- and electron-transfer processes and, consequently, the mechanisms of ROS generation.

### Reactive Oxygen Species Generation

Building on these photophysical differences, the ability of the investigated photosensitizers to generate ROS was evaluated by using complementary fluorescence probes, direct detection of singlet oxygen phosphorescence, and EPR (electron paramagnetic resonance) spectroscopy.

Ethanol was used for comparative singlet oxygen quantum-yield determination because it provides a homogeneous solvent environment and allows measurements against photosensitizer standards with established Φ_Δ_ values. In contrast, 1% DMSO in PBS and Pluronic P123 micelles were used as biologically relevant media to compare ROS generation under conditions closer to those applied in the cellular experiments. The latter measurements were therefore interpreted as formulation-matched comparative ROS readouts rather than absolute quantum yield determination.

Singlet oxygen generation was assessed using both indirect DMA (dimethylanthracene)-based detection and direct phosphorescence monitoring at 1270 nm (Figure 4a,d and Figures S9–S11). Direct detection of ^1^O_2_ phosphorescence revealed trends consistent with those obtained from DMA fluorescence decay, with TPF_5_P Zn identified as the most efficient singlet oxygen-generating porphyrin. TPF_5_P Zn showed the highest Φ_Δ_ values (Table 3), indicating a dominant Type II mechanism associated with efficient energy transfer to molecular oxygen.

**Figure 4:**
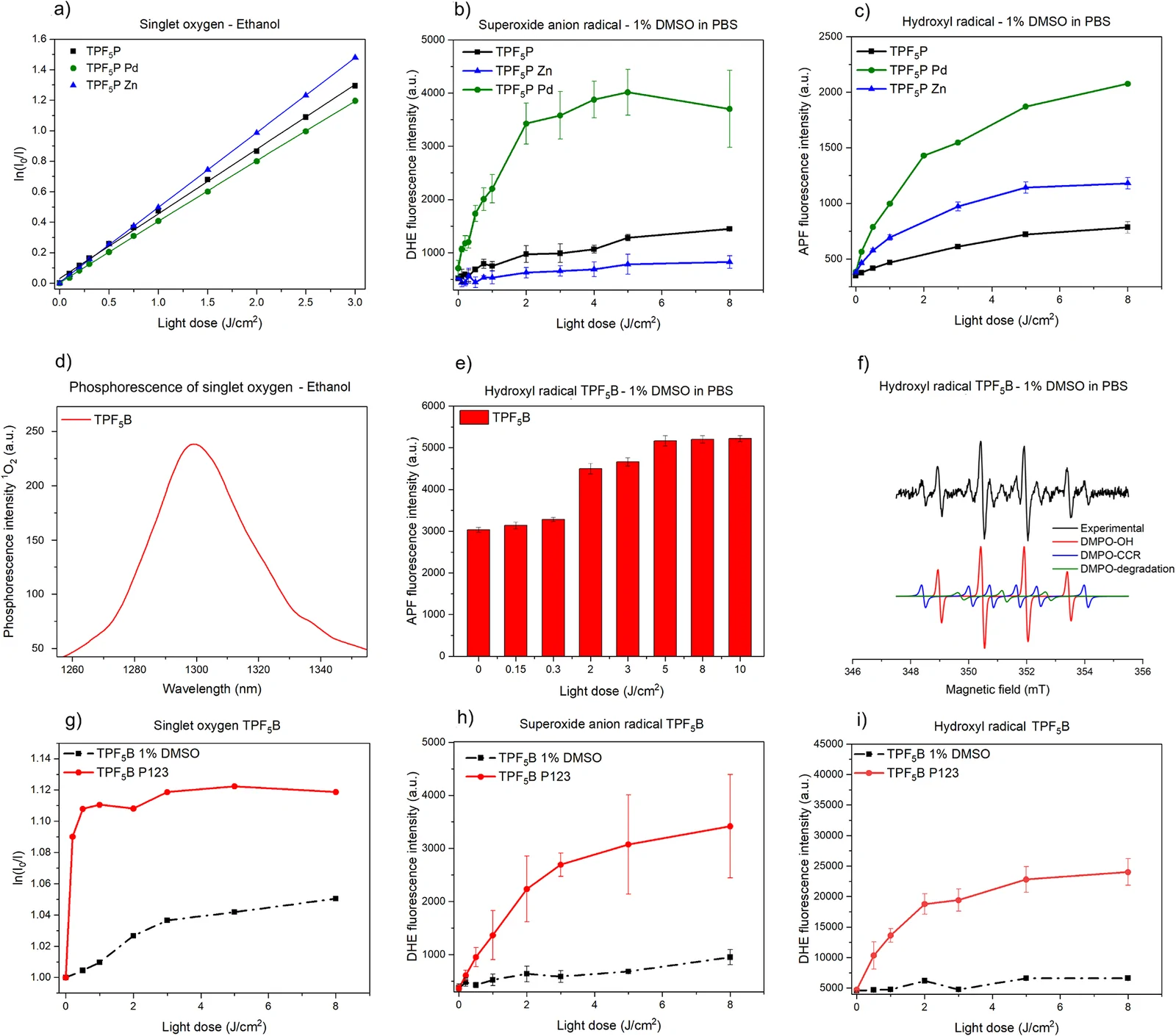
(a) Corresponding ln­(*I*_0_/*I*) plots derived from the decay of DMA fluorescence intensity for porphyrins upon irradiation with blue light (420 ± 20 nm). (b) Superoxide anion radical generation by porphyrins assessed using the DHE probe under identical irradiation conditions. (c) Hydroxyl radical generation by porphyrins assessed using the APF probe under identical irradiation conditions. (d) Phosphorescence spectra of singlet oxygen recorded in ethanol for TPF_5_B. (e) Hydroxyl radical generation by TPF_5_B bacteriochlorin measured with APF upon NIR light irradiation (750 ± 5 nm). (f) EPR spectrum of the DMPO–OH adduct recorded after irradiation of an aqueous TPF_5_B solution (1% DMSO in PBS) with NIR light (750 ± 5 nm), shown together with simulated contributions from DMPO–OH, DMPO–CCR, and characteristic DMPO degradation products (irradiation time ∼20 min). (g-i) ROS generation profile of TPF_5_B in 1% DMSO/PBS and in P123 formulation: (g) singlet oxygen-sensitive DMA readout, (h) superoxide-related DHE readout, and (i) hydroxyl radical/hROS-related APF readout. Photosensitizer concentration was 5 μM, DMA (1% DMSO in PBS), DHE, and APF concentration was 10 μM, and DMA (ethanol, panel a) concentration was 2 μM. Direct quantitative comparison between porphyrins and bacteriochlorin should be made with caution because different excitation wavelengths were used (420 ± 20 nm for porphyrins and 750 ± 5 nm for bacteriochlorin).

To complement these measurements, ROS generation was further compared under formulation-matched conditions by using 1% DMSO in PBS and Pluronic P123 micelles. These experiments were performed by using the same photosensitizer concentration, irradiation wavelengths, light sources, and irradiation times as those applied in the biological assays. The results were not treated as absolute quantum yield determinations because indirect probe-based calculations in micellar systems may be affected by probe partitioning, local concentration effects, and environment-dependent fluorescence responses. Instead, these experiments provide a biologically relevant comparative readout of ROS formation under the conditions used for *in vitro* photodynamic studies.

Superoxide-related ROS formation was evaluated using the DHE (dihydroethidium) probe (Figures 4b and S12). A clear trend was observed, with TPF_5_P Pd showing the highest DHE-associated response, consistent with efficient ISC induced by the heavy atom effect and an increased contribution of electron-transfer pathways. Notably, the free-base porphyrin exhibited a stronger DHE response than the zinc derivative, which aligns with the dominant type II character of TPF_5_P Zn. These observations indicate that Pd­(II) coordination favors radical-generating pathways, whereas the zinc complex is more strongly associated with energy-transfer processes, leading to singlet oxygen formation.

Hydroxyl radical generation, assessed using the APF (aminophenyl fluorescein) probe (Figure 4c,e) and supported by EPR spectroscopy (Figure 4f), followed a similar trend. The Pd­(II) porphyrin displayed the highest apparent activity among the porphyrin series, while the bacteriochlorin derivative (TPF_5_B) also showed a clear hydroxyl radical generation under NIR excitation. The corresponding EPR spectra provided direct evidence for the formation of DMPO–OH adducts, as well as carbon-centered radical species, confirming the involvement of radical-mediated processes.^47,48^ Additionally, the influence of DMSO, a known hydroxyl radical scavenger,^49^ was evaluated in the range of 0.1–3.0% in PBS. Under the experimental conditions used in this study, 1% DMSO does not affect the APF-based ROS readout (Figure S13).

The bacteriochlorin derivative TPF_5_B exhibited a distinct ROS profile (Figure 4d-f), characterized by a relatively low singlet oxygen quantum yield (Table 3) but efficient radical-associated ROS generation. This behavior can be attributed to stronger charge-transfer interactions with molecular oxygen, leading to faster triplet-state quenching and favoring electron-transfer over energy-transfer pathways. As a result, the reduced Φ_Δ_ value of TPF_5_B should not be interpreted as diminished photodynamic potential but as evidence of a mechanistic shift toward Type I photochemistry. This feature is particularly advantageous under hypoxic conditions, where oxygen availability is limited and radical-mediated pathways may contribute substantially to photodynamic efficacy.^50–53^

Comparison of ROS generation in 1% DMSO/PBS and in Pluronic P123 micelles revealed enhanced probe responses after micellar formulation for all investigated photosensitizers, as illustrated for TPF_5_B in Figure 4g-i and for the remaining compounds in Figure S12. This effect was observed for DMA-, DHE-, and APF-based readouts, indicating improved overall photoreactivity under formulation-matched irradiation conditions. The observed enhancement is likely associated with improved solubilization, reduced aggregation, and a more favorable local microenvironment, leading to more efficient interaction of the excited photosensitizer with molecular oxygen. These data support the use of Pluronic P123 micelles as a formulation strategy that improves biologically relevant ROS generation without requiring potentially misleading conversion of probe responses in micelles into absolute quantum yields.

### *In Vitro* Studies

Following physicochemical characterization, the biological performance of the investigated photosensitizers was evaluated *in vitro*, including cellular uptake, intracellular localization, dark cytotoxicity, and photodynamic activity. Experiments were conducted by using B16–F10 murine melanoma and CT26 murine colon carcinoma cells, which are well-established tumor models in preclinical PDT studies. To assess the suitability of the compounds in V-PDT, their activity was additionally evaluated in 2H-11 murine endothelial cells.

For C-PDT, efficient intracellular accumulation of the photosensitizer is essential to enable localized ROS generation upon illumination and subsequent damage to intracellular components. Given the hydrophobic character of the investigated compounds, Pluronic P123 polymeric micelles were evaluated as delivery vehicles to improve aqueous dispersion and cellular delivery. This formulation is known to undergo cellular internalization through endocytic pathways and have previously been shown to improve the uptake and photodynamic activity of tetrapyrrolic photosensitizers.^16,54^ Accordingly, cellular uptake was compared for photosensitizers administered in 1% DMSO-containing medium and after encapsulation in P123 micelles (Figure 5). This approach allows a direct assessment of the formulation effect on cellular accumulation while preserving the photophysical properties required for ROS generation under biologically relevant conditions. However, the extent to which *in vitro* conditions reflect *in vivo* behavior remains inherently limited.

**Figure 5:**
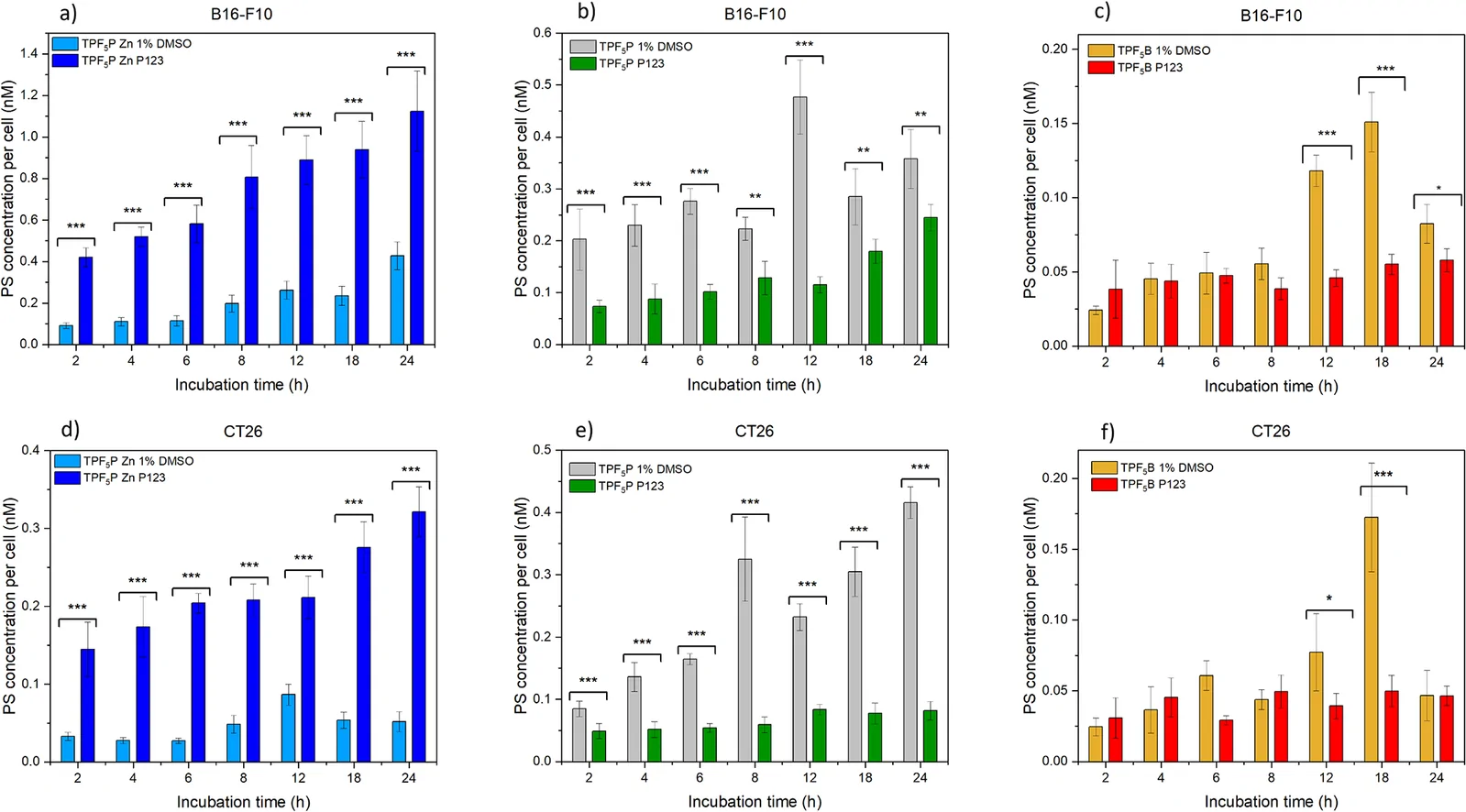
Time-dependent cellular uptake of porphyrins: TPF_5_P, TPF_5_P Zn, and bacteriochlorin TPF_5_B into B16–F10 (a-c) and CT26 (d-f) cells. Formulations were prepared in PBS with 1% DMSO or encapsulated in P123 micelles. The concentration in cancer cells was evaluated over 24 h based on fluorescence intensity divided by the number of cells seeded per well. Data are presented as mean ± SD (*n* = 4). Statistical significance was assessed between the PS concentration in the two formulations. *p < 0.05, **p < 0.01, ***p < 0.001.

### Cellular Uptake

The internalization of PS into cancer cells was evaluated by measuring their fluorescence signal in cell lysates after different times of incubation (2–24 h). The cellular accumulation of the investigated photosensitizers was strongly influenced by their formulation and lipophilicity. As indicated by the experimentally determined logD_7.4_ values (Table 1), TPF_5_P Zn is the least lipophilic compound in the series, and its intracellular uptake was markedly enhanced upon encapsulation in Pluronic P123 micelles, consistent with improved delivery through endocytic pathways (Figure 5a,d). In contrast, the more lipophilic free-base porphyrin TPF_5_P (logD_7.4_ = 2.18) showed only limited improvement in cellular uptake after P123 encapsulation (Figure 5b,e). This behavior may reflect a combination of stronger aggregation tendency, less favorable redistribution from the micellar environment, and reduced effective interaction with the cell membrane, as also suggested by absorption spectral changes observed in an aqueous medium (Figure S4). The uptake differences between TPF_5_P and TPF_5_P Zn should not be attributed to lipophilicity alone but rather to the combined effects of apparent distribution properties, supramolecular organization, encapsulation behavior, and cellular delivery.

The bacteriochlorin derivative TPF_5_B (logD_7.4_ = 1.06) exhibited intermediate behavior (Figure 5c,f). Comparable levels of cellular accumulation were observed for both formulations during the initial incubation period. However, after 12 h, the 1% DMSO/PBS formulation showed an increased intracellular accumulation, followed by a gradual decline over time. This decrease likely reflects dynamic cellular processes such as proliferation, efflux, intracellular redistribution, or metabolic degradation.^55^ Together, these observations highlight the importance of balancing apparent lipophilicity and formulation to achieve efficient cellular delivery, which is a critical determinant of photodynamic efficacy. Calibration curves for lysis-based fluorescence measurements are shown in Figure S14 and were used to determine the concentration of photosensitizers in cell lysates by correlating measured fluorescence intensities with the corresponding concentrations. The concentration was normalized to the number of cells seeded per well.

Beyond total cellular uptake, intracellular localization is an important determinant of PDT outcome, because the short lifetime and limited diffusion range of ROS restrict photodamage to sites close to photosensitizer accumulation. In particular, endoplasmic reticulum (ER) localization is relevant because ROS-mediated ER stress can activate apoptosis through the unfolded protein response (UPR) and Ca^2+^ signaling pathways.^56,57^ Other organelles, including mitochondria, the Golgi apparatus, and lysosomes, can also contribute to the photodynamic response depending on the site of ROS generation. ER stress may additionally promote immunogenic cell death (ICD), a process associated with the activation of antitumor immune responses.^58^

Intracellular localization was therefore examined for the lead compound TPF_5_B, in CT26 cells after administration either in 1% DMSO-containing medium or after encapsulation in Pluronic P123 micelles (Figures 6, S15 and S16), by fluorescence confocal microscopy. Colocalization analysis based on Pearson correlation coefficients revealed formulation-dependent differences in the intracellular distribution of TPF_5_B.

**Figure 6:**
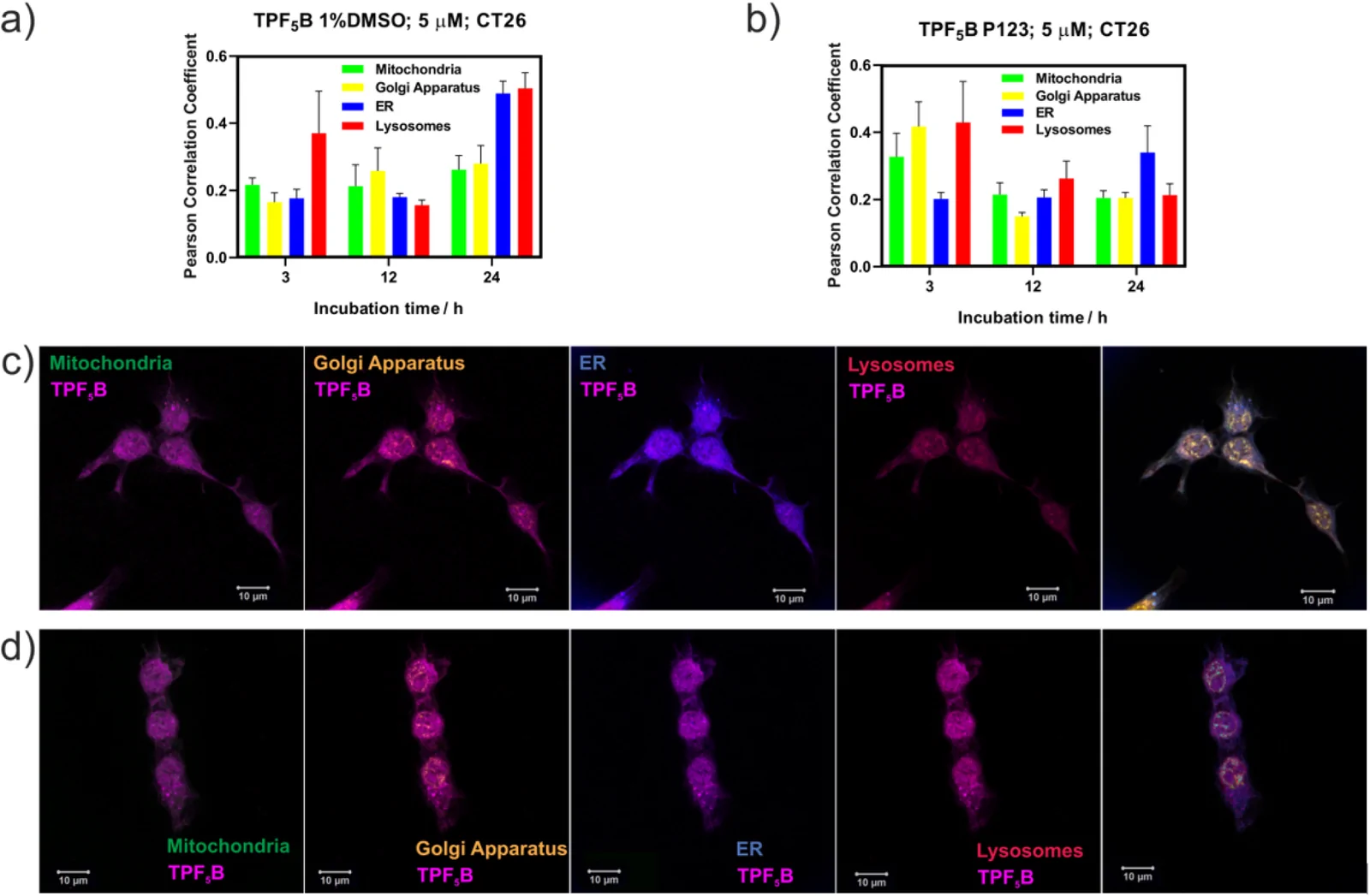
(a) Time-dependent colocalization of TPF_5_B, either administered in DMSO (final concentration 1%) or (b) loaded into Pluronic P123 micelles, with mitochondria (MTCO1), Golgi apparatus (GM130), endoplasmic reticulum (DiOC_6_(3)), and lysosomes (LAMP-1). Colocalization was assessed using Pearson correlation coefficients based on at least seven images per data point after background fluorescence subtraction with the JACoP plugin in ImageJ.^59^ (c) Representative confocal fluorescence microscopy images of CT26 cells following 24 h exposure to TPF_5_B in 1% DMSO. (d) Representative confocal fluorescence microscopy images of CT26 cells after 24 h treatment with TPF_5_B encapsulated in P123 micelles. Results are expressed as mean ± SD (*n* ≥ 7).

After 24 h of incubation, TPF_5_B administered in DMSO stock (final DMSO concentration in the cell culture media: 1%) showed pronounced colocalization with the ER and lysosomes (Figure 6a,c). In contrast, TPF_5_B delivered in Pluronic P123 micelles displayed the highest colocalization with the ER, whereas colocalization with mitochondria, the Golgi apparatus, and lysosomes remained at comparable but lower levels (Figure 6b,d). The relatively high lysosomal colocalization observed for the DMSO formulation may indicate lysosomal sequestration or involvement of endocytic/autophagy-related trafficking pathways. High lysosomal colocalization at the early 3 h time point further supports the contribution of endocytic uptake and intracellular trafficking to the TPF_5_B distribution. By comparison, the micellar formulation showed a more balanced distribution across intracellular structures at the earliest time point followed by progressive enrichment in the ER. This formulation-dependent ER accumulation is particularly relevant for PDT because ER-associated photodamage is known to efficiently trigger oxidative stress-mediated cell death pathways.

### Dark Cytotoxicity

Cell viability, assessed using the Alamar Blue assay, remained high across all tested conditions, indicating that the compounds are well-tolerated in the absence of light. As shown in Figure 7, all synthesized photosensitizers exhibited minimal dark cytotoxicity toward B16–F10 (Figure 7a) and CT26 (Figure 7b) cells after incubation for 24 h. This low intrinsic cytotoxicity fulfills a key requirement for photosensitizers intended for PDT.

**Figure 7:**
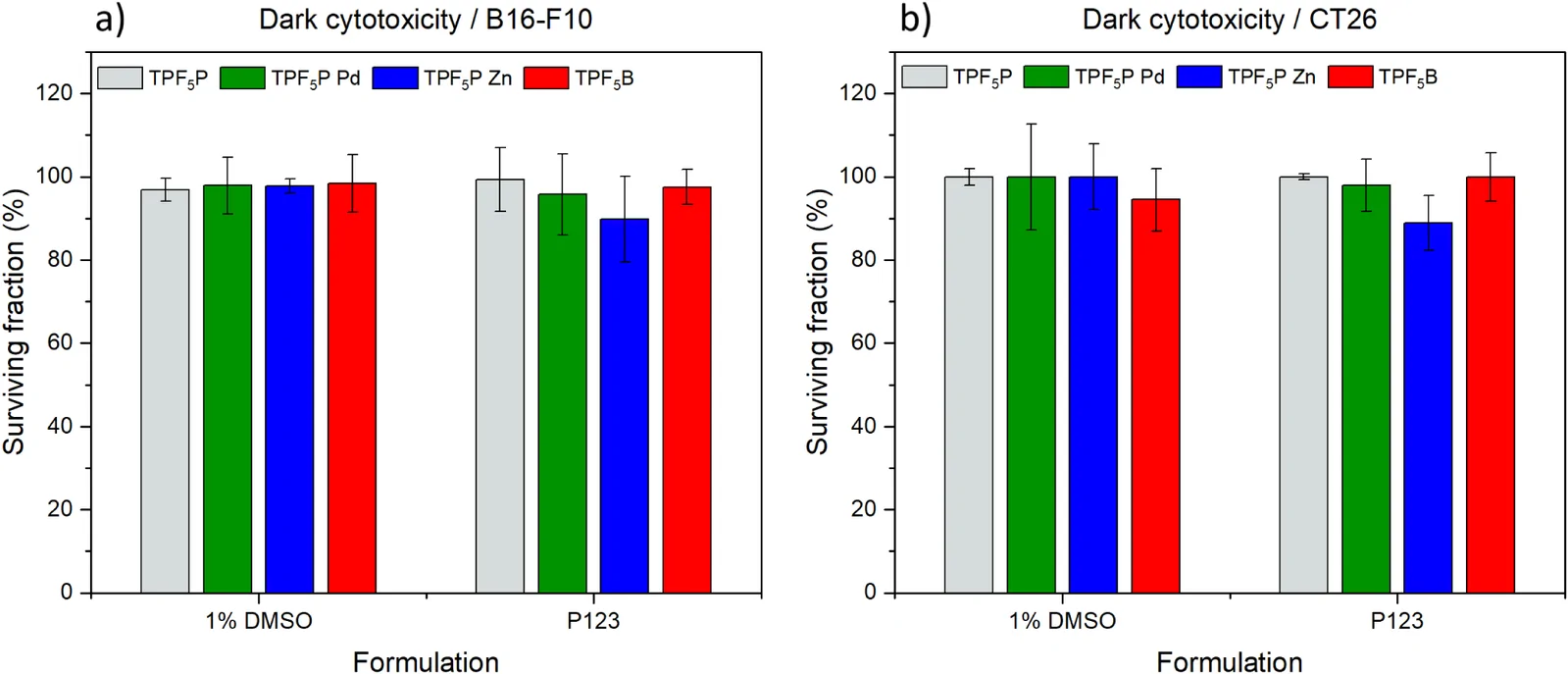
Dark cytotoxicity of porphyrins and bacteriochlorin in: (a) B16–F10 and (b) CT26 cells after 24 h incubation without irradiation. Photosensitizers were administered either in medium containing 1% DMSO or after encapsulation in Pluronic P123 micelles at 20 μM. Data are presented as mean ± SD. No statistically significant changes were observed.

### Intracellular ROS Generation

The effectiveness of ROS generation in cells was evaluated by green fluorescence measurements of fluorescein formed after APF reaction with highly reactive oxygen species, including hydroxyl radical, after cell internalization of PS, APF, and irradiation with an appropriate wavelength. Encapsulation was found to influence intracellular ROS production. As demonstrated in Figure 8a-d, hydroxyl radical generation is consistently higher for photosensitizers formulated in Pluronic P123 micelles compared to those in 1% DMSO across both tested cell lines, which is consistent with previous findings on ROS generation (Figure 4). This effect can be attributed to reduced aggregation of hydrophobic photosensitizers upon P12 encapsulation. In aqueous environments, non-encapsulated compounds are prone to aggregation driven by hydrophobic interactions and π–π stacking, which leads to self-quenching and diminished photochemical activity.^60–62^ Micellar encapsulation improves photosensitizer dispersion and limits aggregation, thereby enabling more efficient ROS generation.^63^ This is consistent with the preservation of excited-state properties in micellar environments.

**Figure 8:**
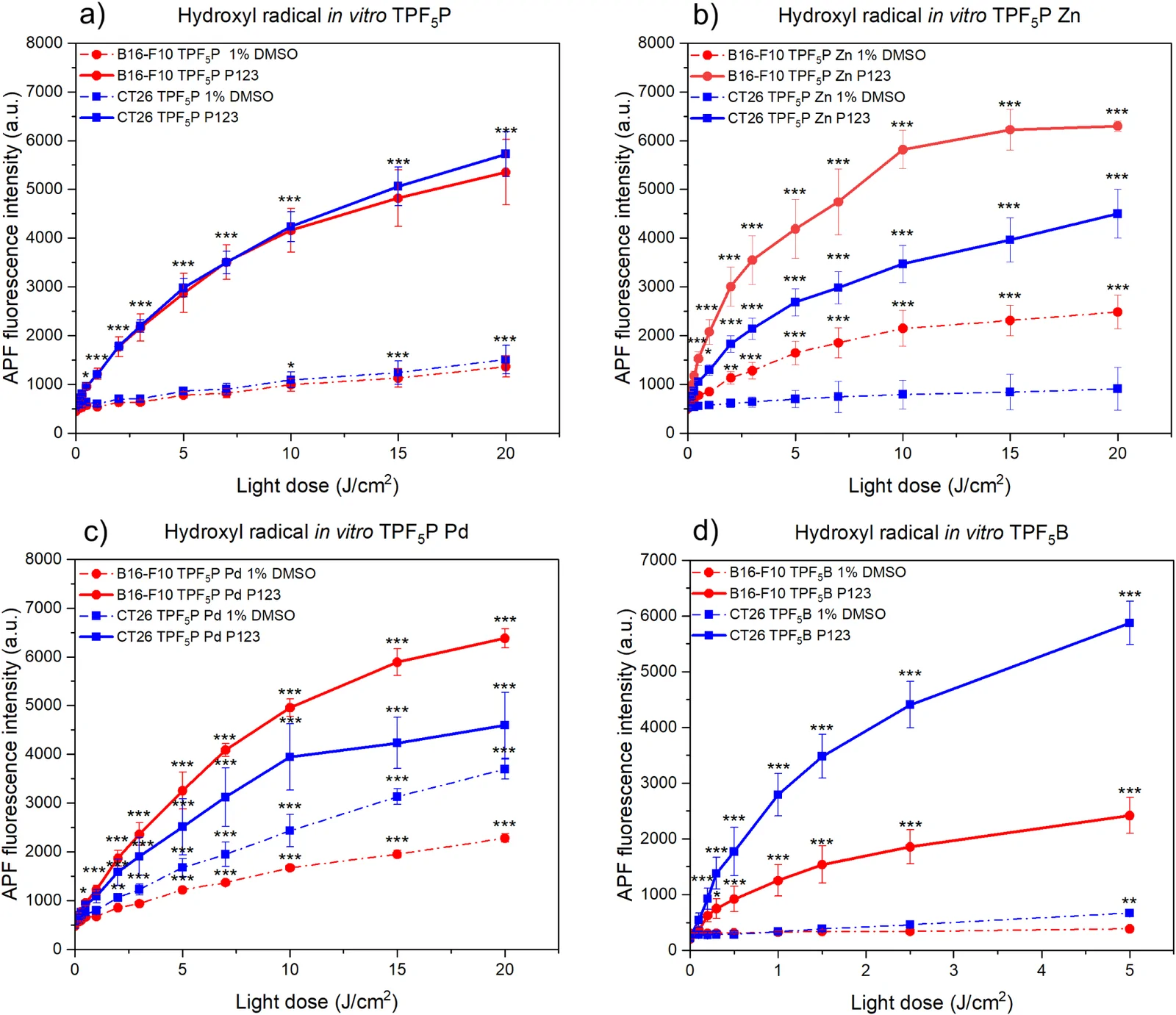
APF-derived green fluorescence intensity in B16–F10 and CT26 cells after irradiation in the presence of porphyrins 10 μM (a-c) with blue light (420 ± 20 nm) and bacteriochlorin (d) with NIR light (750 ± 5 nm) in DMSO, with final concentration of 1% in culture medium or encapsulated in Pluronic P123 micelles. Data are presented as mean ± SD (*n* = 4). *p < 0.05, **p < 0.01, ***p < 0.001.

### Photodynamic Effect

The photodynamic activity of the investigated photosensitizers was evaluated in B16–F10, CT26, and 2H-11 cells (Figures 9 and 10), integrating their photophysical properties, cellular uptake, and ROS generation into a single functional outcome. The metabolic activity (a marker of cell viability) 24 h postirradiation was determined by AlamarBlue assay.

**Figure 9:**
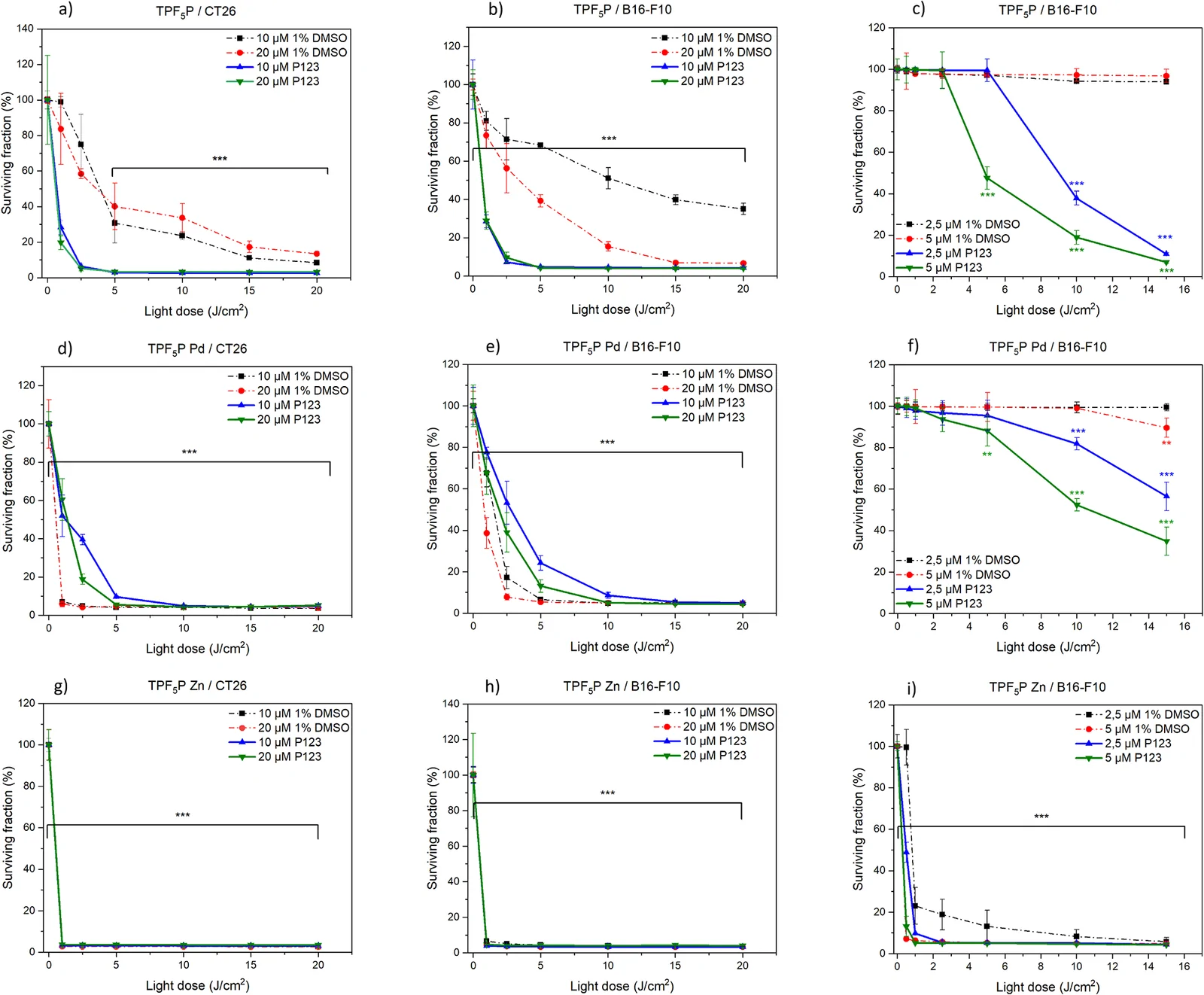
Photodynamic effect of porphyrins: (a-c) TPF_5_P, (d-f) TPF_5_P Pd, (g-i) TPF_5_P Zn, against CT26 and B16–F10 cells, in different formulations (DMSO, with a final concentration of 1% in culture medium, and P123 micelles) at concentrations ranging from 20 to 2.5 μM. Cells treated with the photosensitizer were irradiated with blue light (420 ± 20 nm). Data are presented as mean ± SD (*n* = 4). *p < 0.05, **p < 0.01, ***p < 0.001.

**Figure 10:**
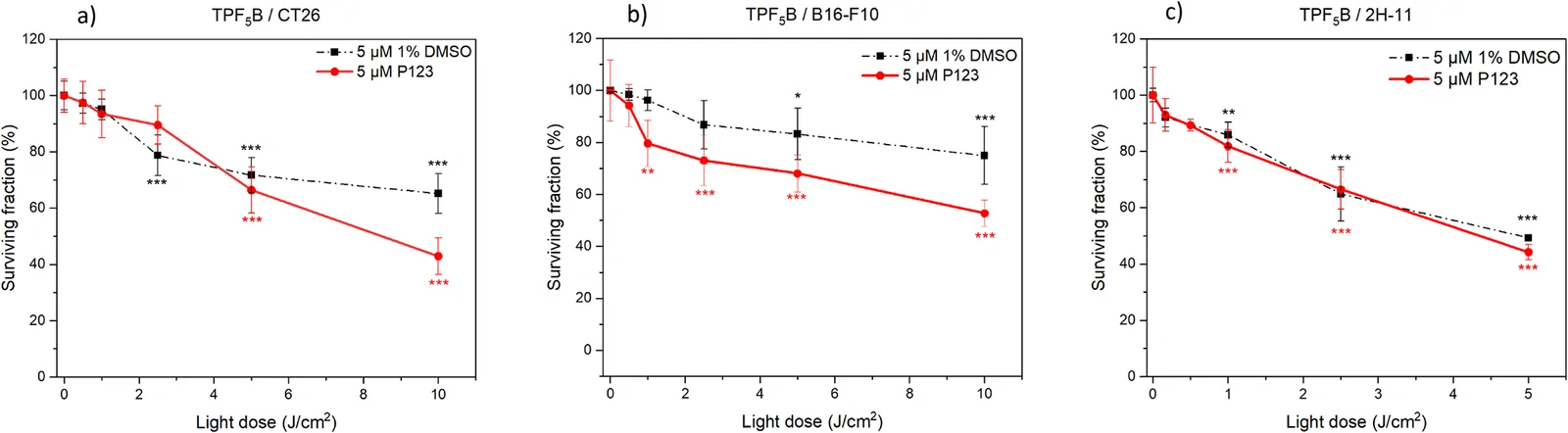
Photodynamic effect of bacteriochlorin: TPF_5_B against (a) CT26, (b) B16–F10, and (c) 2H-11 cells, in different formulations (DMSO, with a final concentration of 1% in culture medium, and P123 micelles) at a concentration of 5 μM. Cells treated with the photosensitizer were irradiated with NIR light (750 ± 5 nm). Data are presented as mean ± SD (*n* = 4). *p < 0.05, **p < 0.01, ***p < 0.001.

Porphyrin derivatives exhibit a clear and concentration-dependent photodynamic response (Figure 9a-i). Encapsulation in P123 micelles significantly enhances their photodynamic activity, particularly at lower concentrations. For TPF_5_P, micellar formulation enables near-complete loss of cell viability at 10–20 μM after short irradiation times, whereas the non-encapsulated compound shows only partial cytotoxicity under identical conditions. At 2.5–5 μM, photodynamic activity is observed only for the micellar delivery, highlighting the critical role of formulation in determining biological performance.

Metalloporphyrins display enhanced photodynamic efficacy compared to the free-base porphyrin, consistent with increased ISC and ROS generation (Figure 4). Among them, the zinc derivative (TPF_5_P Zn) shows the highest potency, inducing near-complete loss of cell viability after short irradiation times at 10–20 μM. Even at 2.5 μM, substantial cytotoxicity is observed, although for the 1% DMSO/PBS formulation the response becomes apparent only at higher light doses, with both formulations reaching comparable effects at the highest irradiation dose.

The bacteriochlorin derivative (TPF_5_B) also exhibits significant photodynamic activity (Figure 10a-c), despite its lower singlet oxygen quantum yield. This apparent discrepancy is consistent with preferential engagement in type I photochemical pathways, enabling efficient generation of radical species. Importantly, its absorption in the NIR makes it inherently more suitable for *in vivo* applications, where deeper tissue penetration and reduced background absorption are critical.^1^ Consistent with this, TPF_5_B demonstrates comparable or improved activity in CT26 relative to B16–F10 cells and was therefore selected for further *in vivo* evaluation. Its performance is strongly context-dependent: while *in vitro* efficacy appears moderate compared to porphyrin analogues, its full therapeutic potential emerges under conditions that better mimic the physiological environment.

To approximate vascular-targeted PDT conditions, experiments were conducted in 2H-11 (Figure 10c) endothelial cells using a short incubation time (15 min), representing a simplified V-PDT model *in vitro*, in which NIR-absorbing photosensitizers, commonly bacteriochlorins, are typically employed. Under these conditions, TPF_5_B induces an approximately 50% reduction in cell viability within 5 min of irradiation, indicating rapid photodynamic action consistent with V-PDT.

These findings demonstrate that photodynamic efficacy cannot be fully predicted from *in vitro* parameters alone and depends on the interplay among photophysical properties, formulation, and biological context. This is particularly evident for bacteriochlorins, whose performance may benefit from *in vivo* conditions such as tissue penetration, blood circulation, and tumor perfusion and, most notably, oxygen availability, underscoring the importance of evaluating photosensitizers under conditions relevant to their intended therapeutic application.

### *In Vivo* Studies

On the basis of the *in vitro* results, the pentafluorinated bacteriochlorin (TPF_5_B) formulated in P123 micelles was selected for *in vivo* evaluation in CT26 tumor-bearing BALB/c mice following intravenous administration (1.5 mg/kg).

Whole-body fluorescence imaging (Figure 11a) revealed a rapid systemic distribution of the photosensitizer, followed by progressive accumulation in tumor tissue over time. While initial fluorescence is predominantly observed at the injection site, a distinct signal in the tumor emerges within the first hours and increases markedly at 24 h post-injection. Notably, strong tumor-associated fluorescence persists for up to 96 h, indicating prolonged retention of the TPF_5_B within the tumor region. This extended retention reflects both the intrinsic stability of the fluorinated bacteriochlorin and the stabilizing effect of the micellar formulation. Fluorination has been reported to enhance metabolic stability by reducing susceptibility to oxidative degradation, for example, via cytochrome P450-mediated pathways, thereby prolonging circulation time and improving pharmacokinetic profiles.^64,65^ In combination with Pluronic P123 formulation, this results in sustained tumor accumulation, which is critical for effective photodynamic therapy.

**Figure 11:**
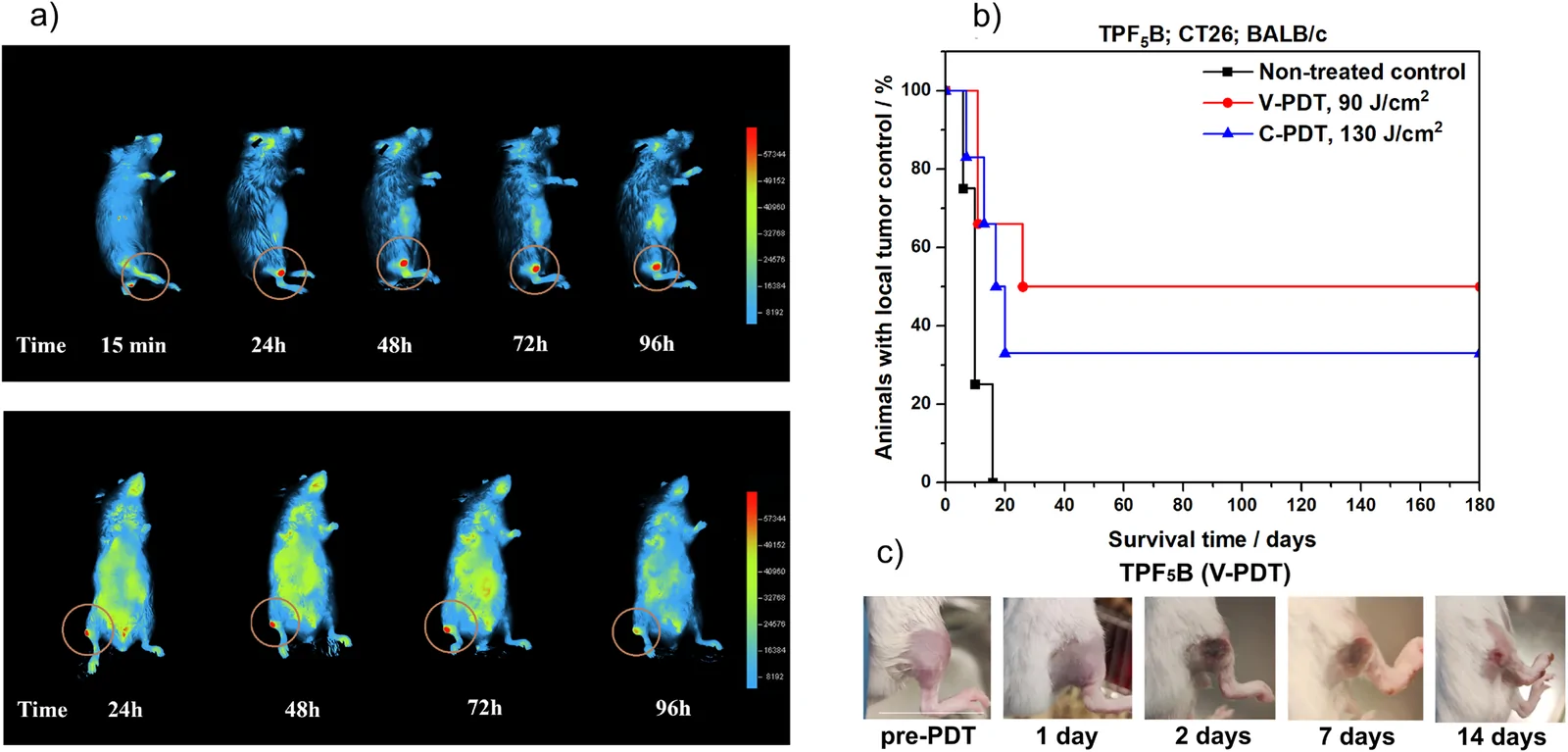
(a) Whole-body fluorescence imaging of BALB/c mice bearing CT26 tumors in the right hind limb, acquired at 15 min, 24, 48, 72, and 96 h after i.v. administration of 1.5 mg/kg TPF_5_B–P123. Imaging was performed with an excitation wavelength of 680 nm and fluorescence emission at 740–780 nm. (b) Kaplan–Meier analysis after PDT with TPF_5_B formulated in Pluronic P123 micelles against CT26 tumors in the BALB/c mice model, in two protocols: V-PDT (DLI = 15 min) and C-PDT (DLI = 24 h). (c) Representative photographs showing PDT-treated tumors: before the treatment, 1 day after, 2 days after, 1 week after, and 2 weeks after PDT, indicating the inflammatory processes, formation of necrotic tissue and a scab, and healthy tissue regeneration.

Therapeutic efficacy was evaluated using both vascular-targeted (V-PDT, DLI = 15 min, 90 J/cm^2^) and cellular-targeted (C-PDT, DLI = 24 h, 130 J/cm^2^) protocols (Figure 11b). The Kaplan–Meier analysis of local tumor controldemonstrates that V-PDT leads to long-term survival with a 50% cure rate, whereas C-PDT achieves approximately 33% cures.

The high efficacy observed for the vascular protocol can be attributed to the presence of the photosensitizer in the bloodstream shortly after administration, enabling efficient vascular damage upon irradiation. This behavior is consistent with known bacteriochlorin-based systems such as redaporfin and padeliporfin (Tookad Soluble).^66–69^

C-PDT protocol relies on tumor accumulation and retention of the photosensitizer, which may be partially attributed to enhanced permeability and retention-like effects (EPR) and supported by micellar transport.^70,71^ The longer drug-to-light interval enables deeper penetration into the tumor tissue and effective intracellular photodamage. In line with the *in vitro* localization data, ER-associated accumulation may contribute to stress signaling after irradiation, potentially promoting regulated cell death pathways including immunogenic cell death-related responses. These results demonstrate that the investigated bacteriochlorin is effective under both the V-PDT and C-PDT protocols.

This dual activity distinguishes TPF_5_B from many bacteriochlorin-based photosensitizers, which are typically optimized for vascular-targeted PDT. TPF_5_B combines early vascular damage with sustained tumor retention, supporting photodynamic efficacy also at extended drug-to-light intervals. Further studies are required to elucidate the relationship among pharmacokinetics, tumor microenvironment, and therapeutic outcomes.

## Summary

This study provides insight into the relationship among molecular structure, excited-state dynamics, and photodynamic efficacy in a series of perfluorinated tetrapyrrolic photosensitizers. All investigated compounds exhibit high photostability, further improved by micellar encapsulation, supporting activity under biologically relevant conditions. Formulation studies further showed that encapsulation efficiency, distribution coefficient (logD_7.4_), particle size, and stability mutually influence photosensitizer dispersion and light-induced ROS generation. Time-resolved measurements performed in Pluronic P123 micelles revealed prolonged triplet-state lifetimes compared with those in organic solvents, highlighting the role of the local microenvironment in modulating excited-state behavior.

Metal coordination was found to strongly influence the photophysical properties. Incorporation of Pd­(II) enhances intersystem crossing but leads to shorter triplet-state lifetimes, which may limit diffusion-controlled interactions with molecular oxygen under biologically relevant conditions. In contrast, Zn­(II) provides a more favorable balance between efficient triplet-state population and lifetime, supporting energy-transfer processes relevant for singlet oxygen generation. This highlights that efficient triplet formation does not necessarily translate to optimal photodynamic performance.

To further assess how these differences manifest under biologically relevant conditions, ROS generation was additionally evaluated in 1% DMSO/PBS and after encapsulation in Pluronic P123 micelles. In these formulation-matched experiments, the generation of singlet oxygen, superoxide anion radical, and hydroxyl radical was monitored under identical irradiation conditions, enabling direct comparison of ROS profiles while accounting for environmental effects, such as aggregation, oxygen accessibility, and local microenvironment.

The bacteriochlorin derivative TPF_5_B exhibits a distinct photochemical profile with a pronounced contribution of type I pathways, associated with enhanced charge-transfer interactions and efficient oxygen-mediated quenching of the triplet state. Despite lower singlet oxygen quantum yields and moderate *in vitro* activity, TPF_5_B shows enhanced ROS generation in the Pluronic P123 formulation, favorable ER association localization, and pronounced *in vivo* performance. Its NIR absorption, prolonged tumor retention, and activity under both vascular- and cellular-targeted PDT protocols support its dual-mode therapeutic potential. These findings underscore the importance of evaluating photosensitizers within the full biological context rather than relying solely on singlet oxygen quantum yields or short-term *in vitro* potencies.

The data demonstrate that efficient photosensitizer design requires not only effective triplet-state formation but also control over triplet excited-state lifetime, oxygen-mediated quenching, and formulation-dependent microenvironmental effects. The synthetic accessibility and tunable photophysical properties of these perfluorinated tetrapyrroles support their further development as versatile photosensitizer platforms.

## Experimental Section

### General Information

All reagents and solvents were obtained from commercial suppliers and used as received unless otherwise stated. All reactions were carried out under an inert atmosphere where required and protected from light. Nuclear magnetic resonance (NMR) spectra were recorded on a 400 MHz spectrometer, and chemical shifts (δ) are reported in ppm relative to residual solvent signals. High-resolution mass spectra (HRMS) were obtained using electrospray ionization (ESI). Electronic absorption spectra were recorded in appropriate solvents as specified. Purifications were performed using column chromatography on silica gel or preparative thin-layer chromatography as indicated.

### Synthesis of 5,10,15,20-Tetrakis­(pentafluorophenyl)­porphyrin

A mixture of pyrrole (0.5 mL, 1 equiv) and pentafluorobenzaldehyde (1.5 g, 1 equiv) was dissolved in 350 mL of dichloromethane (DCM) and stirred under an inert atmosphere for 15 min. Boron trifluoride diethyl etherate (BF_3_·OEt_2_, 0.1 mL) was then added, and the reaction mixture was stirred for 1 h under the same conditions. Subsequently, 2,3-dichloro-5,6-dicyano-1,4-benzoquinone (DDQ, 0.76 g) was introduced, and stirring was continued for 1 h under ambient atmosphere. The reaction mixture was left to crystallize in a cool, dark place for 48 h. The crude product was purified by column chromatography using hexane/DCM (1:1) as the eluent. The reaction was performed under light-protected conditions, affording the desired porphyrin in 14% yield. ^1^H NMR (400 MHz, CDCl_3_) δ 8.92 (s, 8H-β), −2.92 (s, 2H-NH). HRMS (ESI^–^): *m*/*z* [M–H]^−^ calculated for C_44_H_10_F_20_N_4_: MW = 974.56, found: 973.06 (Figures S17–S19).

### Metalation of 5,10,15,20-Tetrakis­(pentafluorophenyl)­porphyrin

A solution of 5,10,15,20-tetrakis­(pentafluorophenyl)­porphyrin (0.021 mmol, 1 equiv), zinc acetate (0.31 mmol), or palladium acetate (0.28 mmol, 10 equiv) in anhydrous DMF (20 mL) was stirred under an inert atmosphere. The reaction mixture was heated to 150 °C for 2 h. Reaction progress was monitored by UV–vis-NIR electronic spectroscopy, with completion indicated by the transformation of four Q bands into two, confirming metal insertion into the porphyrin core. After completion, the solvent was removed under reduced pressure, and the residue was dissolved in a 1:1 mixture of DCM and chloroform. The organic phase was washed three times with 10% NaHCO_3_ solution and twice with distilled water, dried over anhydrous Na_2_SO_4_, filtered, and concentrated to afford the zinc and palladium porphyrin derivatives. The reaction was carried out under light-protected conditions, giving yields of 93% and 89%, respectively. TPF_5_P Zn: ^1^H NMR (400 MHz, CDCl_3_) δ 8.88 (s, 8H-β). HRMS (ESI^+^): *m*/*z* [M + Na]^+^ calculated for C_44_H_8_F_20_N_4_Zn: 1060.92, found: 1061.92 (Figures S20–S22). TPF_5_P Pd: ^1^H NMR (400 MHz, CDCl_3_) δ 8.89 (s, 8H-β). HRMS (ESI^+^): *m*/*z* [M + Na]^+^ calculated for C_44_H_8_F_20_N_4_Pd: 1101.96, found: 1100.98 (Figures S23–S25).

### Synthesis of 5,10,15,20-Tetrakis­(pentafluorophenyl)­bacteriochlorin

5,10,15,20-Tetrakis­(pentafluorophenyl)­porphyrin (0.030 mmol, 1 equiv) and *p*-toluenesulfonic acid hydrazide (2.000 mmol, 40 equiv) were thoroughly ground under solvent-free conditions using a mortar and pestle. The mixture was transferred to a round-bottom flask and connected to a Schlenk line. Air was removed by repeated evacuation and refilling with argon. The system was then kept under vacuum for 1 h and heated at 140 °C for approximately 3 h. The crude product was purified by preparative TLC using ethyl acetate/*n*-hexane (1:10). The upper fraction corresponded to pure bacteriochlorin. ^1^H NMR (400 MHz, CDCl_3_) δ 8.12 (d, J = 1.9 Hz, 4H-β), 4.12 (s, 8H-β), −1.44 (s, 2H-NH). HRMS (ESI^–^): *m*/*z* [M–H]^−^ calculated for C_44_H_14_F_20_N_4_: MW = 978.59, found: 977.08. Anal. Calculated for C_44_H_14_F_20_N_4_: C, 54.0%; H, 1.4%; N, 5.7%. Found: C, 54.8%; H, 3.7%; N, 4.8%. HPLC *t*_R_ = 24.5 min. The sample elution performed in gradient elution, solvent B increased from 50% to 95% over 35 min; solvent A: 0.1 M ammonium acetate in Milli-Q water; solvent B: acetonitrile (MeCN) (Figures S26–S29).

All compounds are >95% pure as determined by NMR, MS, and HPLC analysis.

### Pluronic P123 Micelles Preparation

Pluronic P123 (9 mg) was dissolved in ethanol (1 mL) with gentle heating to ensure complete dissolution. Subsequently, an appropriate volume of the PS stock solution was added to obtain a final concentration of 200 μM upon further solution in PBS. The solvent was then removed under reduced pressure using a rotary evaporator, yielding a thin film. Sterile PBS (1 mL), or deionized water for **ζ**-potential measurements, was added to rehydrate the film, resulting in a final PS concentration of 200 μM. The obtained formulation was used directly for further experiments.

### DLS Hydrodynamic Diameter and ζ-Potential Measurements

The hydrodynamic diameter and ζ-potential of photosensitizer-loaded Pluronic P123 micelles were determined using dynamic light scattering (DLS) on a Zetasizer (Malvern Instruments). The stock formulation (200 μM) was diluted 10-fold prior to measurements.

### Encapsulation Efficiency

Encapsulation efficiency of the investigated compound within Pluronic P123 micelles was quantified by UV–vis spectrophotometry. Following self-assembly, the micellar suspension was filtered through a sterile 0.22 μm syringe filter to remove non-encapsulated aggregates. An aliquot of the purified micellar solution was then diluted 20-fold in DMSO and subjected to ultrasonication for 2 min to disrupt the micelles and release the encapsulated compound into its monomeric form. The absorbance was recorded at the Soret band maximum, and the concentration of the released compound was determined using a previously established calibration curve (2–20 μM) in DMSO. The encapsulation efficiency (EE%) was calculated as the ratio of the experimentally determined amount of compound in the filtered micelles to the theoretical amount used in the formulation.

### *n*-Octanol/PBS Distribution Coefficients at pH 7.4 (logD_7.4_)

Distribution coefficients at pH 7.4 (logD_7.4_) were determined using the shake-flask method. Calibration curves for each photosensitizer were prepared in two systems: 0.5% n-octanol in DMSO and 0.5% PBS in DMSO. The concentration range used for calibration was 0.5–100 nM (100, 75, 50, 25, 15, 10, 5, 1, 0.5 nM), based on fluorescence intensity measurements. Prior to measurements, mutually saturated solvent systems were prepared by mixing n-octanol and PBS and equilibrating overnight under agitation. For partitioning experiments, the investigated compound was dissolved in 5 mL of PBS-saturated n-octanol and sonicated at 35 °C until complete dissolution. The system was then equilibrated by adding n-octanol-saturated PBS, followed by shaking for 15 min to allow partitioning between phases. After equilibration, samples were centrifuged (3700 rpm, 3 min) to ensure complete phase separation. Aliquots (20 μL) from each phase were collected and diluted in 3.98 mL of DMSO. Emission spectra were recorded using excitation wavelengths appropriate for each compound.

### Electronic Absorption and Emission Spectroscopy

Electronic absorption spectra were recorded using a Shimadzu UV-1900i spectrophotometer in the range of 300–800 nm in a 1 cm quartz cuvette. Emission spectra were measured using a Shimadzu RF-6000 spectrofluorometer over the range of 500–800 nm under the same conditions. Molar absorption coefficients were determined from a series of absorption measurements performed in DMSO, Pluronic P123 micelles in PBS, and 1% DMSO in PBS. The measurements were carried out over a concentration range of 1–200 μM. Only absorbance values in the range of 0.1–1.0 were used for constructing calibration curves.

### Photodecomposition Quantum Yields

Photodecomposition quantum yields (Φ_PD_) were determined for photosensitizers dissolved in DMSO, PBS containing Pluronic P123 micelles, and 1% DMSO in PBS. A 2 mL aliquot of each solution was placed in a cuvette and irradiated under conditions ensuring complete absorption of the incident light. Porphyrins were irradiated at 420 ± 20 nm, whereas bacteriochlorins were irradiated at 750 ± 5 nm using a light source with an output power of 17 mW·cm^–2^. The cuvettes were sealed during irradiation to prevent solvent evaporation and maintain a constant concentration. The photodecomposition quantum yield (Φ_PD_) is defined as the rate of photosensitizer molecule degradation (*N*_d_) to the rate of photon absorption (*N*_p_):

$$\Phi_{PD}=\frac{N_{d}}{N_{p}}$$

To determine the number of absorbed photons, the absorption spectrum of the photosensitizer was compared with the emission spectrum of the light source. The overlap region was used to calculate the effective absorbed light intensity (*I*_abs_). The spectral irradiance of the light source, *I*(λ), and the absorbance of the photosensitizer, (*A*(*λ*)), were used according to

$$I_{abs}=I\left(\lambda \right)\times \left(1-10^{-A\left(\lambda \right)}\right)$$

The energy of a photon (*E*_p_) is given by

$$E_{p}=\frac{hc}{\lambda }$$

where *h* is Planck’s constant, *c* is the speed of light, and λ is the wavelength.

The photon flux (*N*_p_) was calculated from the absorbed irradiance:

$$N_{p}=\frac{I_{abs}}{E_{p}}=\frac{I_{abs}\times \lambda }{hc}$$

The number of degraded molecules (*N*_d_) was determined from changes in absorbance using molar absorption coefficients according to the Beer–Lambert law.

During irradiation, the sample temperature was monitored under the applied experimental conditions. No noticeable temperature increase was observed, and all measurements were conducted under near-ambient conditions. Therefore, thermal effects on the photodecomposition or micellar stability are considered negligible.

### Transient Absorption Spectroscopy and Triplet-State Lifetime Measurements

Transient absorption spectra were recorded using a transient absorption spectrometer (Instytut Fotonowy) equipped with an Nd:YAG laser (Q-smart, Quantel), an R928P (Hamamatsu) photomultiplier, and a 6403E oscilloscope (PicoScope). During the measurements, the samples were excited with a 355 nm laser pulse (100 mJ, 10 Hz, pulse duration 6 ns), while a pulsed Xe lamp was used as the probe light source. Measurements were performed at room temperature (20 °C) under both ambient and inert (argon) atmospheres. To remove dissolved oxygen, tetrapyrrole solutions were purged with argon prior to measurements. Decay curves and transient absorption spectra were recorded in ethanol, DMSO, and Pluronic P123 micelles in PBS.

### Singlet Oxygen Phosphorescence Measurements

Phosphorescence measurements were performed using a Fluorolog-3 spectrofluorometer (Horiba-Jobin Yvon) equipped with a 450 W CW xenon lamp as the excitation source and an InGaAs DSS IR FLIGA 1.7 detector (Horiba) operating in the 800–1700 nm range. Singlet oxygen phosphorescence spectra were recorded in ethanol for the investigated porphyrins and bacteriochlorin by using a 1 cm quartz cuvette. The excitation wavelengths were set to 412 nm for porphyrins and 507 nm for bacteriochlorin.

### Reactive Oxygen Species Detection

Photosensitizer solutions were prepared in 1% DMSO in PBS at a concentration of 5 μM. Aliquots (100 μL) were transferred into separate wells of a 96-well plate, followed by the separate addition of APF, DHE, or DMA to a final concentration of 10 μM. The same procedure was applied to the corresponding Pluronic P123 formulations. DHE was used as a superoxide-related ROS probe, APF as a hydroxyl-radical/hROS probe, and DMA as a singlet oxygen-sensitive probe. APF shows an approximately two-orders-of-magnitude higher fluorescence response toward ^•^OH than toward ^1^O_2_ or O_2_^•–^ and negligible response toward H_2_O_2_, NO, and ROO^•^ under reported reference conditions; therefore, under the applied cell-free photochemical conditions, the APF signal was interpreted primarily as a hydroxyl radical-related readout. Fluorescence intensity was measured using an Infinite M200 plate reader (Tecan), with emission recorded at 515 nm upon excitation at 490 nm (for APF probe), emission recorded at 430 nm upon excitation at 365 nm (for DMA probe), and emission recorded at 606 nm upon excitation at 518 nm (for DHE probe). Singlet oxygen generation was also evaluated using 9,10-dimethylanthracene (DMA) as a selective chemical probe at a final concentration of 2 μM in DMF. Photosensitizers were used at a concentration of 5 μM in a total volume of 2 mL. Porphyrin-containing solutions were irradiated with blue light (420 ± 20 nm), whereas bacteriochlorin-containing samples were irradiated with a light-emitting diode (750 ± 5 nm, Instytut Fotonowy, Poland). The irradiation conditions were adjusted to deliver an energy dose of 1 J·cm^–2^ within 60 s. Fluorescence emission spectra of DMA were recorded using a Shimadzu RF-6000 spectrofluorometer in a 1 cm quartz cuvette, with excitation at 363 nm and emission collected in the 385–600 nm range. A progressive decrease in DMA fluorescence intensity was observed over time and fitted to an exponential decay function:

$$y=y_{0}+Ae^{R_{0}x}$$

where *y* represents fluorescence intensity at time *t*, *A* is the amplitude, and *R*_0_ is the rate constant of fluorescence decay. The singlet oxygen quantum yield (Φ_Δ_) of each compound was determined relative to reference photosensitizers using the equation:

$$\Phi_{\Delta }^{\mathrm{sample}}=\Phi_{\Delta }^{Ref}\times \frac{\left|R_{0}^{\mathrm{sample}}\right|\times A^{\mathrm{sample}}}{\left|R_{0}^{Ref}\right|\times A^{Ref}}$$

F_2_PMet and F_2_BMet in ethanol were used as reference compounds with known singlet-oxygen quantum yields.

Hydroxyl radicals were additionally detected by EPR spin trapping using DMPO as a spin trap. Samples contained 5 μM photosensitizer and 50 mM DMPO. EPR measurements were performed at room temperature using a Bruker ESP 300 spectrometer (IBM Instruments Inc.), with spectra recorded under in situ irradiation using a 750 ± 5 nm laser for 20 min.

### Statistical Analysis

Statistical analysis was performed using two-way ANOVA followed by Dunnett’s or Šidák’s multiple-comparisons test, as appropriate. Šidák’s test was used for intracellular uptake comparisons. *In vitro* data are presented as mean ± SD from four independent experiments (*n* = 4). Statistical significance was defined as *p < 0.05, **p < 0.01, and ***p < 0.001.

### Cell Culture

Murine colon carcinoma (CT26), murine melanoma (B16–F10), and murine endothelial (2H-11) cells were cultured in RPMI 1640 medium (Gibco) supplemented with 10% fetal bovine serum (FBS, BioTech, Poland) and 1% penicillin/streptomycin (Gibco). Cells were maintained at 37 °C in a humidified atmosphere with 5% CO_2_ and grown as monolayers. Prior to experiments, cells were detached by trypsinization, washed with PBS without Ca^2+^ and Mg^2+^, and passaged every 2–3 days to maintain exponential growth. For experiments in 96-well plates, cells were seeded at a density of ∼1000 cells per well.

### Cellular Uptake

CT26 and B16–F10 cells were incubated with photosensitizers (20 μM) in two formulations: (i) dissolved in 1% DMSO in RPMI 1640 and (ii) encapsulated in Pluronic P123 micelles. Uptake was monitored over 24 h at selected time points (2, 4, 6, 8, 12, 18, and 24 h). After incubation, cells were washed twice with warm PBS containing Ca^2+^ and Mg^2+^ and lysed using 30 μL of 1% Triton X-100 and 70 μL of DMSO/ethanol (1:3, v/v). Intracellular accumulation was quantified by fluorescence measurements using a Tecan Infinite M200 plate reader, with excitation at the absorption maxima of each compound (TPF_5_P: 411 nm, TPF_5_P Zn: 422 nm, TPF_5_B: 503 nm). The concentration of the photosensitizers in cell lysates was determined from calibration curves by correlating measured fluorescence intensities with the corresponding concentrations obtained under identical instrumental conditions. The concentration was normalized to the number of cells seeded per well.

### Intracellular Localization

Microscope slides were sterilized with 70% ethanol for 20 min, rinsed with PBS containing Ca^2+^ and Mg^2+^, and transferred to 12-well culture plates. CT26 cells were then seeded onto the slides at a density of 2.5 × 10^3^ cells per well and incubated for 24 h. Following incubation, cells were exposed to either 5 μM TPF_5_B dissolved in 1% DMSO in cell culture medium or TPF_5_B-loaded P123 micelles for periods ranging from 3 to 24 h. After treatment, the cells were washed, fixed with 0.5% paraformaldehyde, and permeabilized using 0.1% Triton X-100. To visualize intracellular organelles, fluorescent markers from Invitrogen were employed: α-LAMP-1-eFluor660 for lysosomes, α-MTCO1-Alexa Fluor 555 for mitochondria, α-GM130-Alexa Fluor 594 for the Golgi apparatus, and DiOC_6_(3) for the endoplasmic reticulum. Confocal imaging was carried out on a Zeiss LSM 780 microscope equipped with a 63×/1.4 NA oil-immersion objective and the appropriate laser excitation and emission filter settings. Image processing and colocalization analyses were performed using ImageJ software with the JACoP plugin.^59^

### Intracellular ROS Generation (APF Assay)

CT26 and B16–F10 cells were incubated with photosensitizers (5 μM) in two formulations: (i) dissolved in 1% DMSO in RPMI 1640 and (ii) encapsulated in Pluronic P123 micelles for 24 h. Two hours before the end of incubation, APF (Invitrogen) was added to a final concentration of 15 μM (total volume 100 μL). Cells were then washed with PBS containing Ca^2+^ and Mg^2+^ and irradiated (420 ± 20 nm for porphyrins; 750 ± 5 nm for bacteriochlorins) for varying time intervals. APF fluorescence (λ_exc_ = 495 nm, λ_em_ = 515 nm) was measured using a Tecan Infinite M200 microplate reader immediately before and after irradiation.

### Dark Cytotoxicity

Cells were seeded in 96-well plates and, after 24 h, incubated with photosensitizers (0.5–20 μM) in two formulations: (i) dissolved in 1% DMSO in RPMI 1640 and (ii) encapsulated in Pluronic P123 micelles. After 18–22 h incubation, corresponding to the cell doubling time, viability was assessed using the AlamarBlue assay. Resazurin solution (10 μL, 0.1 mg/mL) was added to each well, and fluorescence was measured using a Tecan Infinite M200 reader (λ_em_ = 605 nm). Cell viability was expressed as a percentage relative to untreated controls. All procedures were performed under light-limited conditions.

### Photodynamic Activity

Cells were seeded in 96-well plates and incubated with photosensitizers (2.5–20 μM) in two formulations: (i) dissolved in 1% DMSO in RPMI 1640 and (ii) encapsulated in Pluronic P123 micelles for 24 h. After incubation, the medium was removed, and cells were washed with PBS containing Ca^2+^ and Mg^2+^. Cells were irradiated in PBS (Ca^2+^/Mg^2+^) at 420 ± 20 nm (porphyrins) or 750 ± 5 nm (bacteriochlorins), with light doses controlled by varying irradiation time (0–20 min). After irradiation, PBS was replaced with fresh culture medium, and cells were incubated for an additional 24 h. Cell viability was determined using the AlamarBlue assay as described above. For the 2H-11 cell line, the same protocol was applied with a shorter incubation time (15 min) and irradiation performed directly in RPMI 1640 medium instead of PBS.

### Animal Model

All animal experiments were conducted in accordance with protocols approved by the First Local Ethics Committee for Animal Research in Kraków, Poland (permission no. 242/2022). Male BALB/c mice (7–8 weeks old, AnimaLab) were used for *in vivo* studies. Animals were housed under specific pathogen-free (SPF) conditions in individually ventilated cages at 22 ± 2 °C, 50–60% humidity, and a 12 h light/dark cycle, with food and water provided ad libitum.

### Fluorescence Imaging

When tumors reached approximately 5 mm in diameter, mice received intravenous injections of TPF_5_B–P123 at a dose of 1.5 mg/kg of body weight. Whole-body fluorescence imaging was performed by using a Newton 7.0 system (Vilber, France) immediately after administration and over the following 96 h. Imaging was conducted using excitation at 680 nm and emission detection in the 740–780 nm range. Animals were anesthetized with 3–4% isoflurane (Aerrane, Baxter, Poland) during imaging.

### Photodynamic Therapy (PDT)

CT26 tumor cells (3.5 × 10^5^) were injected subcutaneously into the right hind limb of BALB/c mice. PDT was initiated when tumors reached 4–5 mm in diameter (typically 7–10 days postinoculation). Mice were randomly assigned to treatment groups (*n* = 6) and received intravenous administration of TPF_5_B–P123. Laser irradiation (748 nm; LDM750.300.CWA.L.M, equipped with an optical fiber FD/Medlight, Ecublens, Switzerland) was applied using two drug-to-light intervals (DLIs): 15 min (90 J·cm^–2^) and 24 h (130 J·cm^–2^). The laser spot size was maintained at 2 cm^2^. Tumor growth, body weight, and animal behavior were monitored regularly. Animals were euthanized when the tumor size exceeded 10 mm. Survival was analyzed using the Kaplan–Meier method.

## Supplementary Material





## Notes

Molecular strings (SMILES) for TPF5P, TPF5P Zn, TPF5P Pd, and TPF5B (CSV)
Calibration curves for concentration determination and encapsulation efficiency; stability of photosensitizer-loaded Pluronic P123 micelles; calibration curves for distribution coefficients (logD_7.4_); electronic absorption and emission spectra in DMSO and 1% DMSO in PBS; photodegradation kinetics in different formulations; triplet-state lifetime measurements in air- and argon-saturated solutions; laser flash photolysis decay profiles; singlet oxygen phosphorescence spectra; DMA fluorescence decay and singlet oxygen generation curves; ROS detection using DMA, DHE, and APF probes; influence of DMSO concentration on hydroxyl radical generation; calibration curves for cellular uptake; confocal fluorescence imaging of TPF5B in solution and micellar formulation; ^1^H NMR and ^19^F NMR spectra; ESI mass spectra with simulated isotope patterns; HPLC traces of TPF5B (PDF)

## Abbreviations

2H-11: murine endothelial cell line
APF: aminophenyl fluorescein
B16–F10: murine melanoma cells
BALB/c: inbred mouse strain
C-PDT: cellular-targeted photodynamic therapy
CT: charge transfer
CT26: murine colon tumor cells
DAD: diode array detection
DCM: dichloromethane
DDQ: 2,3-dichloro-5,6-dicyano-1,4-benzoquinone
DHE: dihydroethidium
DLI: drug-to-light interval
DLS: dynamic light scattering
DMA: dimethylanthracene
DMF: dimethylformamide
DMPO: 5,5-dimethylpyrroline N-oxide
DMSO: dimethyl sulfoxide
EE%: encapsulation efficiency
EPR: electron paramagnetic resonance
EPR effect: enhanced permeability and retention
ER: endoplasmic reticulum
ESI: electrospray ionization
EtOH: ethanol
FBS: fetal bovine serum
HOMO: highest occupied molecular orbital
HPF: hydroxyphenyl fluorescein
HPLC: high-performance liquid chromatography
HRMS: high-resolution mass spectrometry
ICD: immunogenic cell death
i.v.: intravenous
ISC: intersystem crossing
logD_7.4_: n-octanol/PBS distribution coefficients at pH 7.4
LUMO: lowest unoccupied molecular orbital
MeCN: acetonitrile
NIR: near-infrared
NMR: nuclear magnetic resonance
PBS: phosphate-buffered saline
PDT: photodynamic therapy
PEO: poly­(ethylene oxide)
PPO: poly­(propylene oxide)
PS: photosensitizer
ROS: reactive oxygen species
SPF: specific pathogen-free
TLC: thin-layer chromatography
*t*_R_: retention time
UPR: unfolded protein response
V-PDT: vascular-targeted photodynamic therapy
ΔOD: delta optical density
ε: molar absorption coefficient
ε_max_: molar absorption coefficient at maximum absorbance
λ_em_: emission wavelength
λ_ex_: excitation wavelength
λ_max_: wavelength of absorption maximum
τ_T_: triplet state lifetime
Φ_F_: fluorescence quantum yield
Φ_PD_: photodecomposition quantum yield
Φ_Δ_: singlet oxygen quantum yield
