# Supplementary material for: From Porphyrins to Versatile Photostable Bacteriochlorins: Triplet-State-Driven Design of Perfluorinated Photosensitizers
Source: J Med Chem. 2026 Aug 8;69(16):19856–76. doi: 10.1021/acs.jmedchem.6c01282 (PMC13528295; doi:10.1021/acs.jmedchem.6c01282)
Supplement: Supplementary file 1 [file jm6c01282_si_002.pdf]

Supporting Information

From Porphyrins to Versatile Photostable Bacteriochlorins: Triplet-State-Driven Design of Perfluorinated Photosensitizers

Dominik Barczyk[1,2], Marta Warszyńska[1, 2], Paweł Repetowski[1, 2], Joanna Kuncewicz[1] and Janusz M. Dąbrowski[1,*]

[1] *Faculty of Chemistry, Jagiellonian University, 30-387 Kraków, Poland*

[2] *Doctoral School of Exact and Natural Sciences, Jagiellonian University, 30-348 Kraków, Poland*

*Correspondence should be sent to Janusz M. Dąbrowski, jdabrows@chemia.uj.edu.pl, +48126632293, +48126340515 (fax)

Table of Contents

Figure S1. Calibration curves for concentration determination (a, c, e, g) and corresponding absorbance of disrupted P123 micelles loaded with photosensitizers TPF_5P, TPF_5P Zn, TPF_5P Pd, and TPF_5B, respectively (b, d, f, h). **S3**

Figure S2. Time-dependent normalized absorption spectra of photosensitizer-loaded Pluronic P123 micelles dispersed in PBS: (a) TPF_5P, (b) TPF_5P Zn, (c) TPF_5P Pd, and (d) TPF_5B. Spectra were recorded over 7 days to assess formulation stability.... **S4**

Figure S3. Calibration curves related to values of distribution coefficient ($LogD_{7.4}$) for (a) TPF_5P, (b) TPF_5P Zn, and (c) TPF_5B **S5**

Figure S4. Normalized electronic absorption spectra of porphyrins TPF_5P, TPF_5P Pd, TPF_5P Zn, and bacteriochlorin TPF_5B recorded in 1% DMSO in PBS. **S6**

Figure S5. Normalized electronic absorption and emission spectra of synthesized photosensitizers (a) TPF_5P, (b) TPF_5P Zn, (c) TPF_5P Pd, and (d) TPF_5B, recorded in DMSO. **S6**

Figure S6. Photodegradation kinetics (molecular decay per photon). **S7**

Figure S7. Transient absorption decay traces used to determine triplet-state lifetimes of TPF_5P, TPF_5P Pd, TPF_5P Zn, and TPF_5B in air-saturated ethanol, DMSO, and Pluronic P123 micelles in PBS. **S8**

Figure S8. Transient absorption decay traces used to determine triplet-state lifetimes of TPF_5P, TPF_5P Pd, TPF_5P Zn, and TPF_5B in argon-saturated ethanol, DMSO, and Pluronic P123 micelles in PBS. **S9**

Figure S9. Phosphorescence spectra of singlet oxygen recorded in ethanol for porphyrins.. .... **S10**

Figure S10. DMA emission spectra recorded during irradiation with blue light (420 ± 20 nm) in the presence of TPF_5P Zn (a), and with NIR light (750 ± 5 nm) for TPF_5B (b). **S10**

Figure S11. Singlet oxygen generation curves related to DMA fluorescence decay, recorded in ethanol. **S11**

Figure S12. ROS detection by different probes: DMA (singlet oxygen), DHE (superoxide anion radical), APF (hydroxyl radical), respectively in 1% DMSO in PBS and P123 in PBS formulation for the discussed porphyrins TPF_5P, TPF_5P Pd, TPF_5P Zn. **S11**

Figure S13. Influence of DMSO concentration (0.1-3%) on hydroxyl radical generation monitored using the APF probe. . **S12**

Figure S14. Calibration curves for the assessment of cellular uptake. **S13**

Figure S15. Fluorescence confocal imaging of TPF_5B dissolved in DMSO and different cell organelles **S13**

Figure S16. Fluorescence confocal imaging of TPF_5B encapsulated in Pluronic P123 micelles and different cell organelles **S14**

Figure S17. ^{1}H NMR spectra of TPF_5P in $CDCl_3$-d. **S15**

Figure S18. ^{19}F NMR spectra of TPF_5P in $CDCl_3$-d. **S15**

Figure S19. ESI- mass spectra obtained for TPF_5P (MW = 974.56), along with a simulation obtained by ChemCalc.org. **S16**

Figure S20. ^1H NMR spectra of TPF_5P Zn in $CDCl_3$-d. **S17**

Figure S21. ^{19}F NMR spectra of TPF_5P Zn in $CDCl_3$-d. **S17**

Figure S22. ESI+ mass spectra obtained for TPF_5P Zn (MW = 1037.92), along with simulation obtained by ChemCalc.org. **S18**

Figure S23. ^1H NMR spectra of TPF_5P Pd in $CDCl_3$-d. **S19**

Figure S24. ^{19}F NMR spectra of TPF_5P Pd in $CDCl_3$-d **S19**

Figure S25. ESI+ mass spectra obtained for TPF_5P Pd (MW = 1078.96), along with simulation obtained by ChemCalc.org. **S20**

Figure S26. ^1H NMR spectra of TPF_5B in $CDCl_3$-d. **S21**

Figure S27. ^{19}F NMR spectra of TPF_5B in $CDCl_3$-d. **S21**

Figure S28. ESI- mass spectra obtained for TPF_5B (MW = 978.59), along with a simulation obtained by ChemCalc.org .. **S22**

Figure S29. Analytical HPLC chromatogram and UV-Vis spectrum of lead compound TPF_5B. **S23**

Supporting Information:

Encapsulation Efficiency

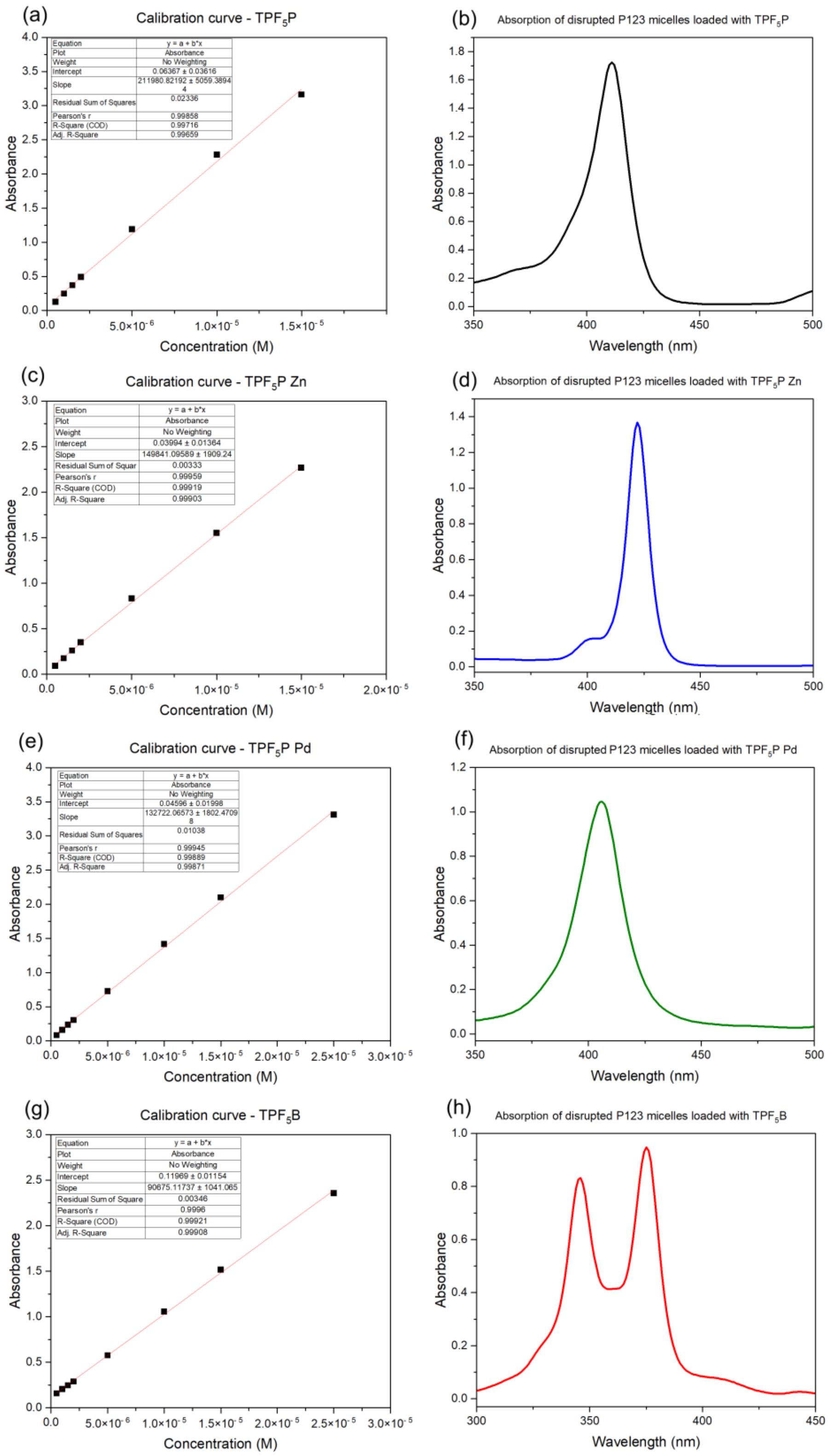

Figure S1. Calibration curves for concentration determination (a, c, e, g) and corresponding absorbance of disrupted P123 micelles loaded with photosensitizers TPF_5P, TPF_5P Zn, TPF_5P Pd, and TPF_5B, respectively (b, d, f, h), measured in DMSO and used to calculate encapsulation efficiency.

Pluronic P123 micelles stability over time

Formulation stability was assessed spectroscopically by monitoring time-dependent changes in the normalized absorption spectra of photosensitizer-loaded Pluronic P123 micelles dispersed in PBS.

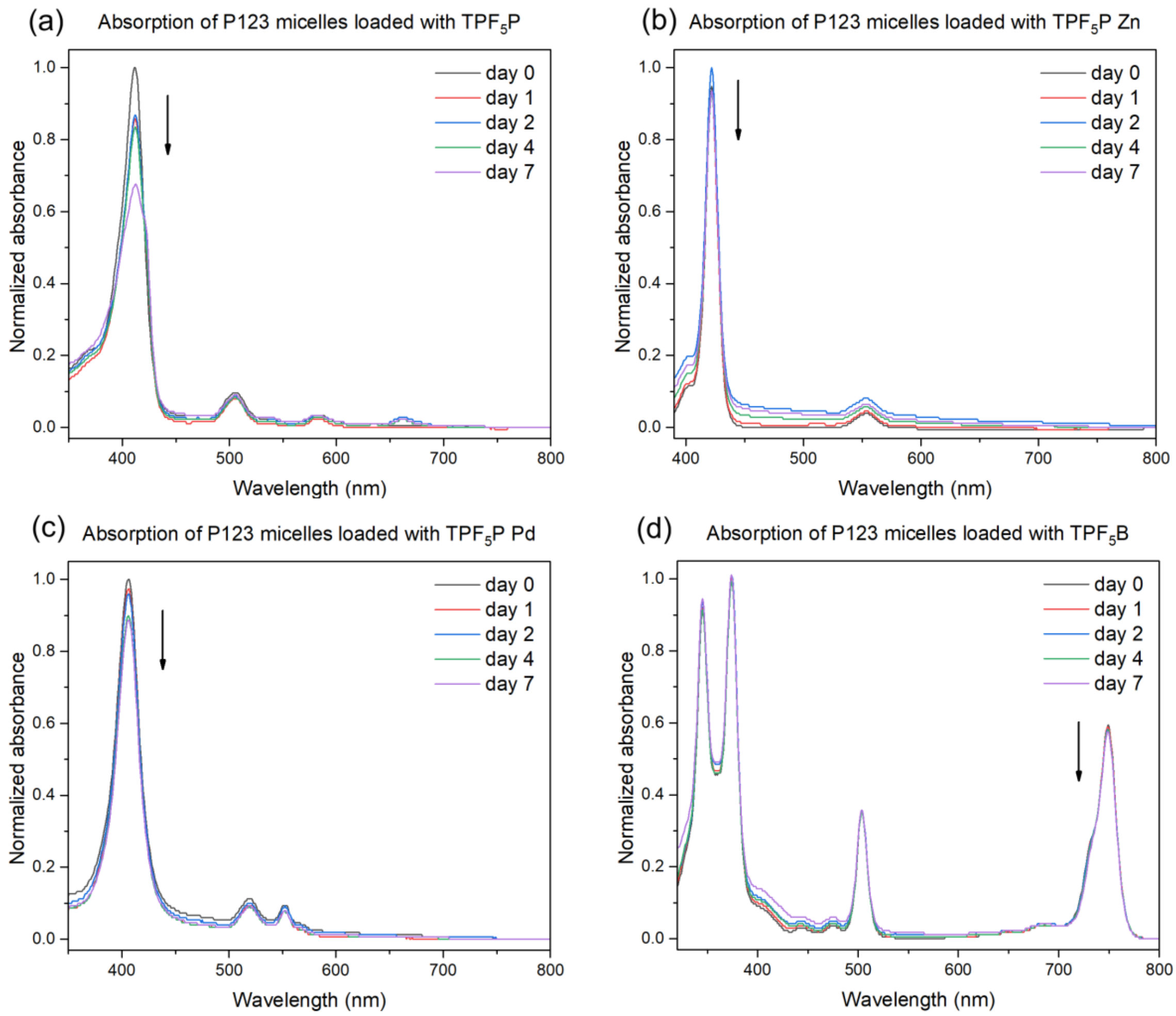

Figure S2. Time-dependent normalized absorption spectra of photosensitizer-loaded Pluronic P123 micelles dispersed in PBS: (a) TPF_5P, (b) TPF_5P Zn, (c) TPF_5P Pd, and (d) TPF_5B. Spectra were recorded over 7 days to assess formulation stability.

n-Octanol/PBS distribution coefficients at pH 7.4 (log$D_{7.4}$)

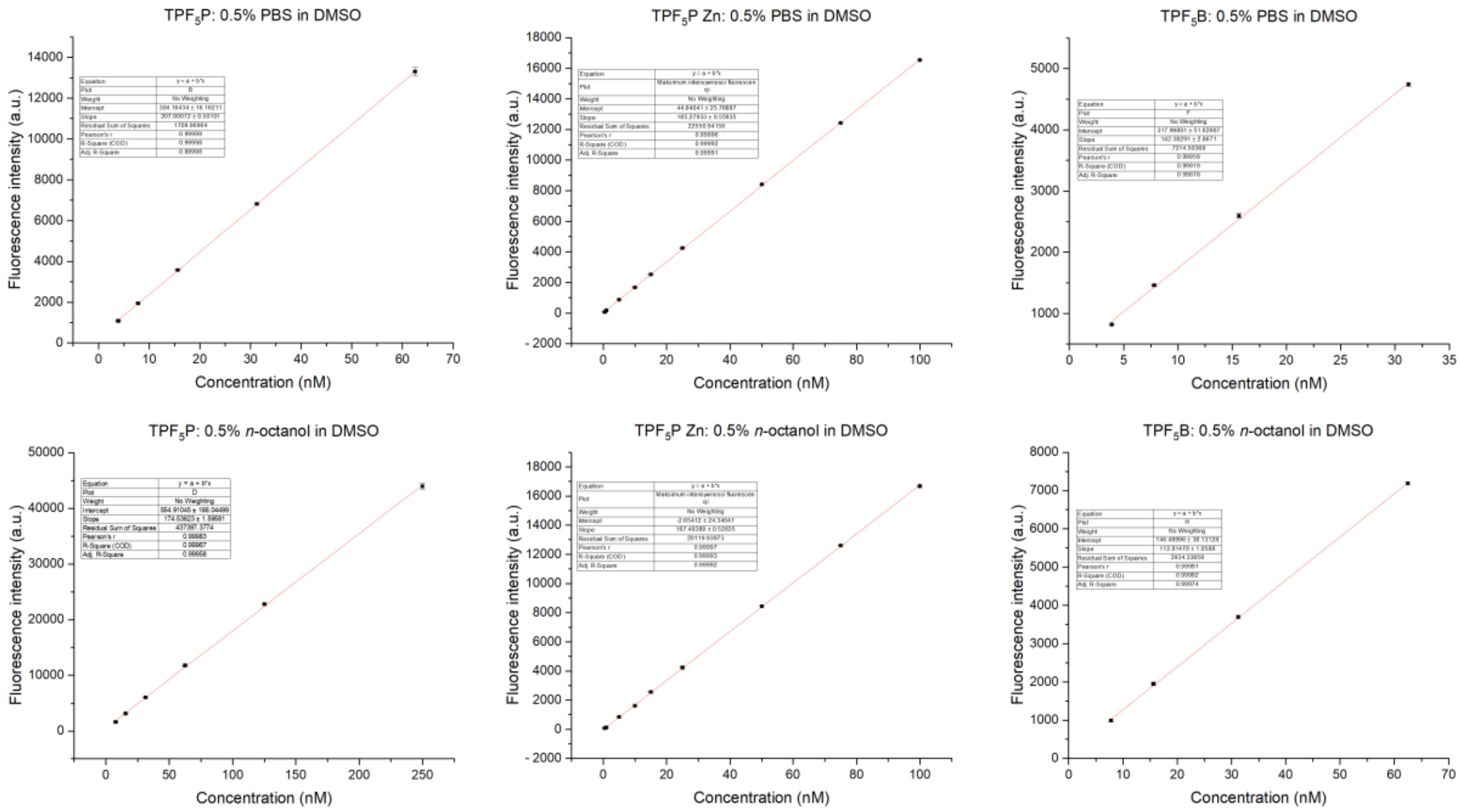

Figure S3. Calibration curves related to values of distribution coefficient (Log$D_{7.4}$) for (a) TPF_5P, (b) TPF_5P Zn, and (c) TPF_5B based on fluorescence intensity of photosensitizers in 0.5% solutions of PBS and n-octanol in DMSO.

Spectroscopic characterization of the synthesized photosensitizers

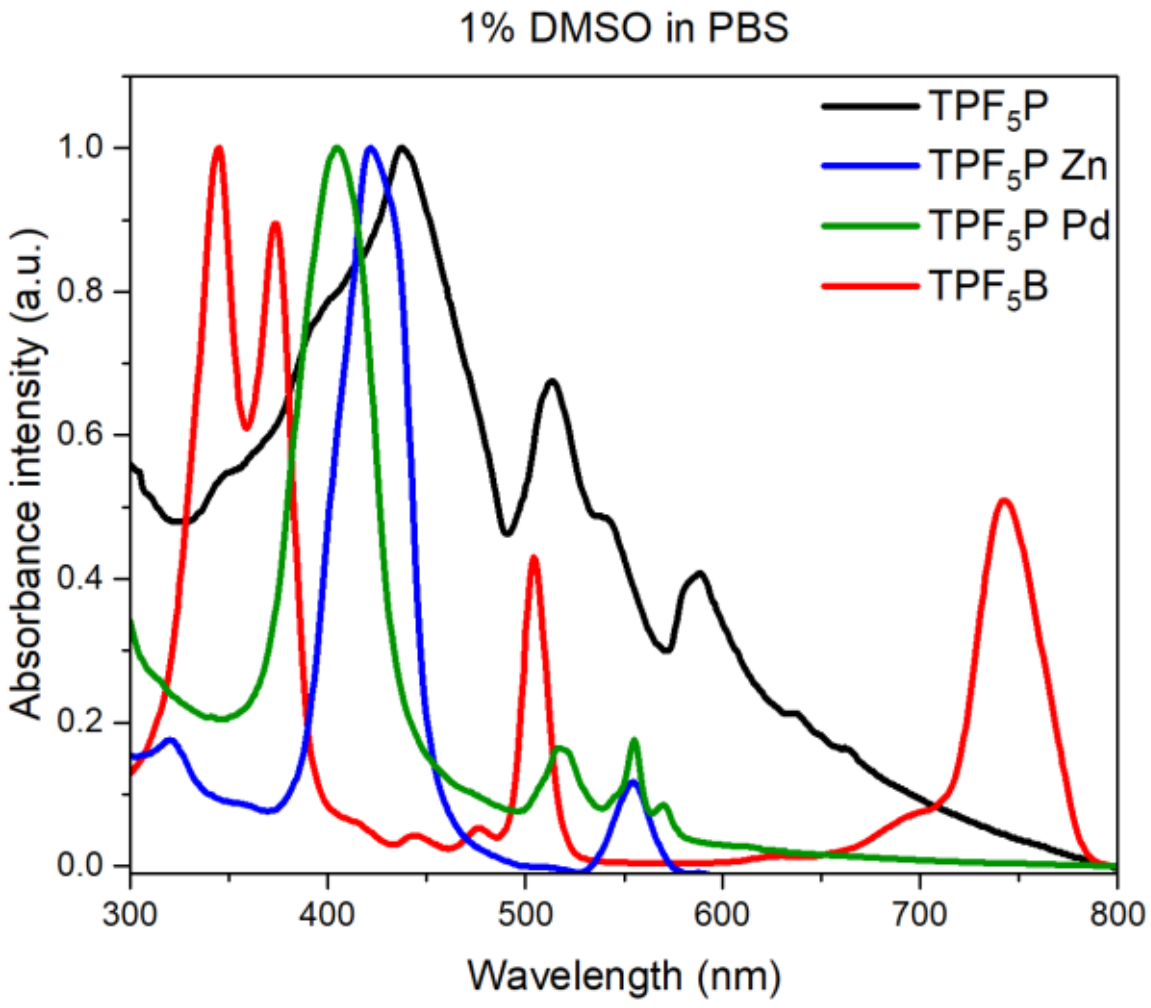

Figure S4. Normalized electronic absorption spectra of porphyrins TPF_5P, TPF_5P Pd, TPF_5P Zn, and bacteriochlorin TPF_5B recorded in 1% DMSO in PBS.

The observed spectral features for TPF_5P **(Figure S4)**, including band broadening and shifts of the Soret and Q bands, are indicative of π-π stacking interactions and support the presence of aggregated species in solution.

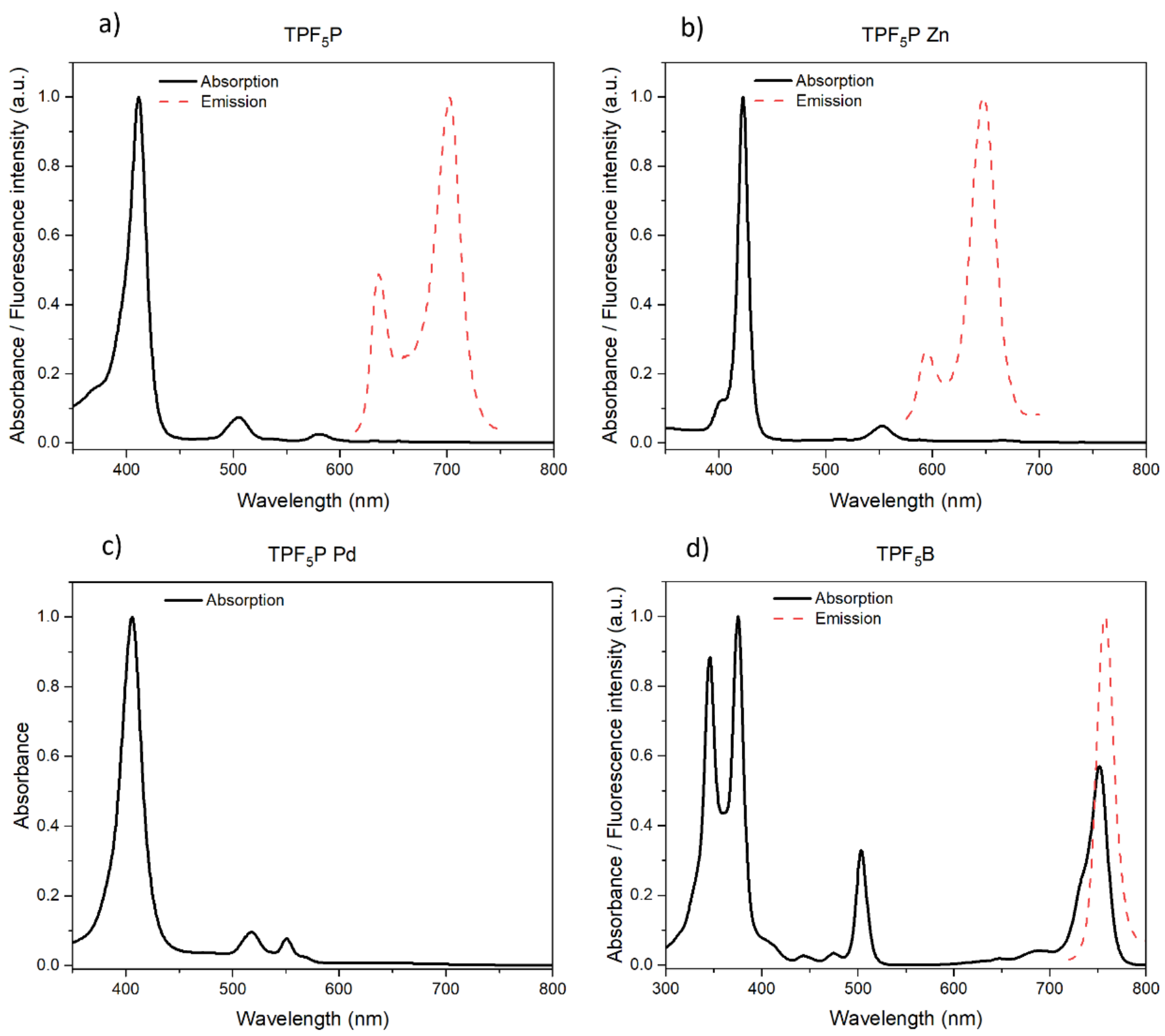

Figure S5. Normalized electronic absorption and emission spectra of synthesized photosensitizers (a) TPF_5P, (b) TPF_5P Zn, (c) TPF_5P Pd, and (d) TPF_5B, recorded in DMSO.

Quantum yield of photodecomposition

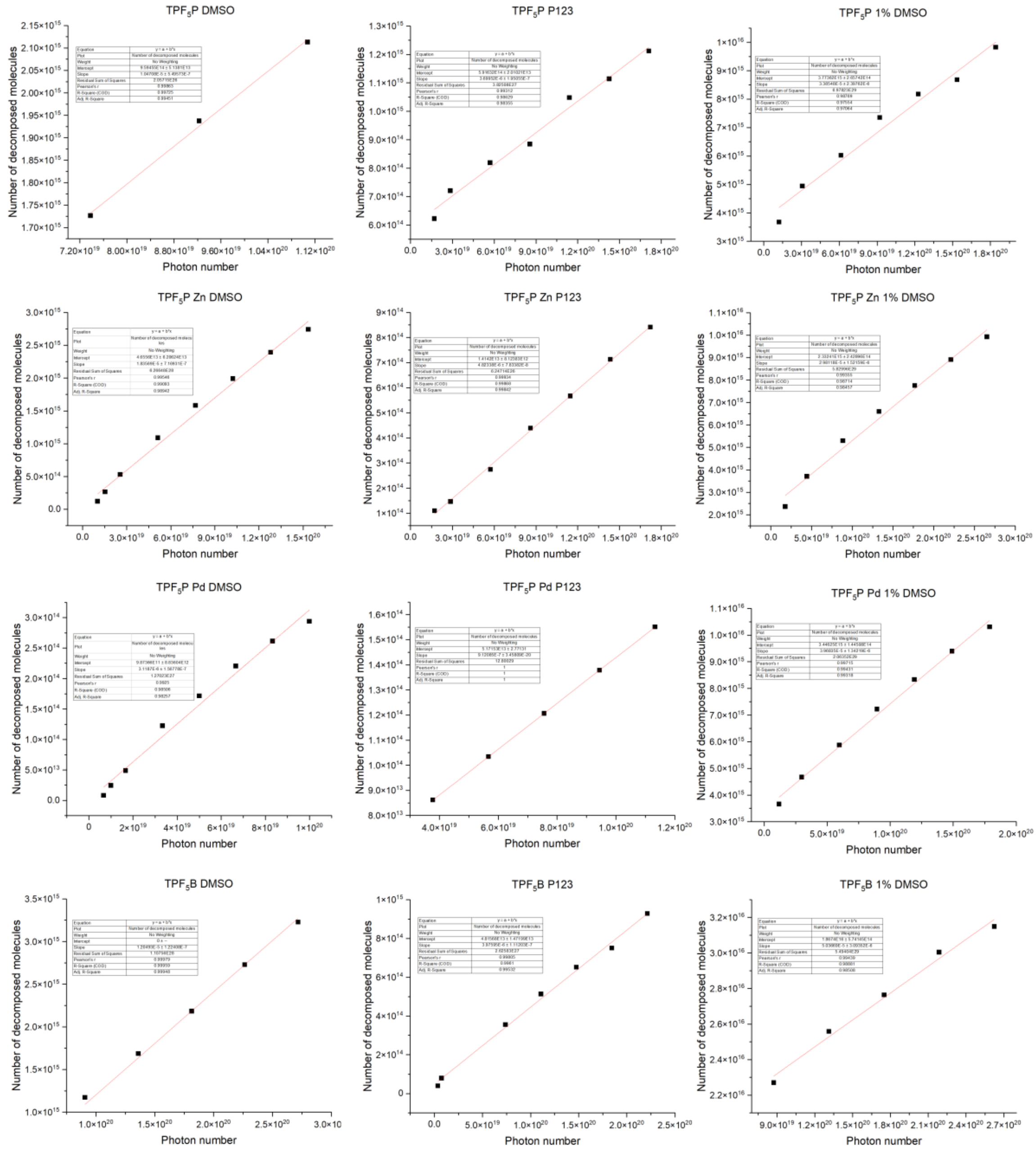

Figure S6. Photodegradation kinetics (molecular decay per photon). Porphyrins were irradiated with blue light (420 ± 20 nm) and bacteriochlorin with NIR light (750 ± 5 nm) at a light dose equal to 1 J/cm^2 per 1 minute in three formulations: DMSO, Pluronic P123, and 1% DMSO in PBS.

Triplet-state lifetime measurements

Triplet-state lifetimes were determined from transient absorption decay traces recorded after laser excitation at 355 nm under air- and argon-saturated conditions.

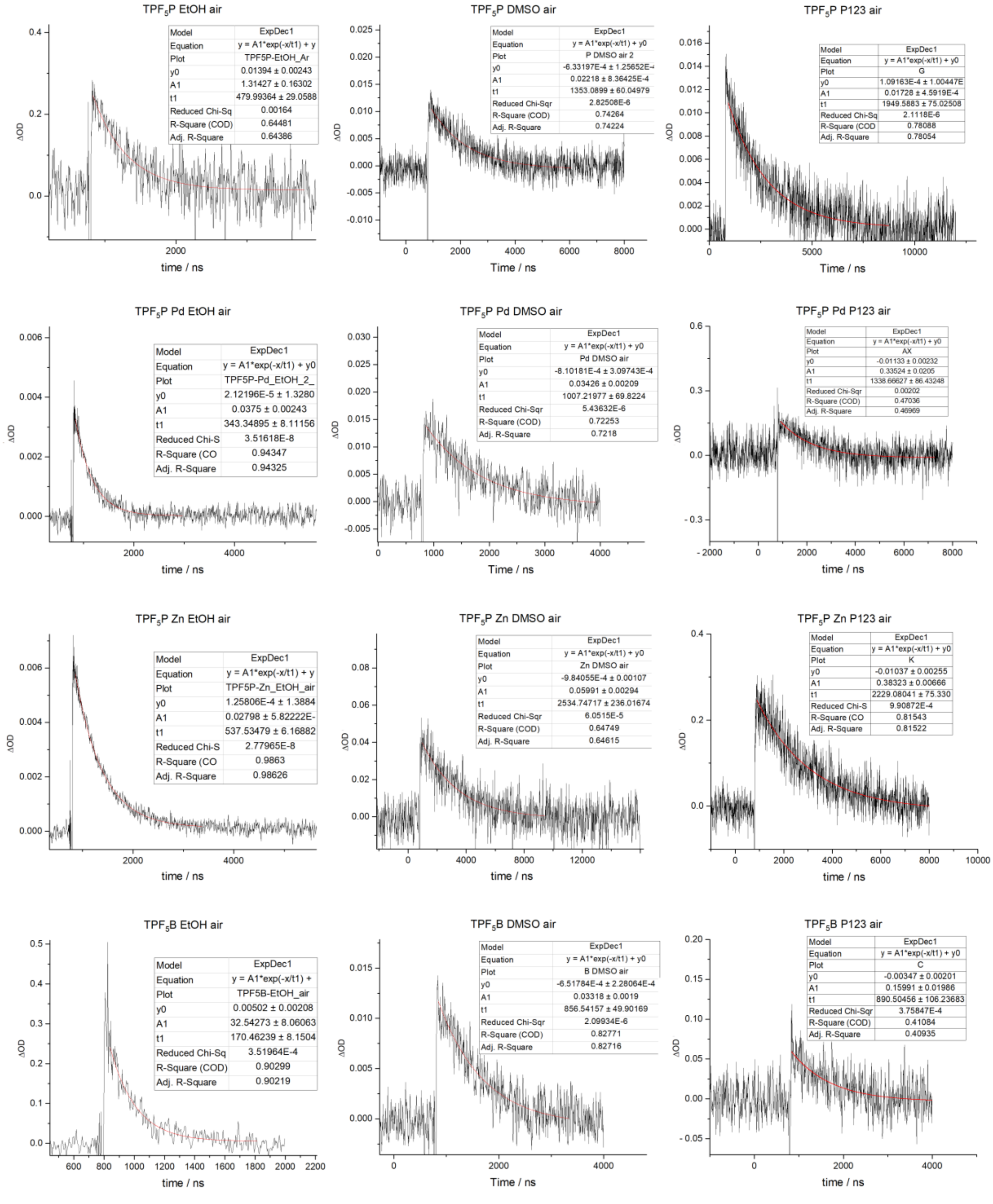

Figure S7. Transient absorption decay traces used to determine triplet-state lifetimes of TPF_5P, TPF_5P Pd, TPF_5P Zn, and TPF_5B in air-saturated ethanol, DMSO, and Pluronic P123 micelles in PBS. Data were obtained by laser flash photolysis at 20 °C with excitation at 355 nm.

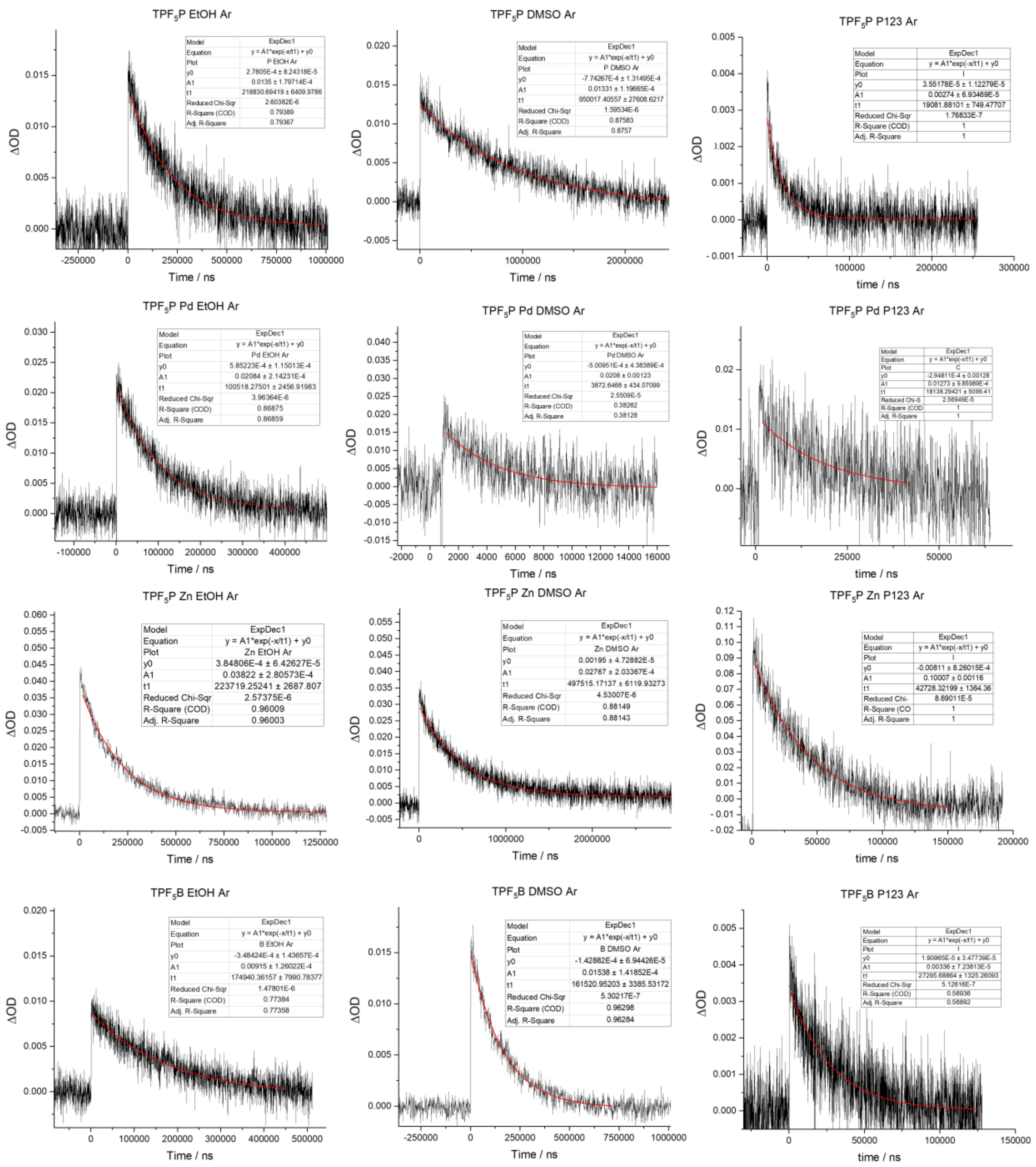

Figure S8. Transient absorption decay traces used to determine triplet-state lifetimes of TPF_5P, TPF_5P Pd, TPF_5P Zn, and TPF_5B in argon-saturated ethanol, DMSO, and Pluronic P123 micelles in PBS. Data were obtained by laser flash photolysis at 20 °C with excitation at 355 nm.

Reactive oxygen species detection.

ROS generation was evaluated using two complementary approaches: direct detection of singlet oxygen phosphorescence and complementary probe-based assays were used to compare ROS generation under defined irradiation and formulation conditions. DMA was used as a singlet oxygen-sensitive probe, DHE as a superoxide-related ROS probe, and APF as a hydroxyl-radical/hROS probe.

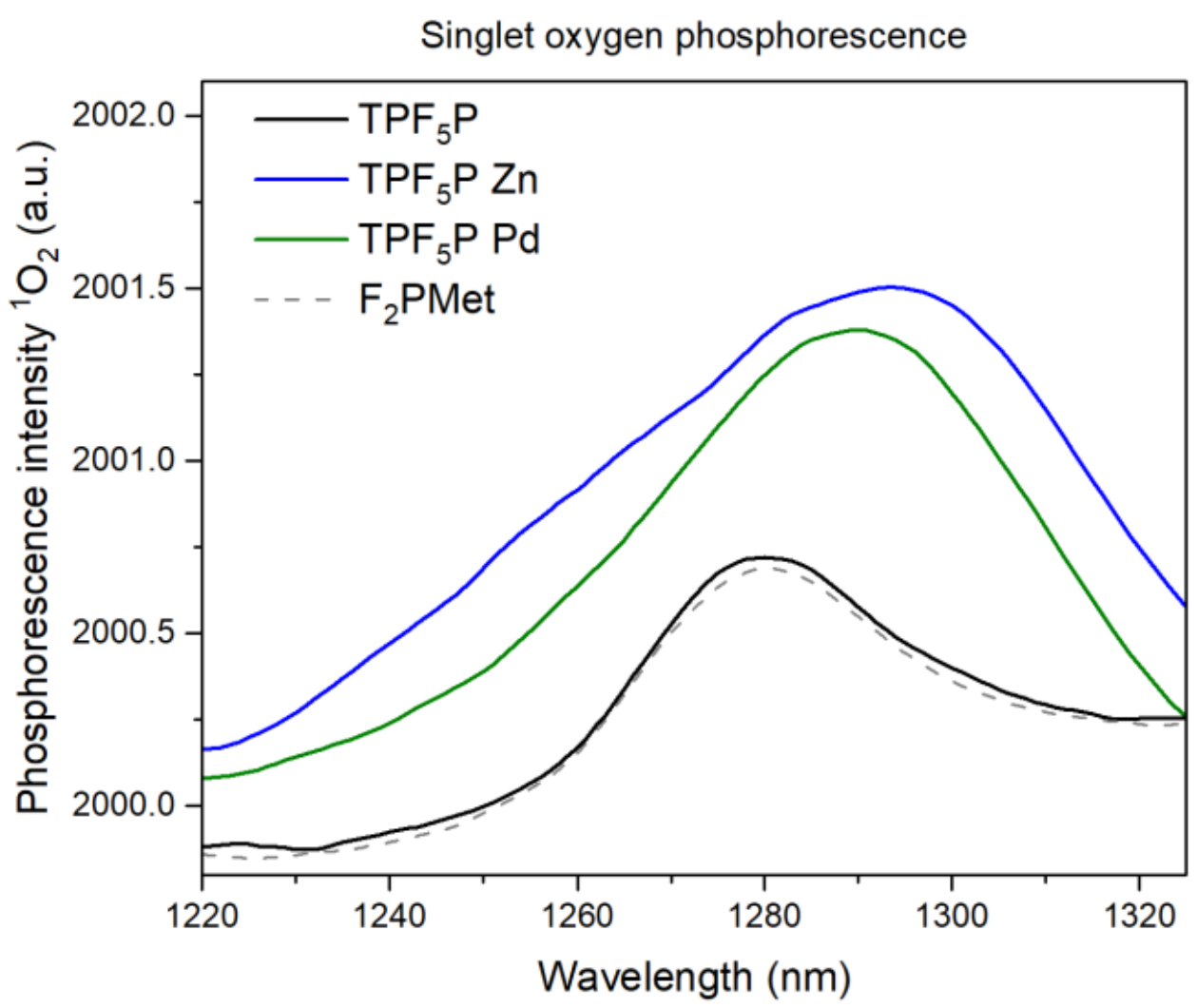

Figure S9. Direct detection of singlet oxygen phosphorescence generated by porphyrin photosensitizers in ethanol. Phosphorescence spectra were recorded for TPF_5P, TPF_5P Zn, TPF_5P Pd, and the F_2PMet reference under matched absorbance conditions using excitation at 412 nm.

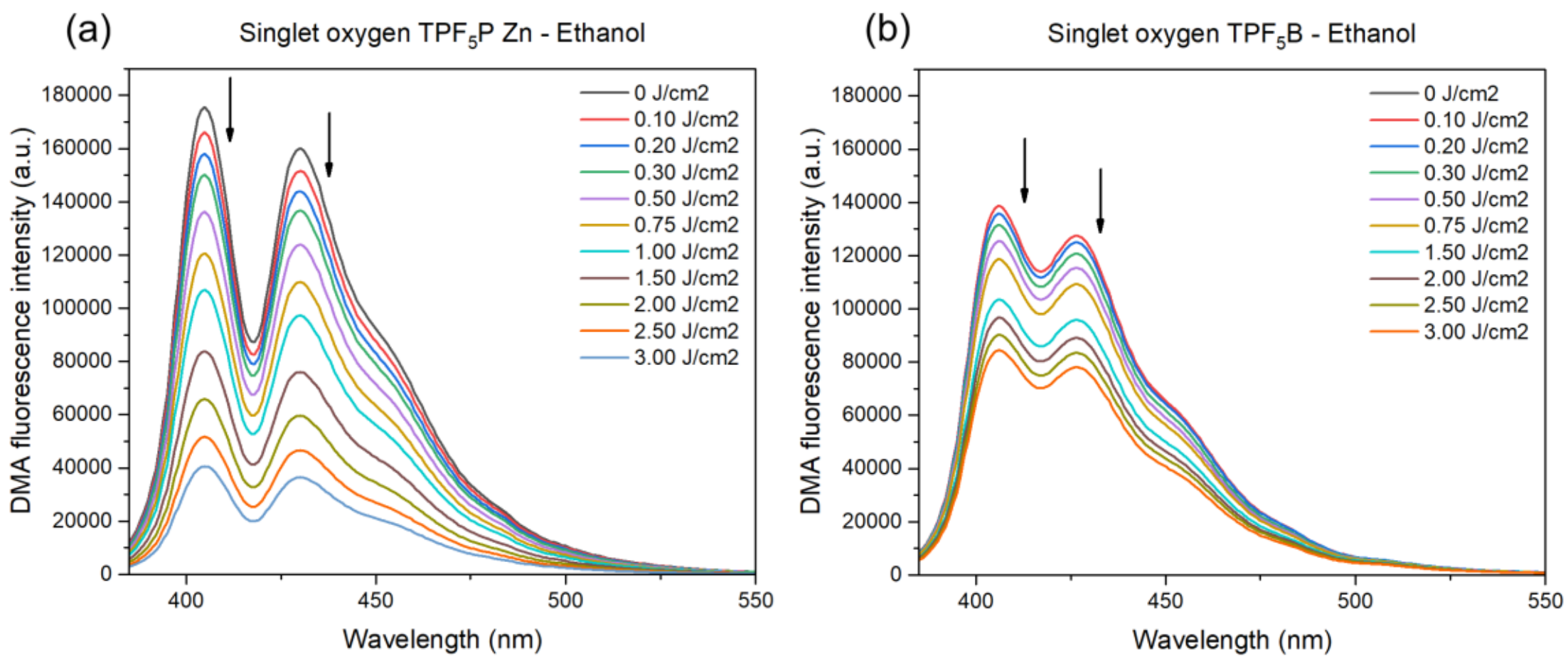

Figure S10. Representative DMA emission spectra recorded during irradiation are shown for the two compounds exhibiting the highest and the lowest singlet oxygen generation quantum yields respectively: (a) TPF_5P Zn under blue light (420 ± 20 nm) and (b) TPF_5B under NIR light (750 ± 5 nm). The progressive decrease in DMA fluorescence reflects singlet oxygen production.

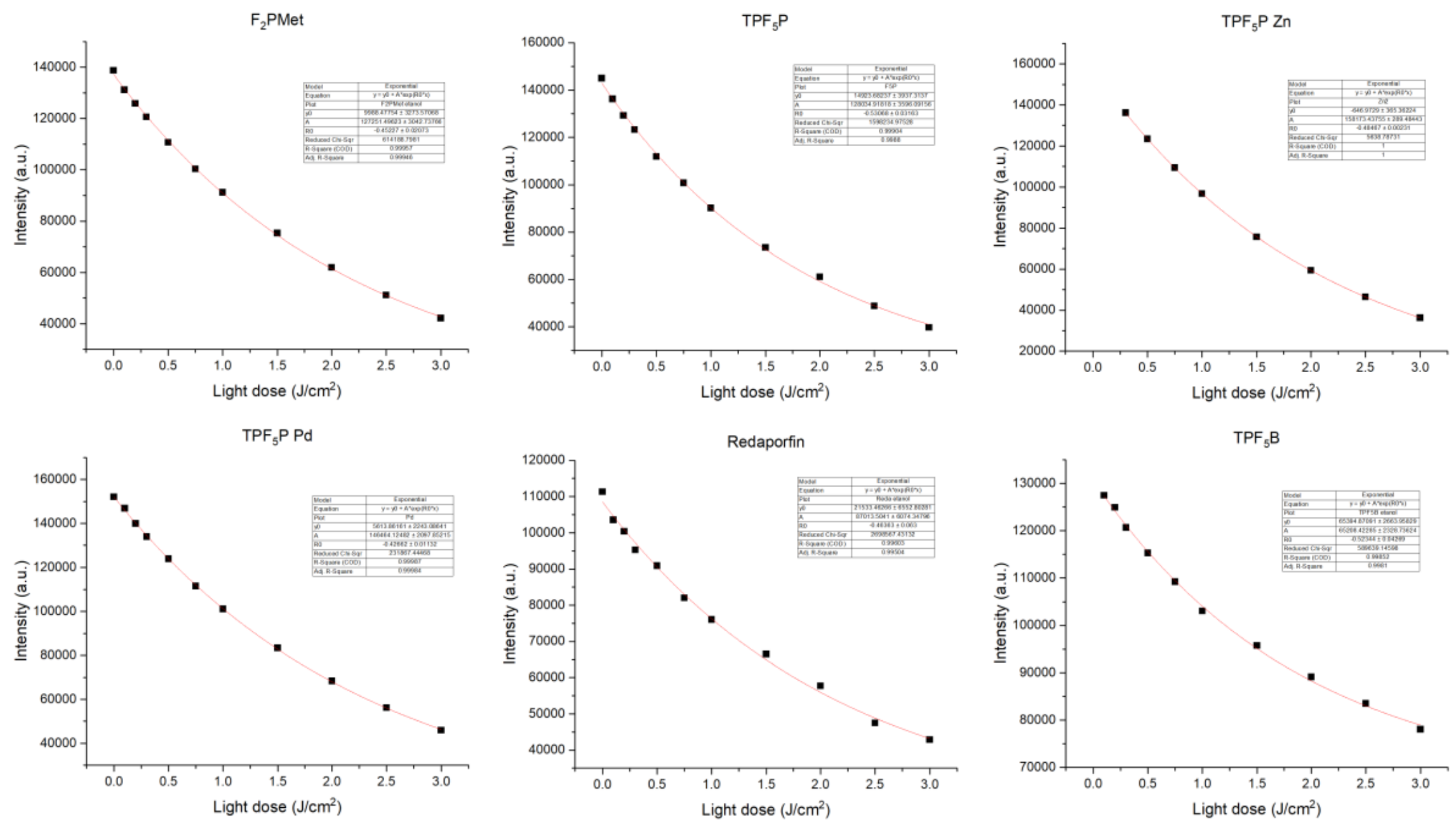

Figure S11. DMA fluorescence decay curves used for comparative singlet oxygen generation analysis in ethanol. F_2PMet and redaporfin (F_2BMet) used as reference photosensitizers for porphyrin and bacteriochlorin measurements, respectively.

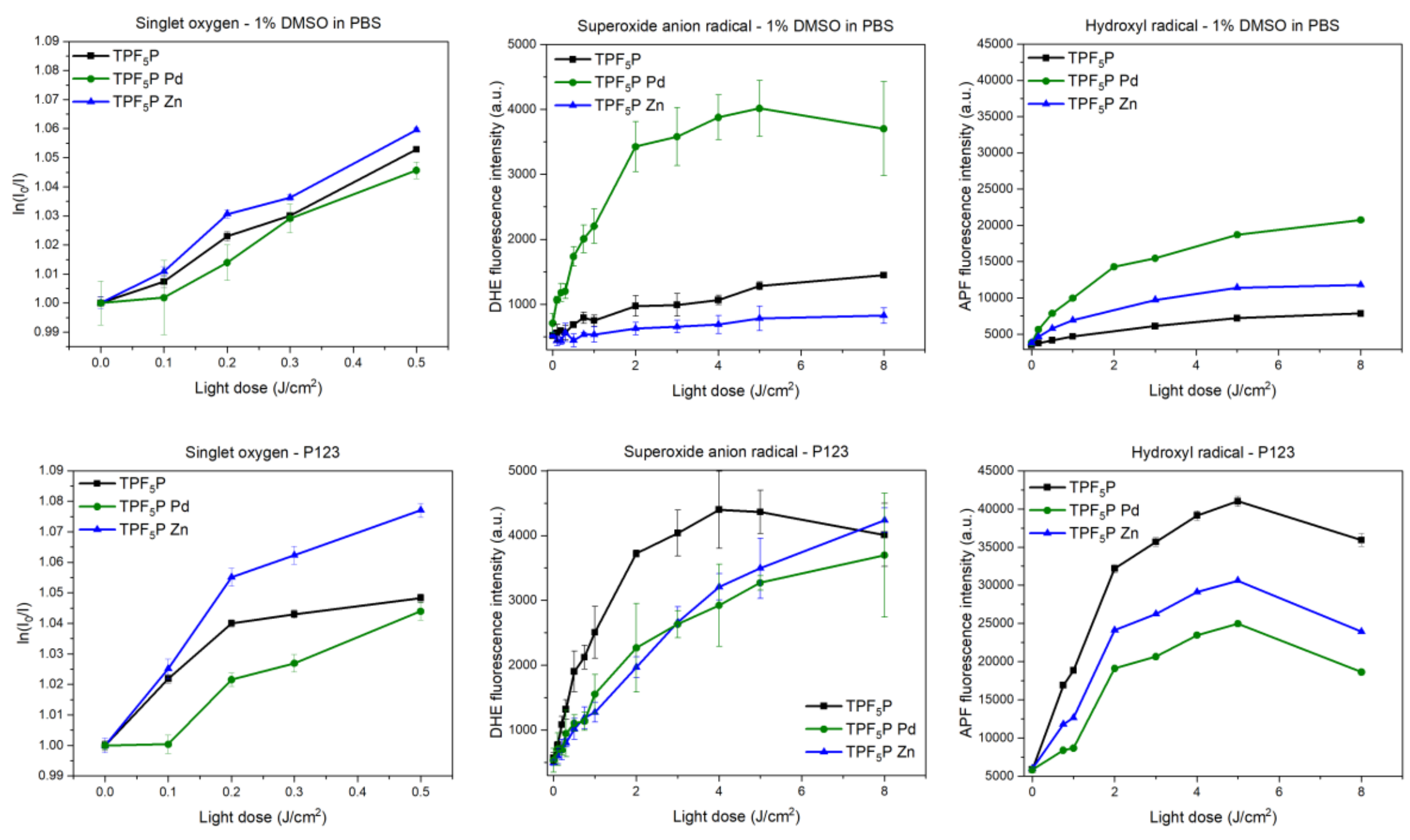

Figure S12. Comparative ROS readouts for porphyrins TPF_5P, TPF_5P Pd, and TPF_5P Zn in 1% DMSO in PBS and Pluronic P123 micelles. DMA was used as a singlet oxygen-sensitive probe, DHE as a superoxide-related ROS probe, and APF as a hydroxyl-radical/hROS probe.

Influence of DMSO on ROS generation

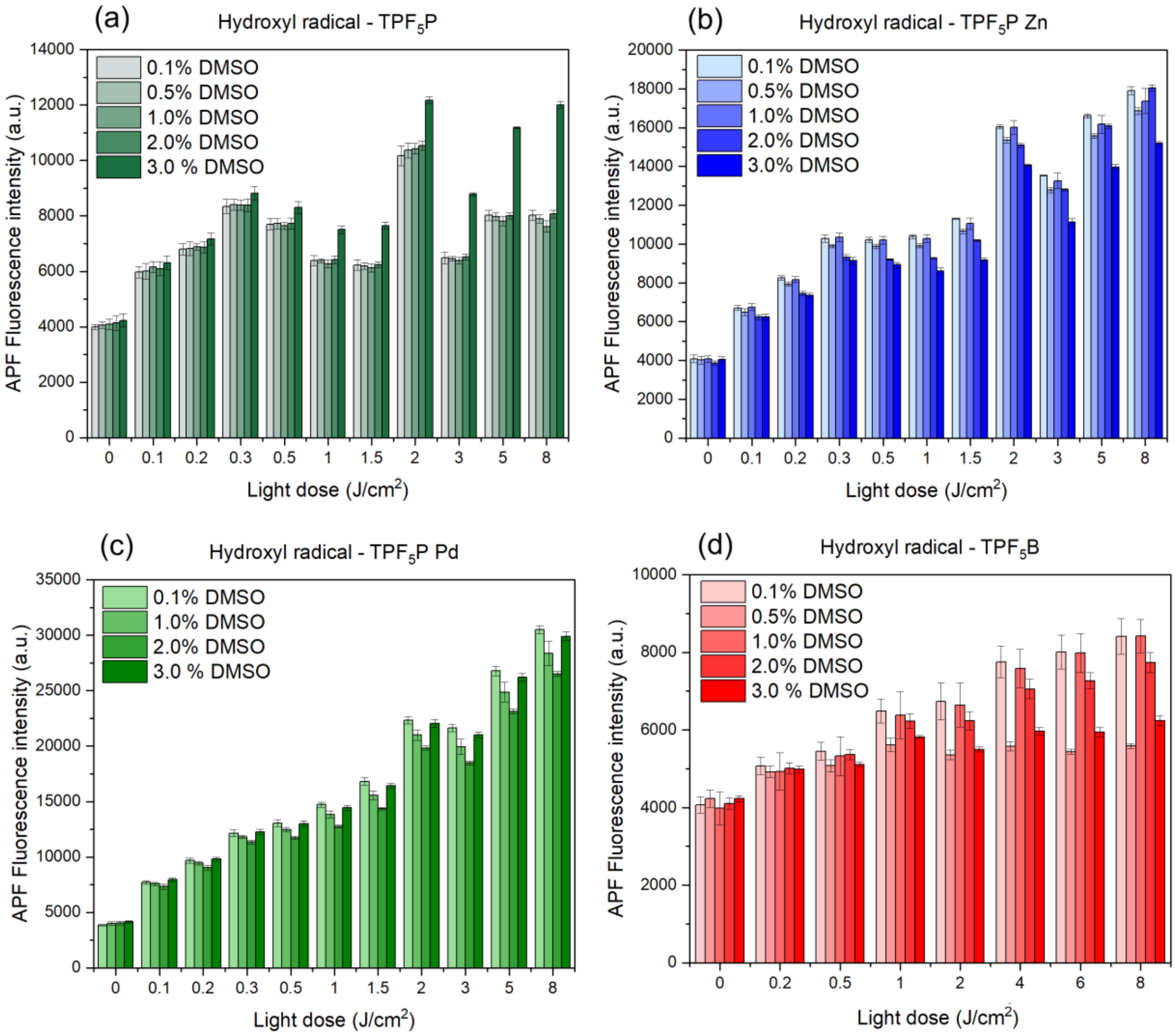

Figure S13. Influence of DMSO concentration (0.1–3%) on the APF-based hydroxyl-radical-related response. Samples were irradiated with blue light (420 ± 20 nm) for porphyrins (a–c) and NIR light (750 ± 5 nm) for TPF_5B (d) at a photosensitizer concentration of 5 µM and APF concentration of 10 µM.

Cellular uptake

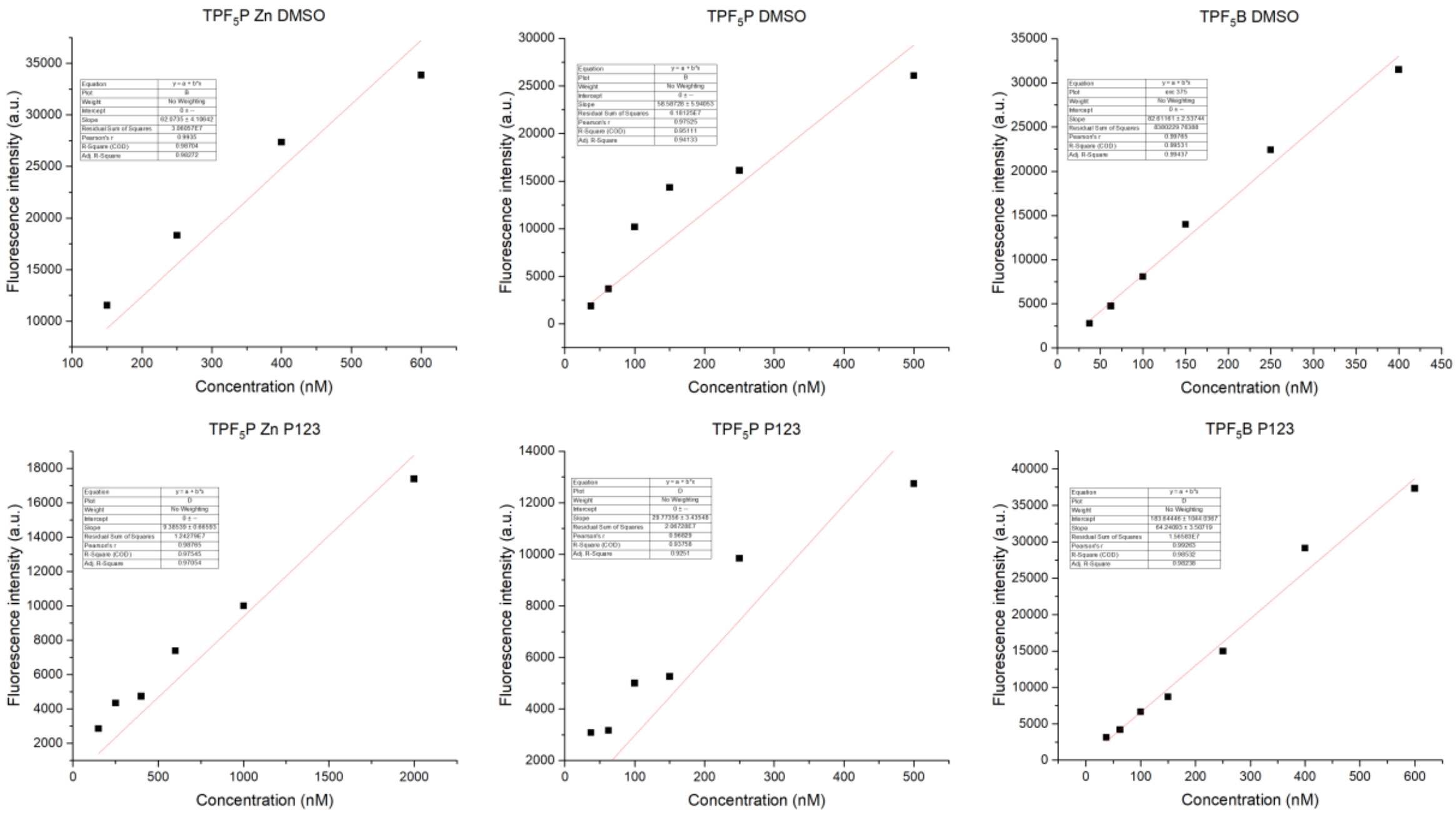

Figure S14. Calibration curves for the assessment of cellular uptake were obtained in a medium composed of 30% (v/v) of 1% Triton X-100 and 70% (v/v) of a DMSO/ethanol mixture (1:3, v/v), containing the photosensitizer in the respective formulation.

Intracellular localization

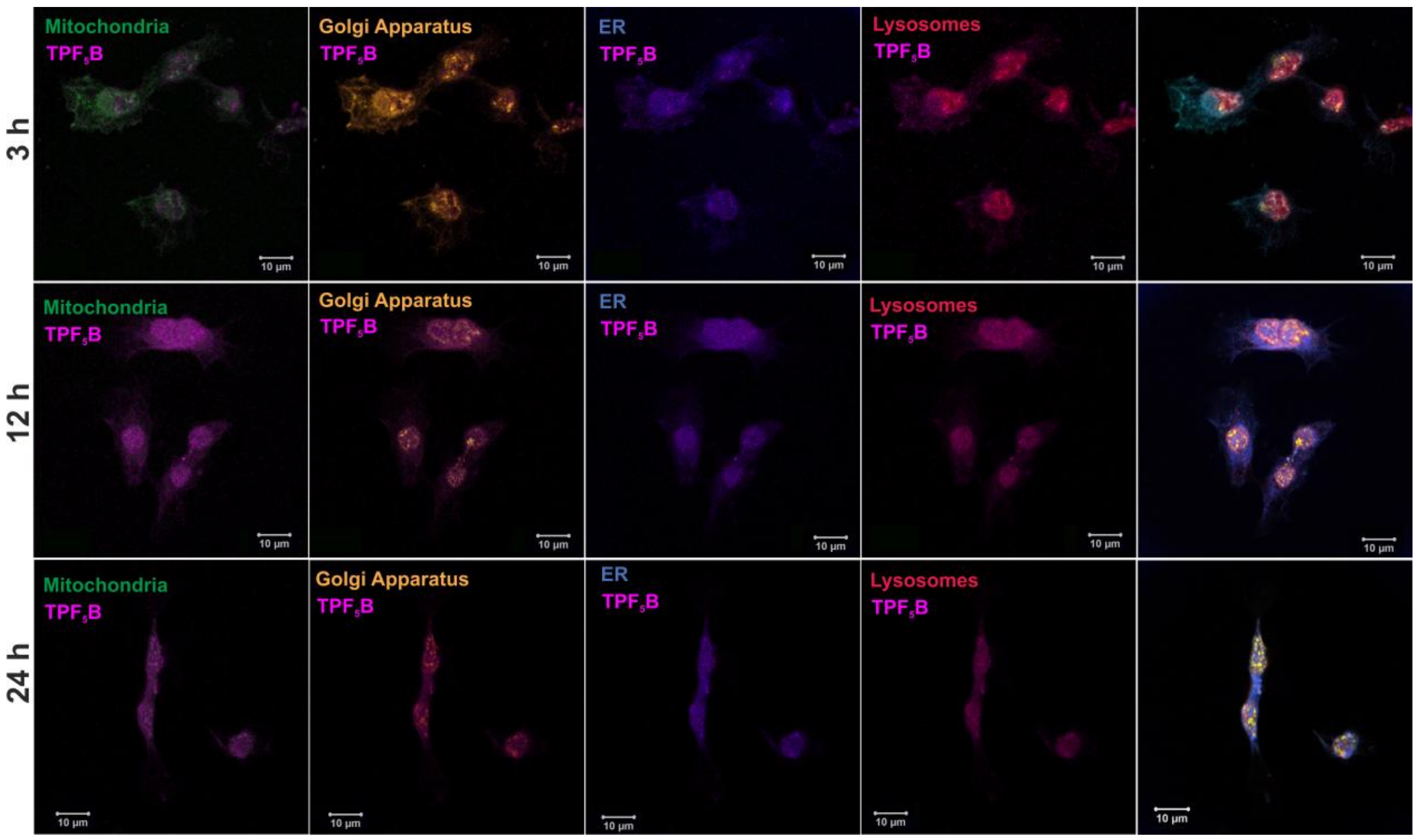

Figure S15. Fluorescence confocal imaging of TPF_5B dissolved in DMSO (final concentration in cell medium 1%) and different cell organelles - a) lysosomes (stained with α-LAMP-1), b) mitochondria (stained with α-MTCO1), c) Golgi apparatus (stained with α-GM130), d) endoplasmic reticulum (stained with $DiOC_3(6)$).

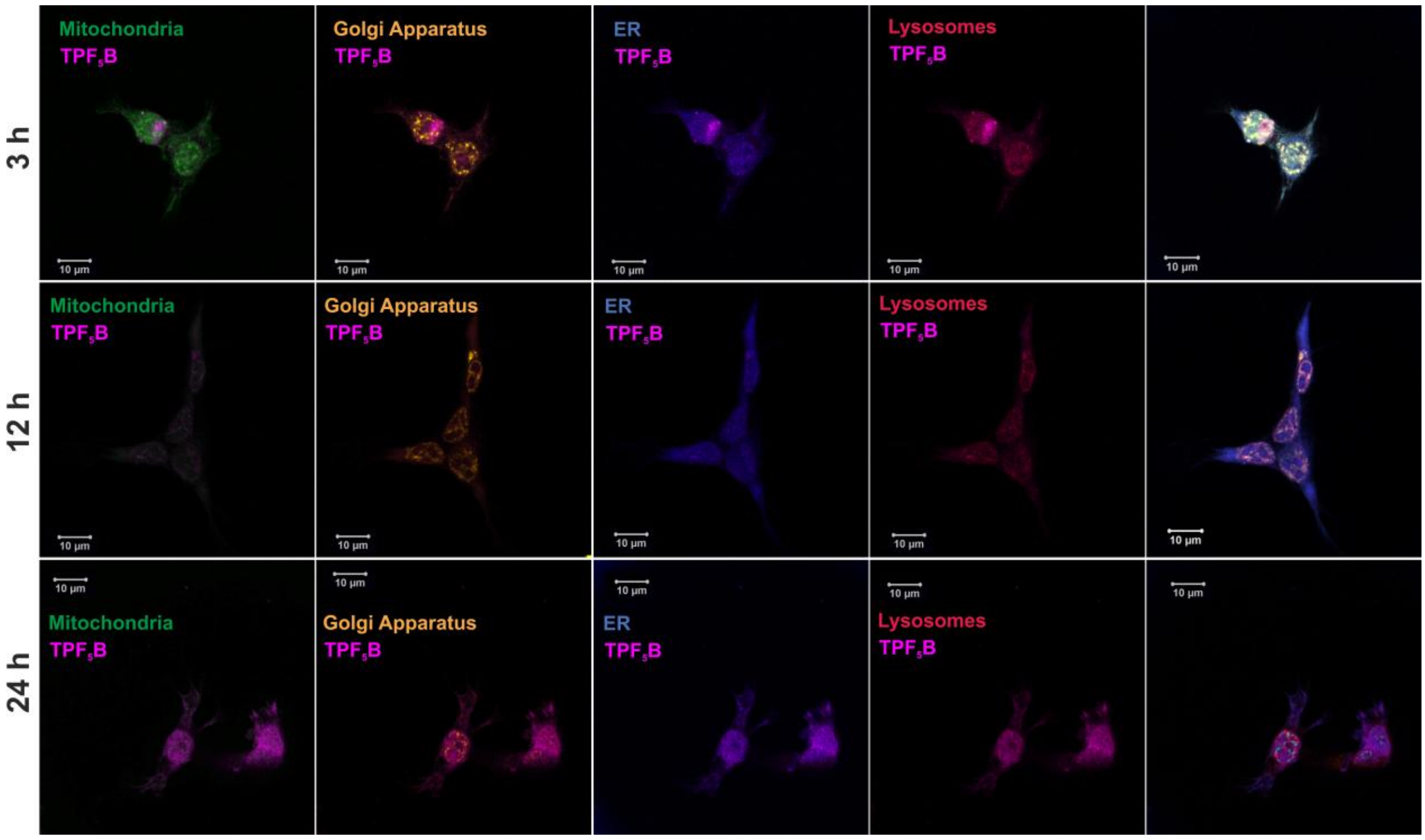

Figure S16. Fluorescence confocal imaging of TPF_5B encapsulated in Pluronic P123 micelles and different cell organelles - a) lysosomes (stained with α-LAMP-1), b) mitochondria (stained with α-MTCO1), c) Golgi apparatus (stained with α-GM130), d) endoplasmic reticulum (stained with $DiOC_3(6)$).

Confirmation of the photosensitizer structure

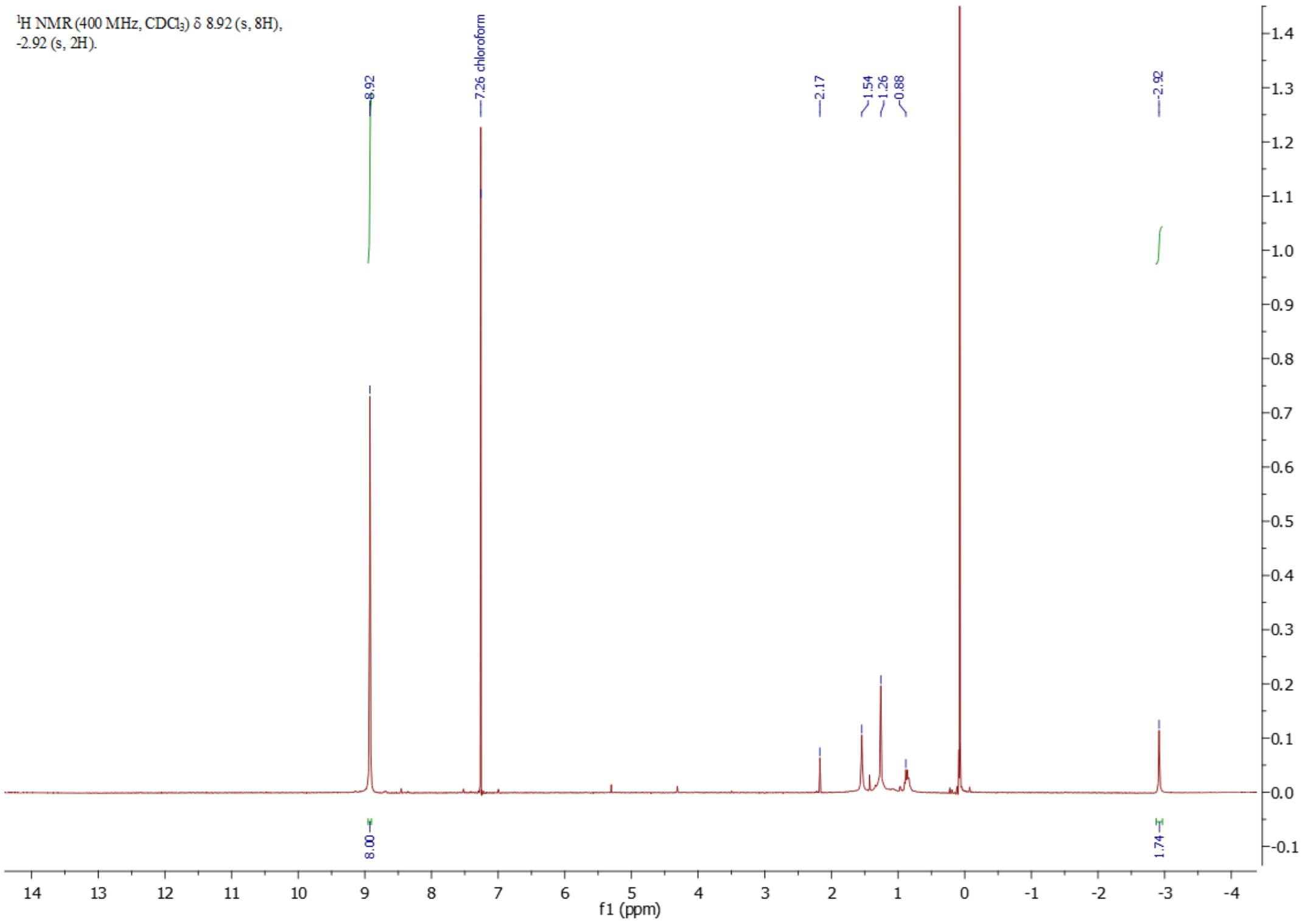

Figure S17. ^{1}H NMR spectrum of TPF$_5$P in $CDCl_3$-d. Signals at 2.17, 1.54, 1.26, 0.88 are common signals from solvents (acetone, water and *n*-hexane, respectively).

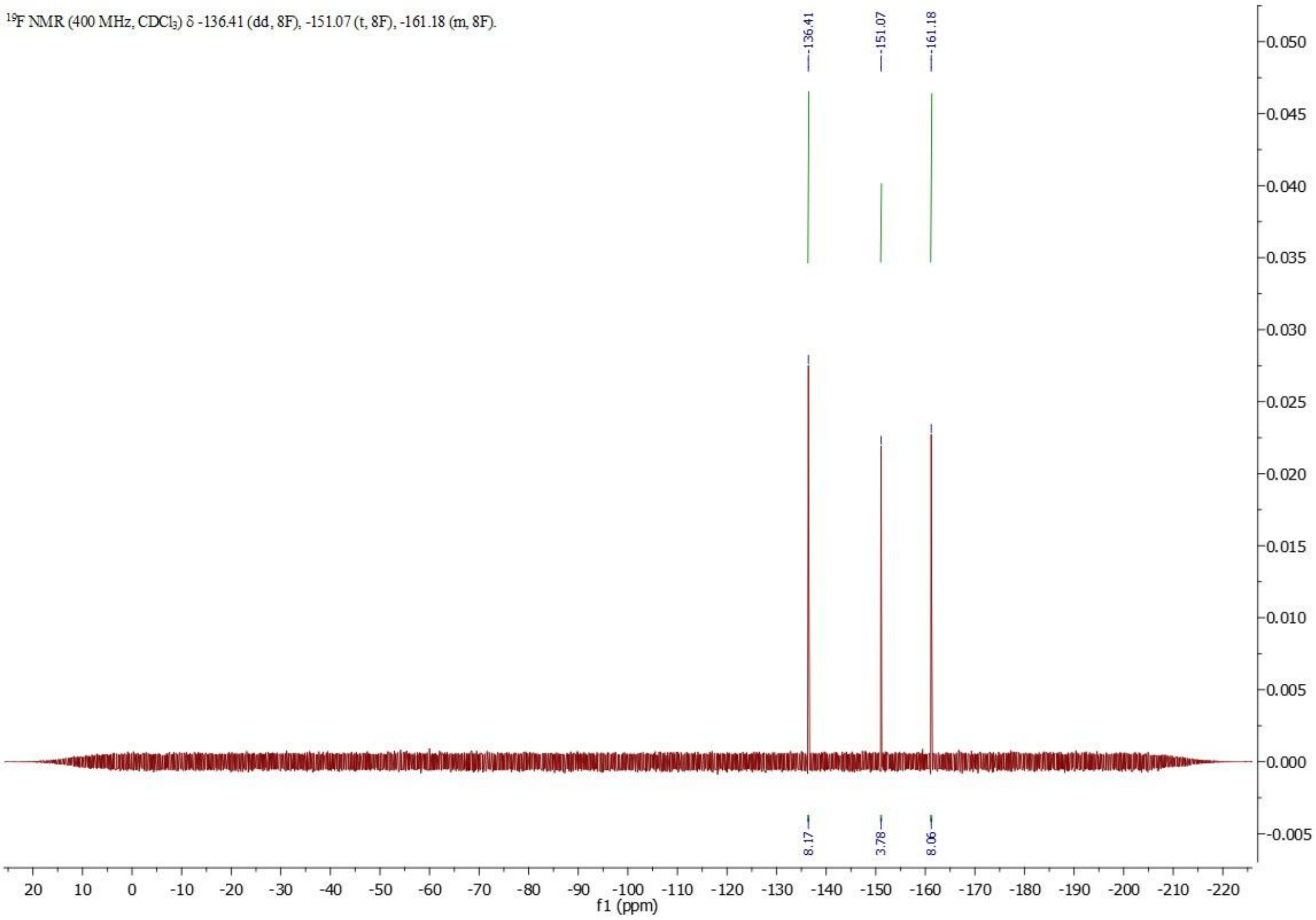

Figure S18. ^{19}F NMR spectrum of TPF$_5$P in $CDCl_3$-d.

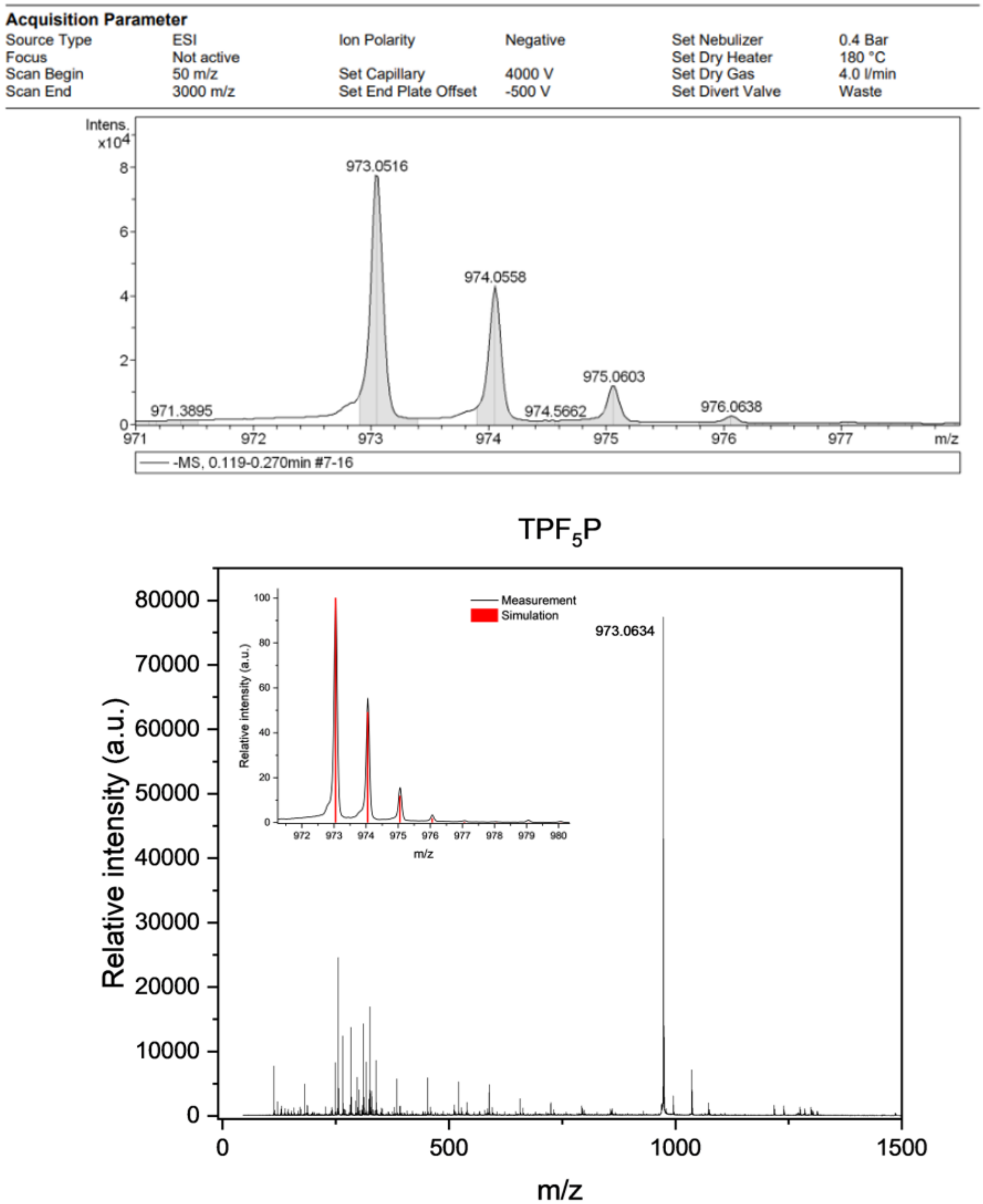

Figure S19. ESI- mass spectra obtained for TPF_5P (MW = 974.56), together with the isotope pattern simulated using ChemCalc.org.

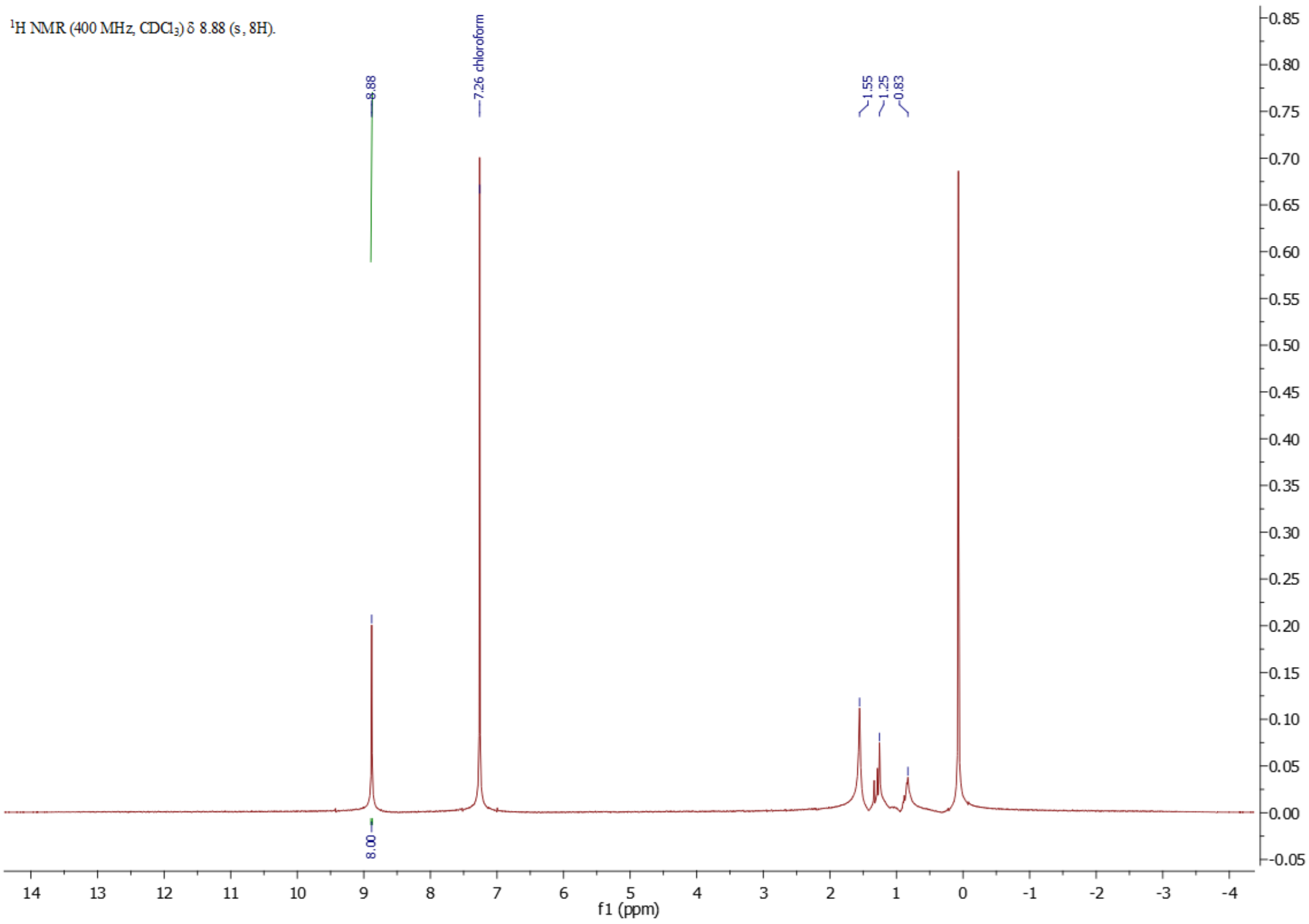

Figure S20. ^{1}H NMR spectrum of TPF$_5$P Zn in CDCl$_3$-d. Signals at 1.55 and 1.25, 0.83 are common signals from solvents (water and *n*-hexane, respectively).

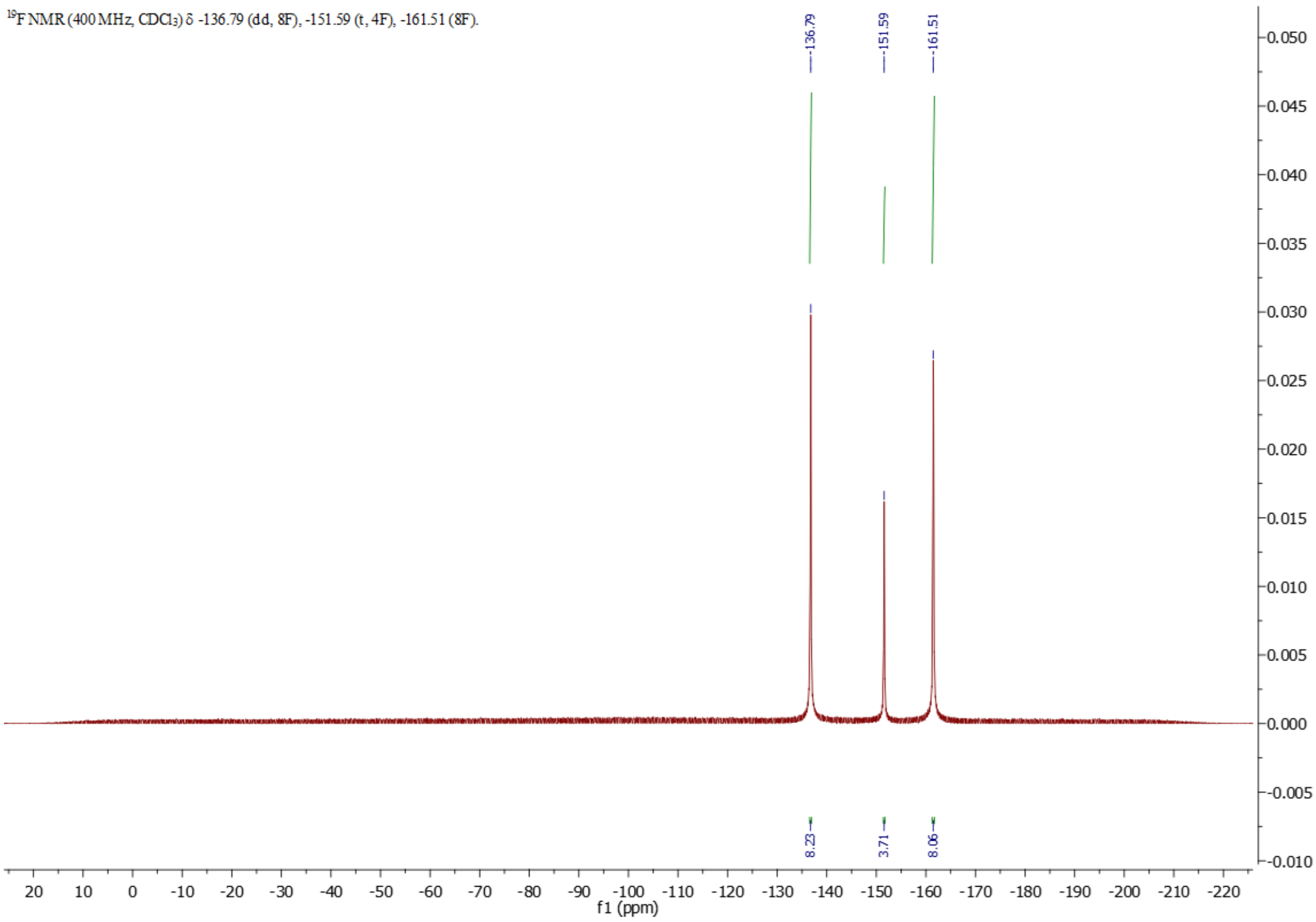

Figure S21. ^{19}F NMR spectrum of TPF$_5$P Zn in CDCl$_3$-d.

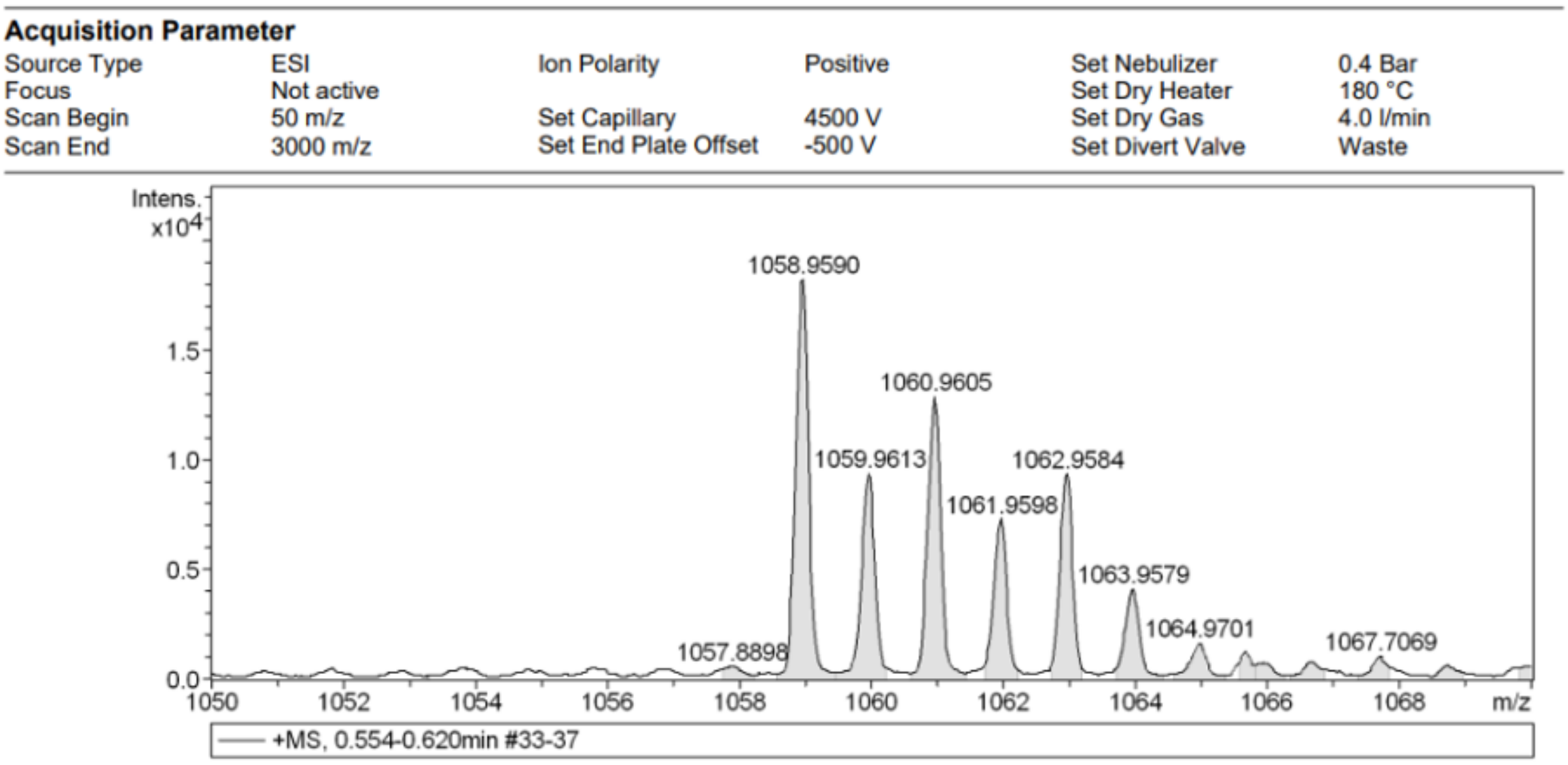

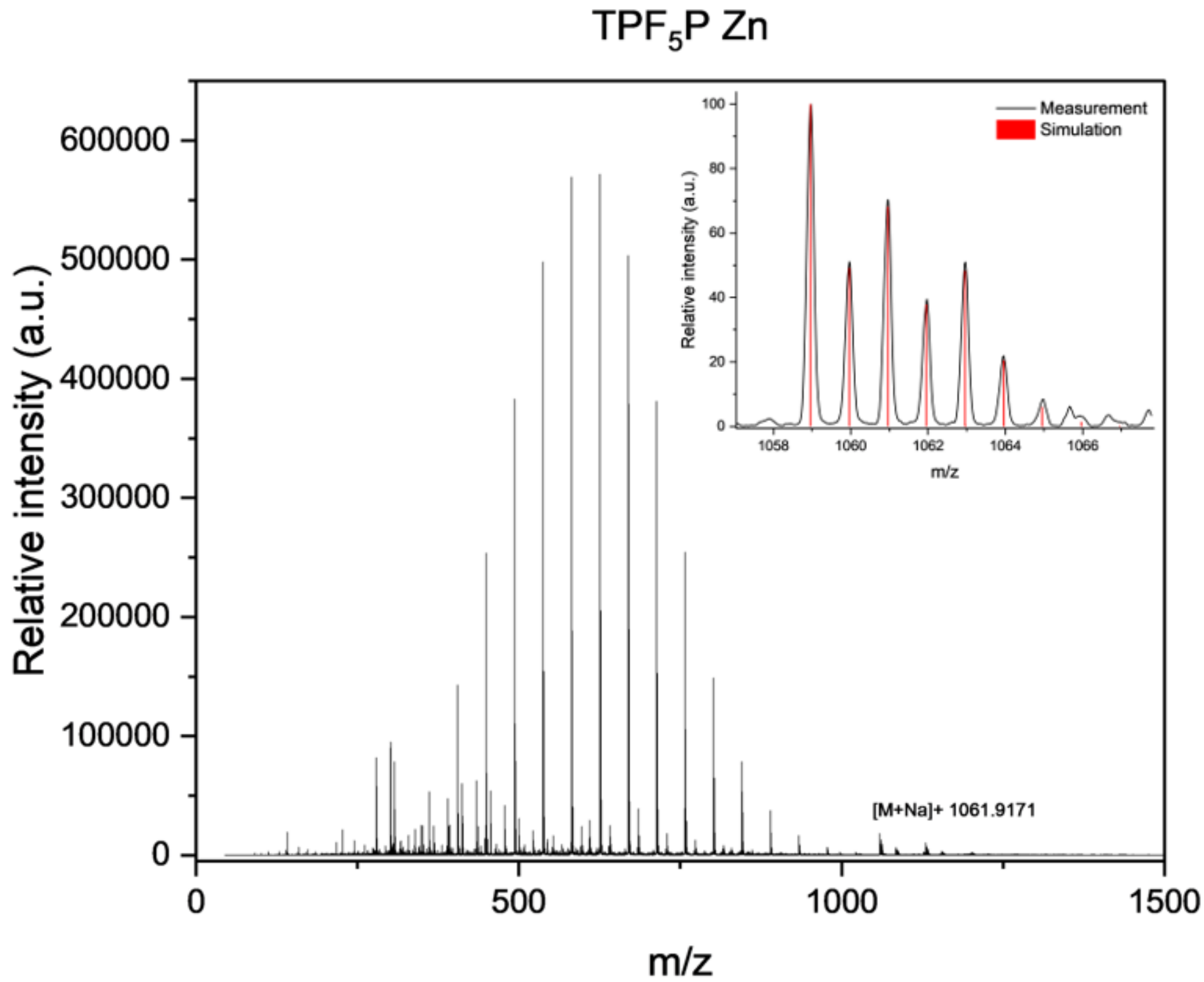

Figure S22. ESI+ mass spectra obtained for TPF_5P Zn (MW = 1037.92), together with the isotope pattern simulated using ChemCalc.org.

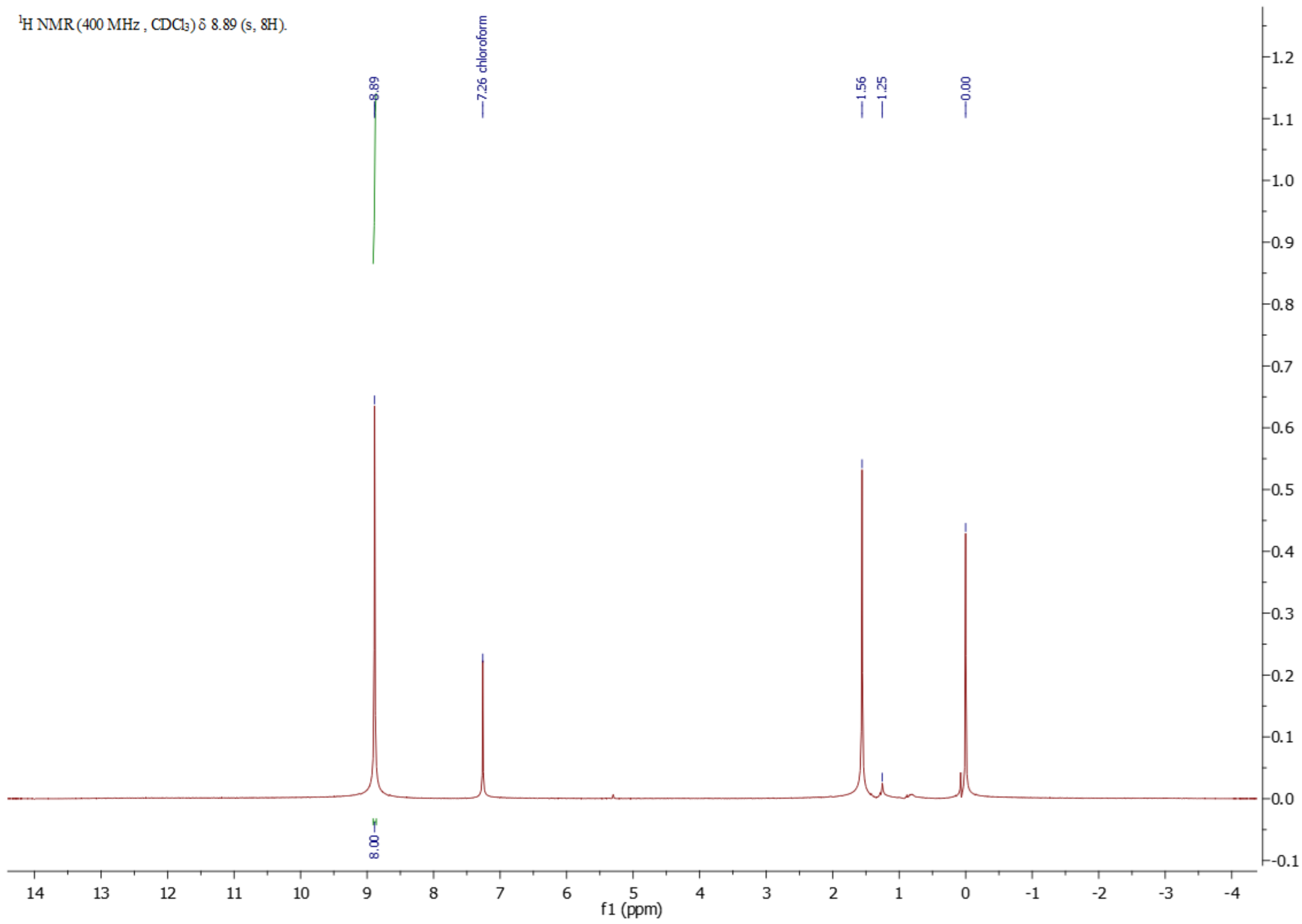

Figure S23. ^{1}H NMR spectrum of TPF_5P Pd in $CDCl_3$-d. Signals at 1.56 and 1.25 are common signals from solvents (water and *n*-hexane respectively).

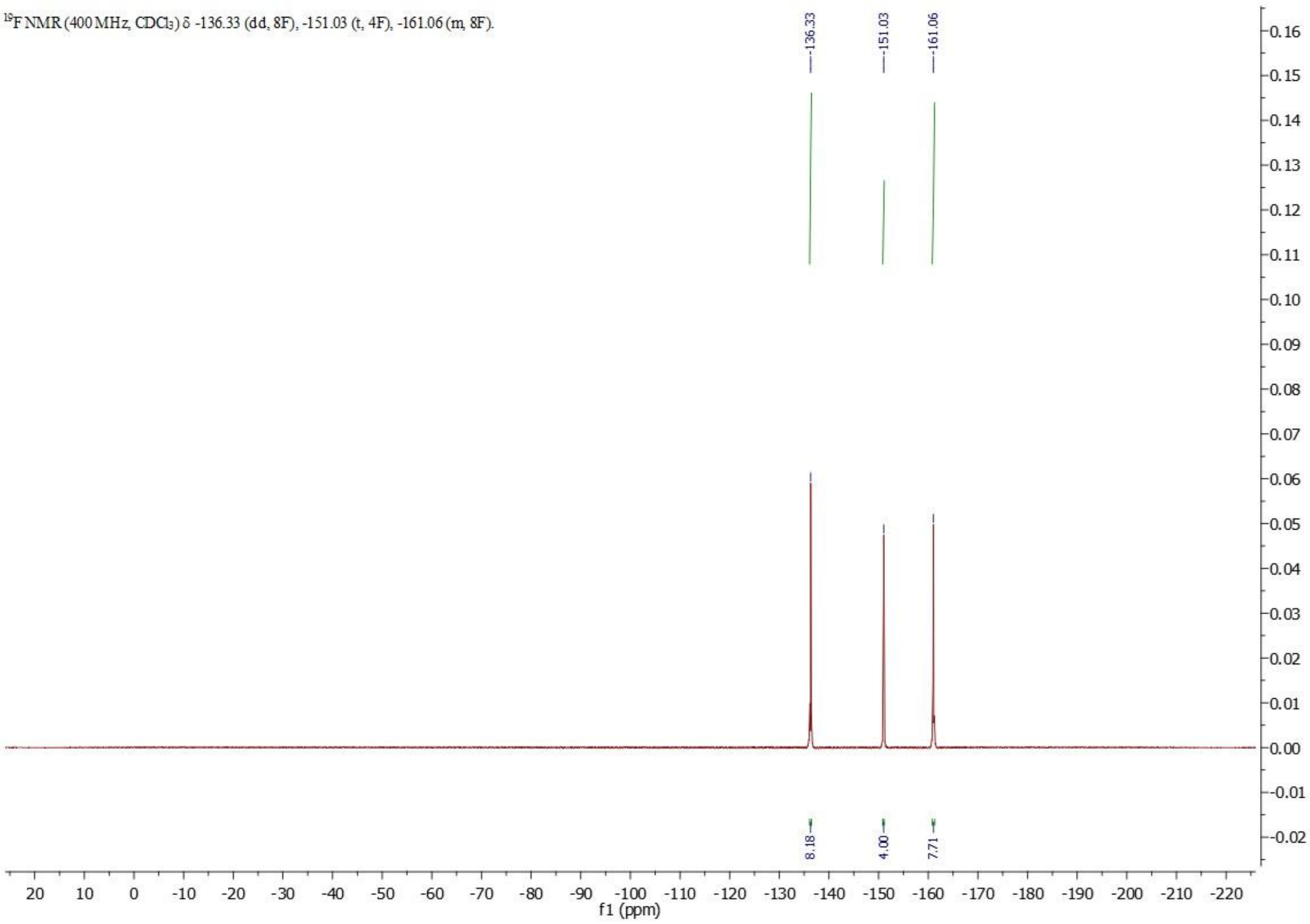

Figure S24. ^{19}F NMR spectrum of TPF_5P Pd in $CDCl_3$-d

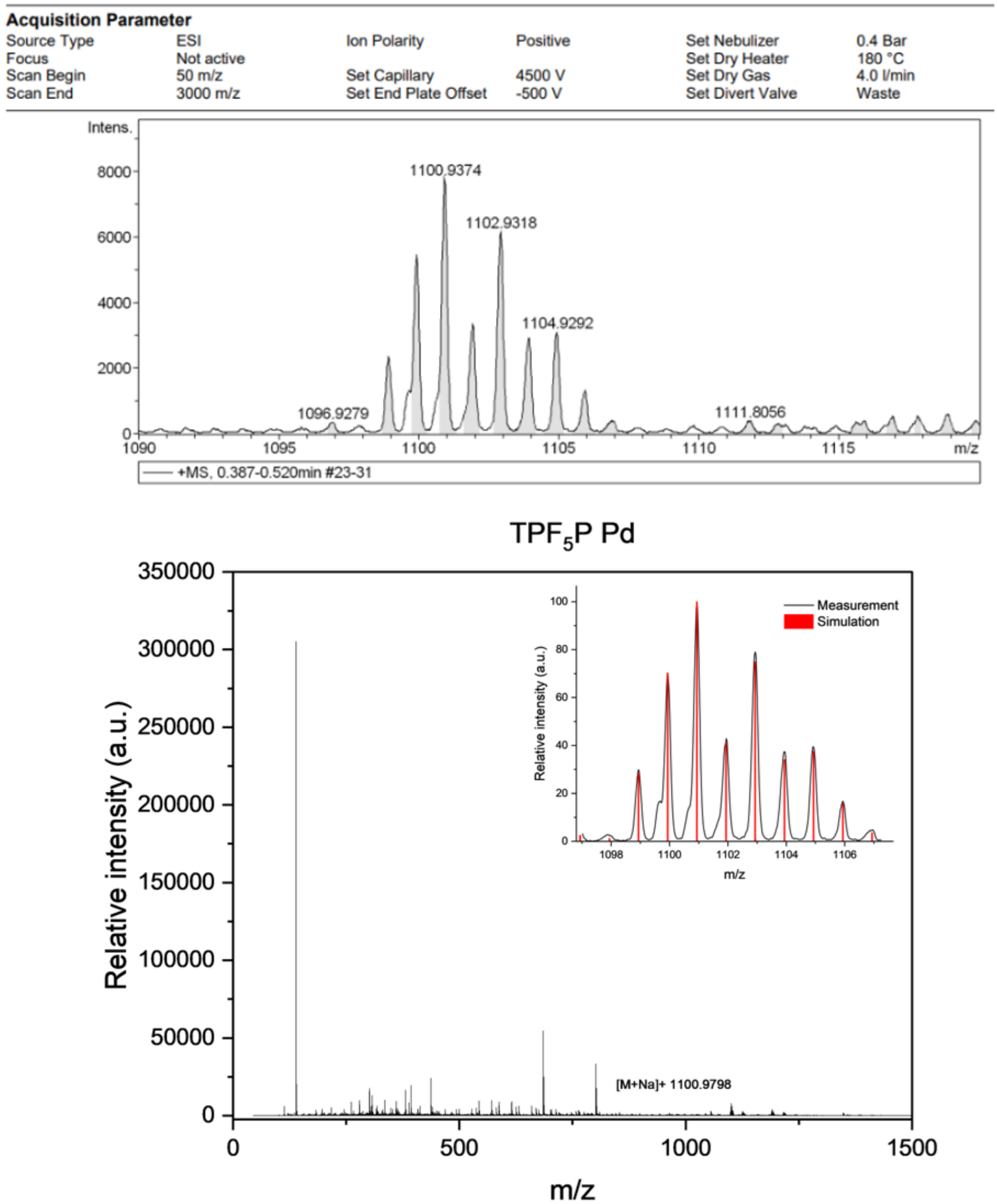

Figure S25. ESI+ mass spectra obtained for TPF_5P Pd (MW = 1078.96), together with the isotope pattern simulation using ChemCalc.org.

For TPF_5P Zn and TPF_5P Pd analyzed in positive ionization mode (ESI+), the observed molecular ion peak appears at an m/z value 23 units higher than the calculated molecular weight, consistent with the formation of the sodium adduct $[M + Na]^+$. Such adduct formation is commonly observed in soft ionization techniques, particularly electrospray ionization, due to the ubiquitous presence of trace sodium ions originating from solvents, glassware, or sample handling.

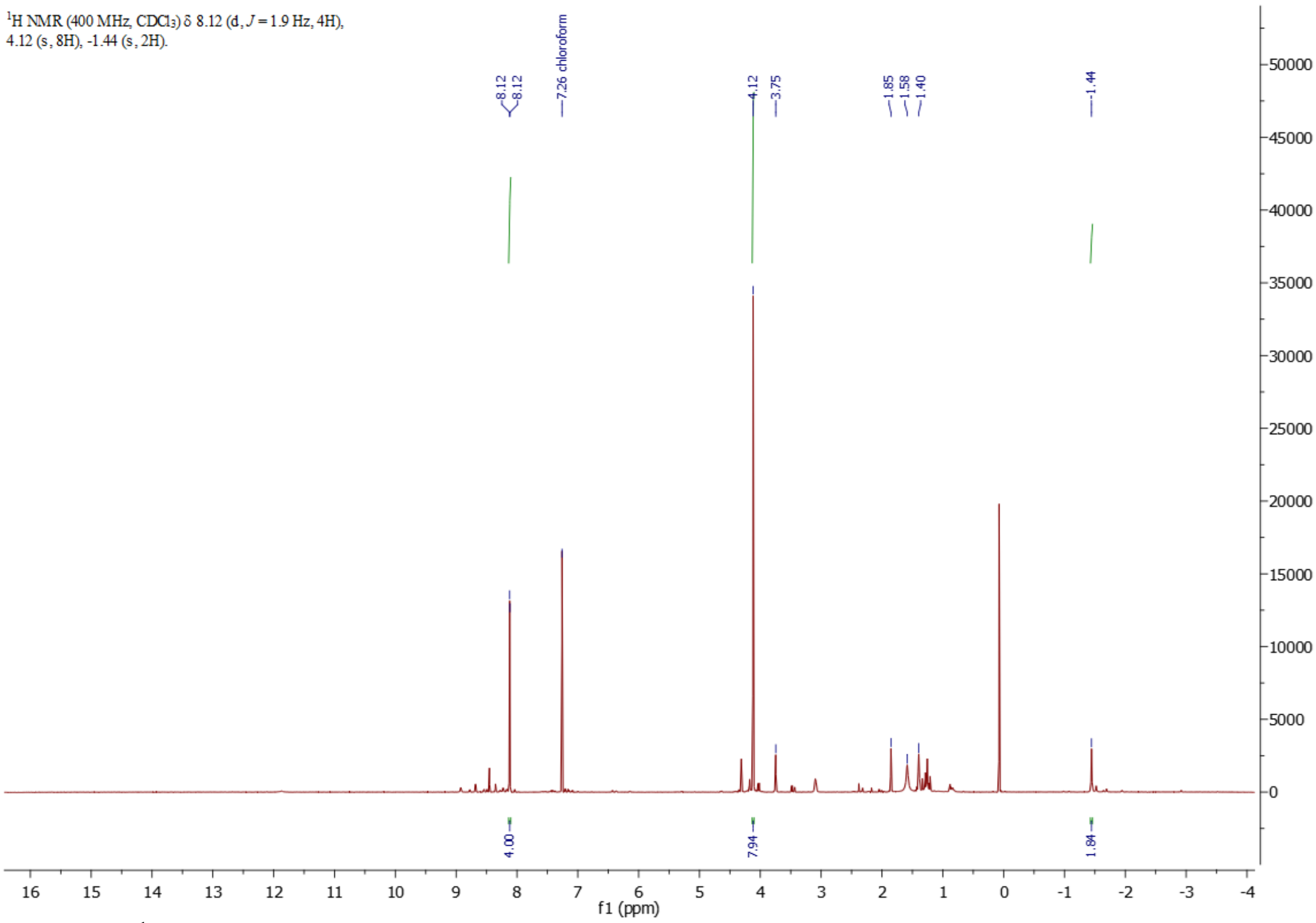

Figure S26. ^{1}H NMR spectrum of TPF_5B in $CDCl_3$-d. Signals at 1.58 and 3.75, 1.85, 1.40 are common signals from solvents (water and ethanol respectively).

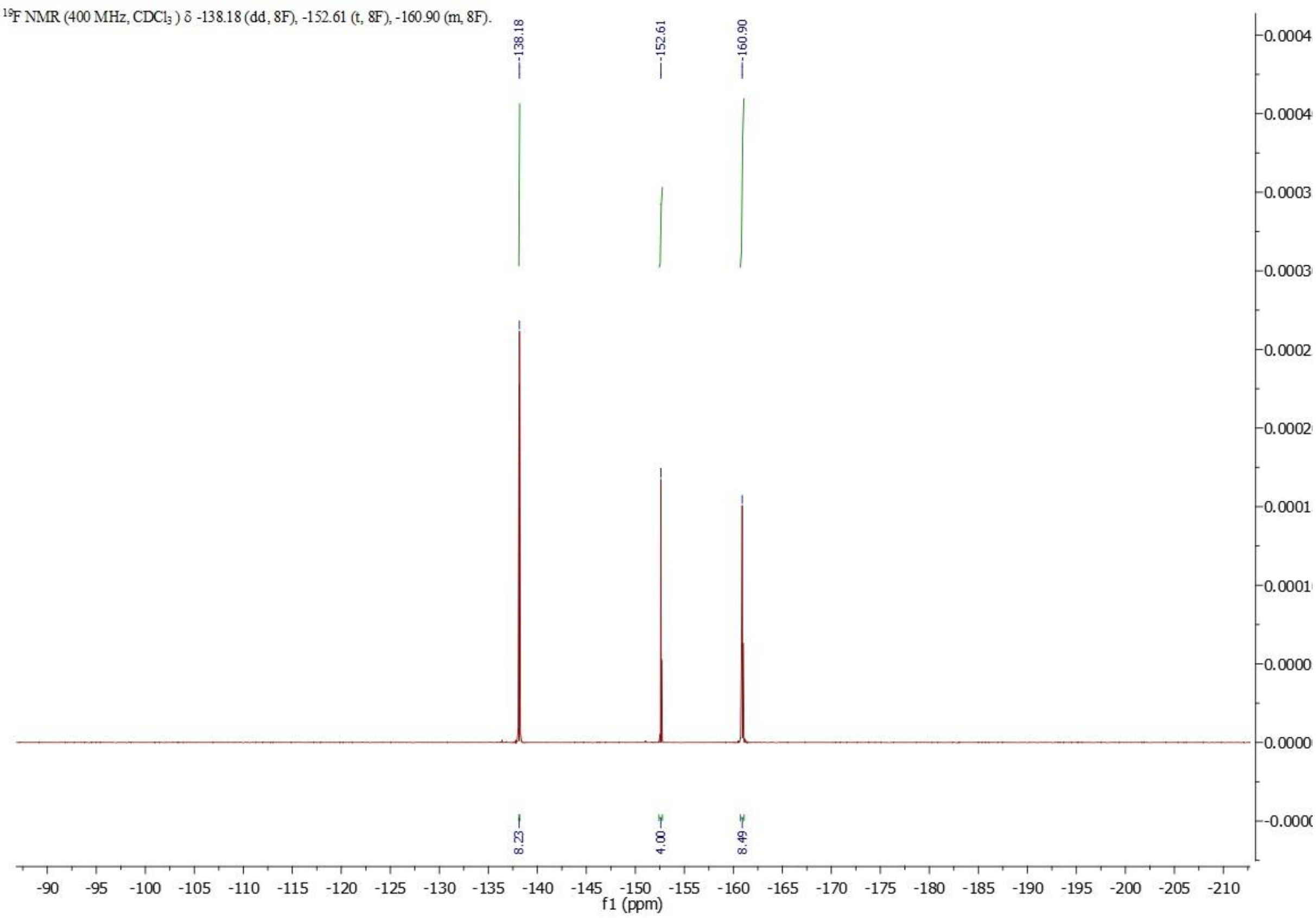

Figure S27. ^{19}F NMR spectrum of TPF_5B in $CDCl_3$-d.

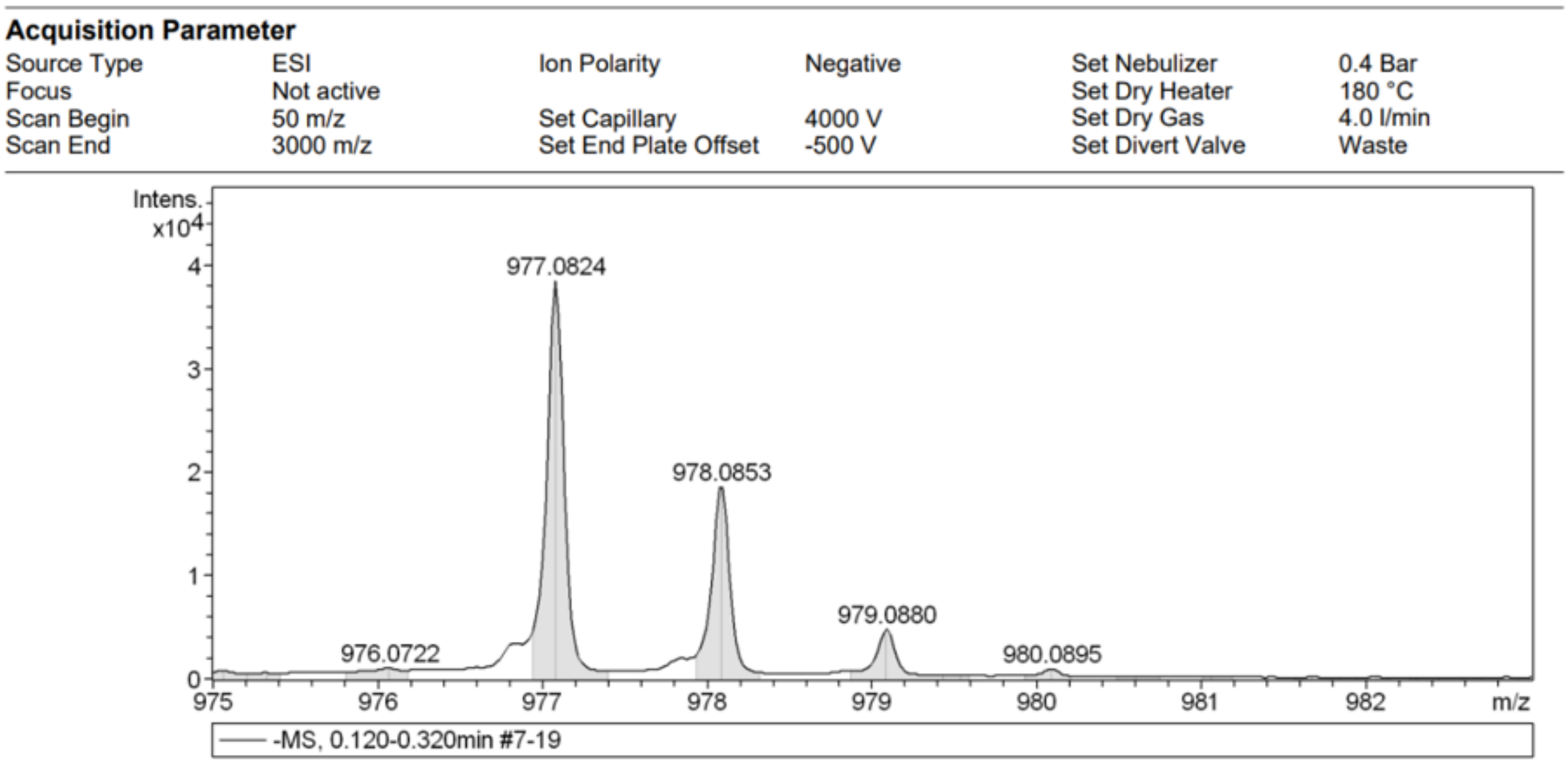

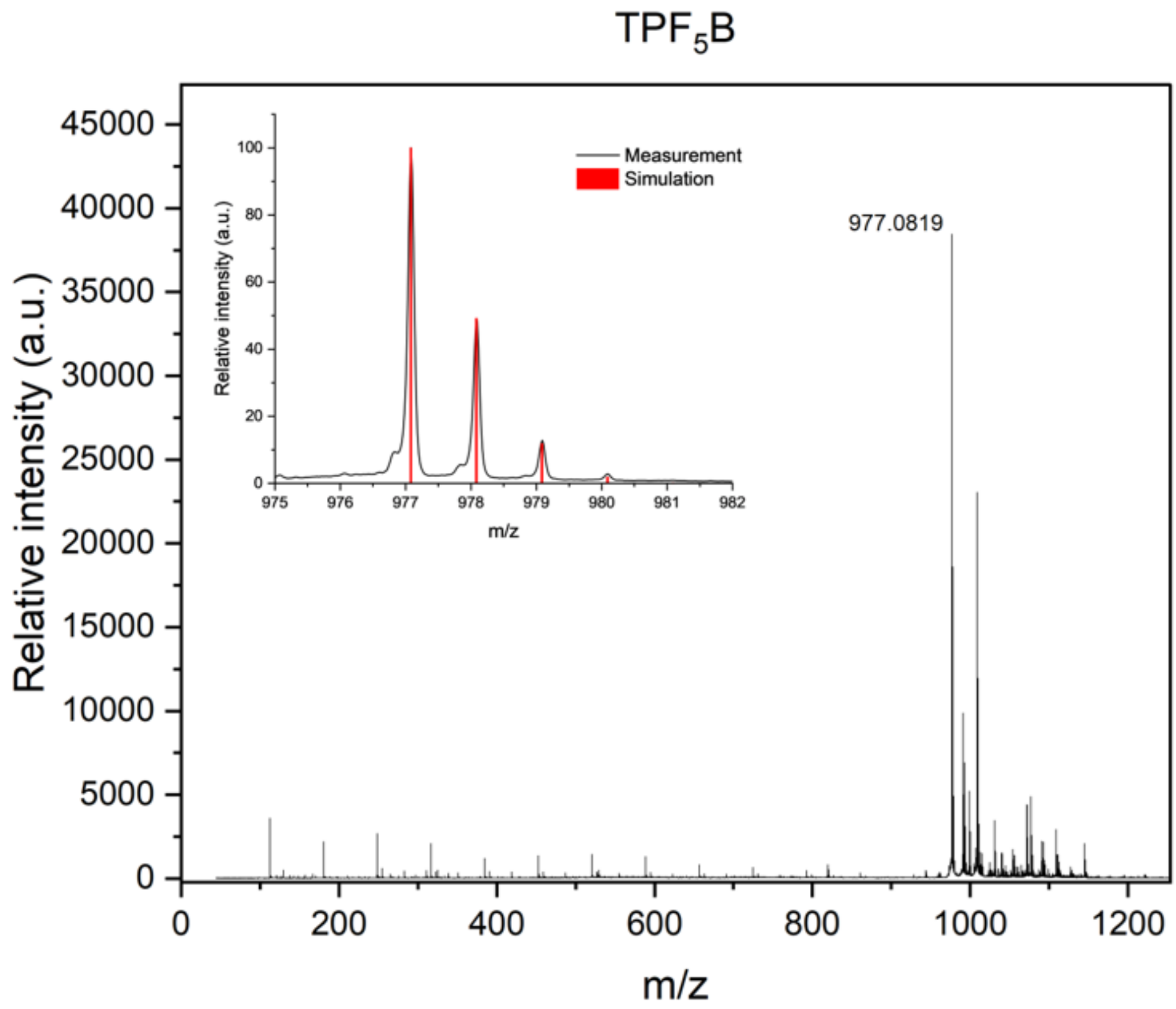

Figure S28. ESI- mass spectra obtained for TPF_5B (MW = 978.59), together with the isotope pattern simulated using ChemCalc.org.

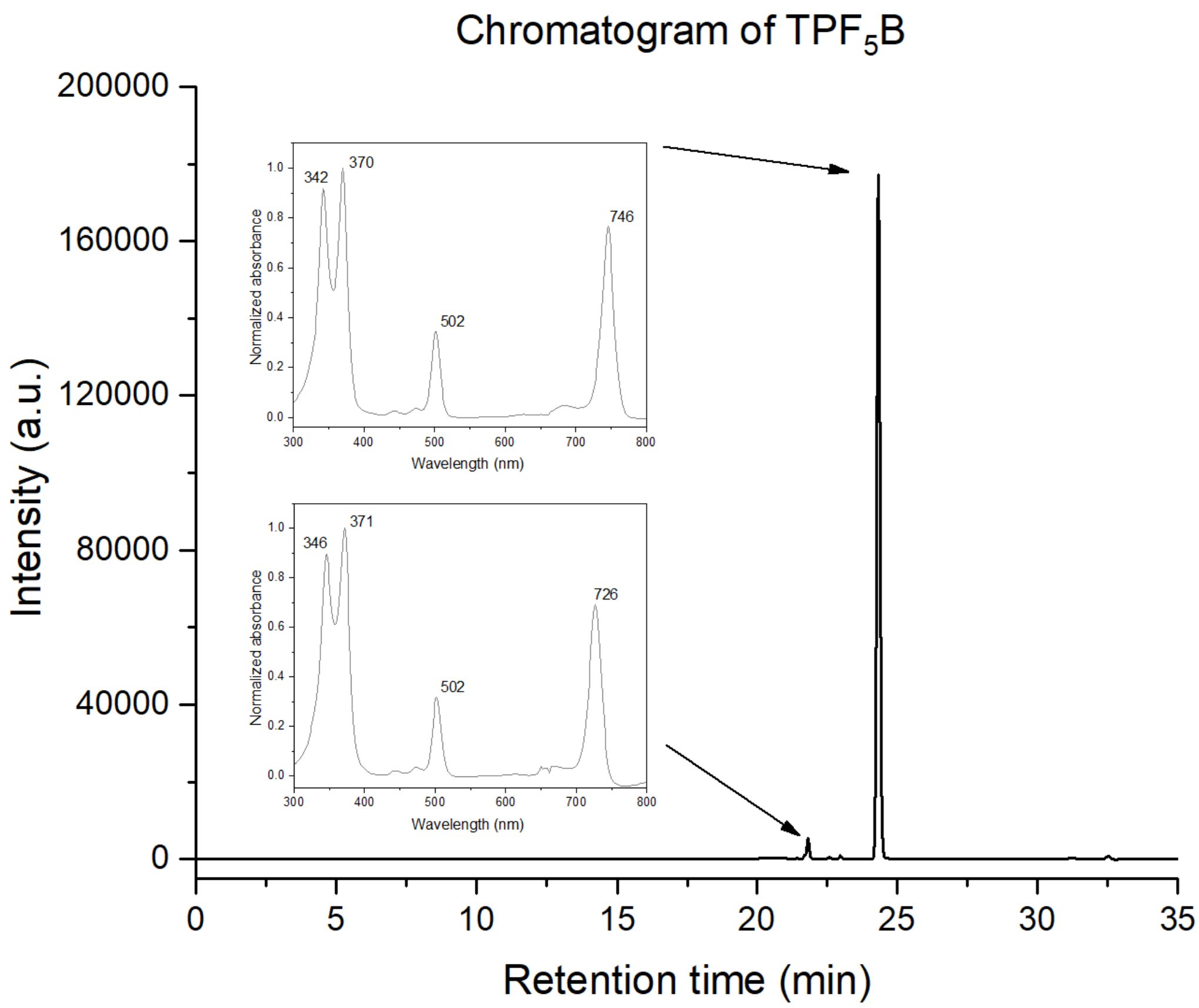

Figure S29. Analytical HPLC chromatogram and UV-Vis spectrum of lead compound TPF_5B. The main peak exhibited a retention time of t_R = 24.50 min. The purity of TPF_5B was 96.62%, calculated by peak-area normalization.

The purity of TPF_5B was assessed by high-performance liquid chromatography using a Shimadzu LC-2030C system equipped with diode array detection (DAD), and a C18 reversed-phase column (PerkinElmer, 5 µm, 150x4.6 mm). The mobile phase consisted of solvent A (0.1 M ammonium acetate in Milli-Q water) and solvent B (acetonitrile, MeCN). Elution was carried out using a gradient from 50% to 95% of solvent B over 35 min. The flow rate was maintained at 1.0 mL/min, and the injection volume was 10 µL. The retention time (t_R) of the main peak was 24.50 min. Compound purity was determined from the integrated peak areas of the HPLC chromatogram and was found to be 96.62%.

Abbreviations

2H-11 murine endothelial cell line

APF aminophenyl fluorescein

B16-F10 murine melanoma cell line

BALB/c mice

C-PDT cellular-targeted photodynamic therapy

CT charge transfer

CT26 murine colon tumor cell line

DAD diode array detection

DCM dichloromethane

DDQ 2,3-dichloro-5,6-dicyano-1,4-benzoquinone

DHE dihydroethidium

DLI drug-to-light interval

DLS dynamic light scattering

DMA dimethylanthracene

DMF dimethylformamide

DMPO 5,5-dimethylpyrroline N-oxide

DMSO dimethyl sulfoxide

EE% encapsulation efficiency

EPR electron paramagnetic resonance

EPR effect enhanced permeability and retention

ER endoplasmic reticulum

ESI electrospray ionization

FBS fetal bovine serum

EtOH ethanol

HOMO highest occupied molecular orbital

HPF hydroxyphenyl fluorescein

HPLC high-performance liquid chromatography

HRMS high resolution mass spectrometry

ICD immunogenic cell death

i.v. intravenous

$logD_{7.4}$, *n*-Octanol/PBS distribution coefficients at pH 7.4

ISC intersystem crossing

LUMO lowest unoccupied molecular orbital

MeCN acetonitrile

NIR near-infrared

NMR nuclear magnetic resonance

PBS phosphate buffered saline

PDT photodynamic therapy

PEO poly(ethylene oxide)

PPO poly(propylene oxide)

PS photosensitizer

ROS reactive oxygen species

SPF specific pathogen-free

TLC thin-layer chromatography

t_R retention time

UPR unfolded protein response

V-PDT vascular-targeted photodynamic therapy

ΔOD delta optical density

ε molar absorption coefficient

ε_{max} molar absorption coefficient

λ_{Em} emission wavelength

λ_{Ex} excitation wavelength

λ_{max} wavelength of band's maxima

τ_T triplet state lifetime

Φ_F fluorescence quantum yield

Φ_{PD} photodecomposition quantum yield

Φ_{Δ} singlet oxygen quantum yield
